# Dynamic Covalent Networks of Molecular Clusters for Hard and Impact‐Resistant Glass with Feasible Processability

**DOI:** 10.1002/advs.202524238

**Published:** 2026-03-02

**Authors:** Haiyan Xiao, Jia‐Fu Yin, Linjie Lan, Wei Liu‐Fu, Panchao Yin

**Affiliations:** ^1^ State Key Laboratory of Luminescent Materials and Devices & South China Advanced Institute for Soft Matter Science and Technology Guangdong Basic Research Center of Excellence for Energy & Information Polymer Materials South China University of Technology Guangzhou P. R. China; ^2^ School of Chemical Engineering and Light Industry Guangdong University of Technology Guangzhou P. R. China

**Keywords:** dynamic covalent bond, impact resistance, molecular cluster, molecular granular materials, organic glass, relaxation dynamics

## Abstract

The polymeric organic glass suffers from the intrinsic tradeoffs among mechanical strengths, hardness, and impact resistance from their chain topologies, limiting their extensive applications. Herein, a polymer‐free approach is developed for the fabrication of glass by crosslinking sub‐nm particle using dynamic covalent bonds. The sub‐nm polysilsesquioxane (POSS) particle is crosslinked by the dynamic boronic ester bonds when 1,4‐phenylenediboronic acid is doped. The high crosslink density with homogeneous distribution of POSS endows the fabricated films with high elastic modulus (1.79 ± 0.43 GPa), hardness (0.36 ± 0.03 GPa), and transparency (>89.5% transmittance). The dynamic feature of the network enables excellent impact resistance beyond typical polymers by showing highenergy dissipation capacity (258.14 J cm^−3^). The dynamic network also grants feasible processability that films with thickness as 1.3 µm can be fabricated through simple hot press. Moreover, due to the enriched B─O bonds and hydroxyl groups, high adhesive strengths as 8.20 ± 0.98 MPa for single lap shear strength on typical glass substrate can be achieved. This study provides new chemical systems to the design of glass with the capability to achieve balanced comprehensive performance in hardness, adhesiveness, (re)processability, and impact resistance besides its inherent high mechanical strength and transparency.

## Graphical abstract

Molecular granular material glass is fabricated by crosslinking sub‐nm POSS clusters with dynamic borate ester bonding while the materials combine high transparency, modulus, and hardness with superior impact resistance and (re)processability, offering a promising strategy for creating robust yet highly processable organic glasses.

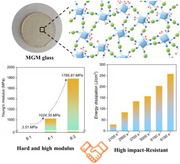

## 1 Introduction

Organic glass refers to a group of solid, transparent thermoplastic materials, and generally, they consist of macromolecular organic compounds, e.g., long‐chain polymers, that prevents the periodic packing of structural units and leads to the amorphous feature [1]. As opposed to the silicate‐based inorganic glass [2, 3], organic glass is characterized by the high impact resistance, light weight, and processability besides their intrinsic high mechanical strength, and they demonstrate broad applications in display [4], packaging [5], optical lenses [6], and fiber industries [7, 8, 9]. However, they suffer from the disadvantage of soft surface that is prone to scratching, low refractive indices, and low heat resistance, and thus, their extensive applications are limited [8, 10, 11]. Actually, it is the chain topology of polymers that sets ceiling for the comprehensive performance of organic glass. Typically, the mechanical properties of organic glasses stem from an intrinsic trade‐off: enhancing strength and rigidity reduces chain mobility and energy dissipation, thereby compromising toughness and impact resistance. Overcoming this compromise is key to developing high‐performance organic glasses that synergize these properties for demanding applications [12, 13, 14]. A comparable interplay is anticipated for surface hardness, toughness, and processability. To deal with the challenges, the glass nanocomposites have been developed by incorporating nanomaterials into glass matrix to reinforce the mechanical properties of organic glass [3, 15, 16]. Meanwhile, polymer chains can be tailored with certain functional groups for inter‐chain supramolecular interaction and the mechanical performance can be optimized [9, 14, 17]. Nevertheless, they are invariably constrained by the inherent trade‐offs between key properties, such as strength vs. toughness, processability vs. thermal stability, and surface hardness vs. bulk integrity. These limitations ultimately restrict their overall performance, particularly in meeting the demands of applications in extreme conditions. Consequently, it is highly desired to explore novel chemical systems other than the chain topology dominated polymer systems for the design of organic glass [18].

Molecular clusters (MCs) are a group of molecular nanoparticles with sizes ranging from sub‐1 to 10 nm, which is at the transition regime between small molecules and colloid nanoparticles (NPs) [19, 20, 21, 22]. Their monodispersed, well‐defined structures enable feasible and precise surface modification with required functional groups for tunable inter‐particle interactions. Benefiting from the ultra‐small sizes, the energy barriers for their diffusive dynamics are comparatively low in compared to colloid NPs and they are generally close to the ambient thermal fluctuation, granting broad windows for relaxation dynamics modulation to regulate the macroscopic mechanical properties [22, 23, 24, 25, 26]. Especially, materials with MC as building blocks have been confirmed to demonstrate tunable viscoelasticity [27, 28]. Among MCs, the outstanding performance of polyhedral oligomeric silsesquioxane (POSS) stems from its unique sub‐nanometer hybrid architecture. Versus small molecules, its rigid cage‐like core simultaneously enhances both strength and toughness while eliminating migration. In contrast to conventional nanoparticles, its molecular‐scale dimensions prevent aggregation, and its organic–inorganic design synergistically improves both processability and mechanical properties, which transcending the single‐function limitations of traditional fillers. As a group of emergent structural materials, molecular granular materials (MGMs) constructed from the dense packing of MCs exhibit resilient elasticity even at 100 K above their glass transition temperatures, which is distinct to the viscoelasticity of polymers [29, 30, 31, 32]. Therefore, the exploitation of MCs as structural constituents offers possible solutions to the intrinsic tradeoffs that limits the developments of polymer glass. Meanwhile, dynamic covalent chemistry (DCC) offers a compelling solution by introducing reversible cleavage and exchange capabilities into strong covalent bonds for enhanced toughness and processability [33, 34, 35, 36, 37, 38]. The integration of DCC into MGM systems provides a promising route to fabricate new types of organic glass with superior performance [39]. Herein, the MGM glass is designed from the crosslinking of sub‐nanosized POSS clusters with typical dynamic borate ester bonds and the crosslinking density can be feasibly varied for optimized glass performance. Their comprehensive performance in mechanical strength, hardness, impact resistance, adhesiveness, and (re)processability are evaluated while the hierarchical condensed structures and relaxation dynamics are probed using small angle scattering and broadband dielectric spectroscopy (BDS), respectively, for the understanding of the structure‐property relationship of the new MGM glass systems.

## 2 Results and Discussions

### 2.1 Molecular Design and Structural Characterization of MGM Glass

**FIGURE 1:**
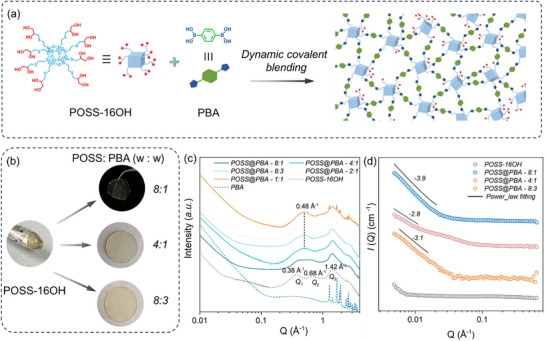
Design strategy and structural characterizations for MGM glass. (a) Schematical illustration for the preparation of POSS@PBA via dynamic covalent blending. (b) The physical appearances of POSS precursor and POSS@PBA composites. (c,d) SAXS and SANS data of POSS@PBA composites and precursors.

POSS cluster carrying 8 ortho‐dihydroxy groups (POSS‐16OH) is molecularly blended with 1,4‐phenylenediboronic acid (PBA) and the formation of typical boron ester dynamic covalent bonds ensure the construction of dynamic networks from the crosslinked POSS units (Figure 1a; Figure S1, details about the synthetic procedures are in provided in Supporting Information). By varying the mass ratios of PBA component, the mechanical properties can be broadly tuned as reflected from their physical appearances changed from soft elastomer with poor dimension stability to self‐standing rigid films (Figure S1; Figure 1b). The as‐prepared samples are termed as POSS@PBA‐x:y, where x:y denotes the mass ratio between POSS‐16OH and PBA (Table S1). Model reaction between POSS‐16OH and monofunctional phenylboronic acid is performed. The formation of B─O bonds can be further verified by the boron nuclear magnetic resonance (^11^B NMR) examinations from the shift of the characteristic boron peaks from −3.9 to −5.4 ppm (Figure S3). In FT‐IR studies, broad peaks ranging from 3700–3200 cm^−1^ assigned to the O─H stretching vibration band can be clearly observed for the raw materials while the corresponding peak intensity evidently decreases after the composition process, confirming the consumption of hydroxy groups during the borate ester reactions. Meanwhile, originated from alternation of bonding environment and reduced symmetry, the B─O vibration peak shifts toward higher wavenumbers in compared to PBA precursor. Moreover, the emergence of a new peak at 1306 cm^−1^, confirming the formation of B─O bonds in the network (Figure S4).

Small angle scattering methodologies provide broad spatial coverage characterization that enables deep insights into the hierarchical structures of the as‐formed hybrid networks. The small angle X‐ray scattering (SAXS) curve of the bulk of POSS‐16OH precursor shows three resolved scattering peaks while the scattering coherence at high *Q* (*Q* > 1 Å^−1^) is assigned to the form factor corresponding to the fine structures of discrete POSS skeleton (Figure 1c). At low *Q* range, the two peaks at 0.68 and 0.38 Å^−1^ are considered as the structure factor that reflect the spatial information regarding the packing of POSS clusters. The inherent chemical immiscibility between POSS cores and grafted hydroxy groups drives the asymmetrical distribution of surface structures and this directs to the formation of POSS supra‐nanostructures. The average separation distance between the POSS aggregates and inter‐POSS separation distance inside the aggregates can thereby be determined, d = 2π/*Q*, to be 1.65 and 0.92 nm, respectively. Moreover, the coherent scattering peaks at high *Q* (*Q* > 1.5 Å^−1^) is assigned to the form factor signals corresponding to the fine structures of a single, discrete POSS particle. For the POSS@PBA blends, these characteristic scattering features are all well preserved. The form factor analysis provides direct evidence for the structural integrity of the POSS cores after crosslinking reactions. Only one peak can be observed in the structure factor region since the crosslinking reaction drives the symmetric distribution of surface structures. This can be interpreted as the deconstruction of the POSS aggregates and subsequent homogenous distribution of POSSs inside the composites. POSSs remain as discrete particles and their surface i‐diol groups can therefore be fully exposed, which enlarges the possibility for covalent bonding with PBA components to yield robust cross‐linked networks. Due to the incorporation of PBA segment between the POSS particles, the increase of inter‐POSS separation distance (∼1.33 nm) can be rationally expected (Figure 4d). For the blends with high PBA loadings (POSS@PBA‐1:1 and 2:1), PBA cannot fully react and the remained PBA will phase separated out of the networks and finally crystallize, which is witnessed by the appearance of sharp diffraction peaks at high *Q* ranges. With respect to the small angle neutron scattering (SANS) data, sharp upturn at low *Q* range can be observed for the POSS@PBA hybrids and this is structurally originated from the network inhomogeneities with a length scale of nanometer scale (Figure 1d). The analysis further demonstrates that low‐PBA composites (e.g., POSS@PBA‐8:1) form homogeneous networks with smooth interfaces, as reflected in a Porod slope of approximately −3.9. With increasing PBA loadings (4:1 and 8:3), enhanced crosslinking kinetically traps compositional fluctuations, resulting in greater interfacial roughness and nanoscale heterogeneity. Meanwhile, neither scattering peak nor shoulder feature appears from medium to high *Q* regime (0.03–0.6 Å^−1^). This further validates the uniformity of local structures and homogenous distribution of the POSS units, consistent well with above SAXS analysis.

### 2.2 Processability and Optical Transparency of MGM Glass

**FIGURE 2:**
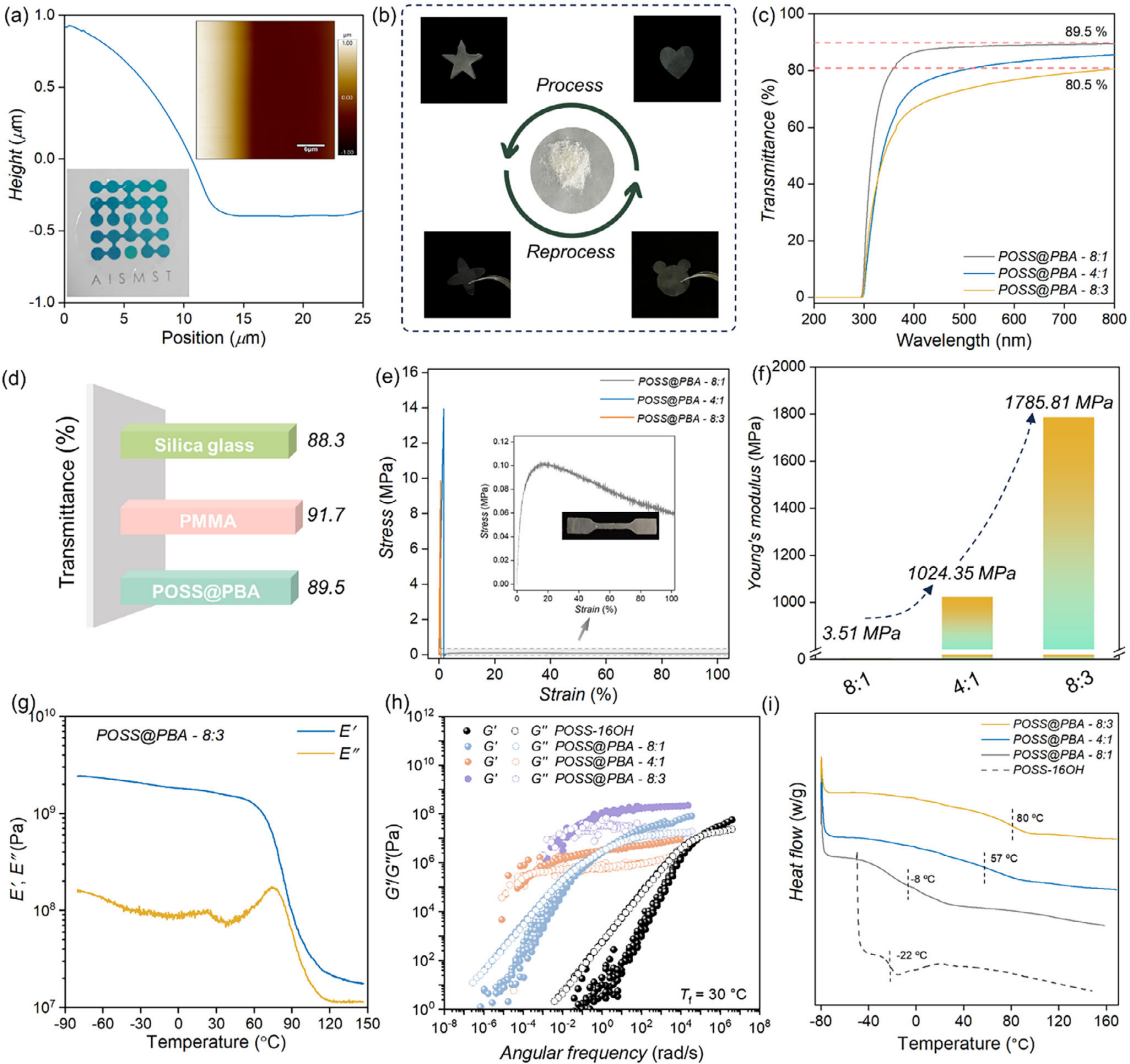
Processability, optical transparency, and mechanical properties of MGM glass. (a) AFM examination is conducted to measure the thickness of POSS@PBA. The inset is photograph of POSS@PBA film. With the copyright permission of “AISMST” logo from our institute. (b) POSS@PBA can be processed and reprocessed at mild conditions. (c) UV–vis spectra of POSS@PBA films. (d) Comparison of the transmittance of silica glass, PMMA and POSS@PBA. (e) Strain–stress curve for POSS@PBA with stretching rates of 10 mm min^−1^. The inset is image of POSS@PBA‐4:1. (f) The corresponding Young's modulus of POSS@PBA determined from the linear elastic region. (g) DMA data of POSS@PBA‐8:3. (h) Rheological master curves of POSS@PBA and POSS precursor with *T*_f_ of 30°C. (i) DSC thermograms of POSS@PBA at temperature ranging from −80°C to 170°C.

Colorless and optically transparent POSS@PBA films can be facilely prepared by harnessing the hot‐pressing approaches (Figure 2a). Taking POSS@PBA‐4:1 as an example, the raw powder is amenable to thermal manufacturing into customized shapes under mild conditions (10 MPa, 5 min, 60°C) (Figure 2b). Interestingly, ultra‐thin films with thickness at micro‐meter scale can be obtained by means of the hot‐pressing methodology and the film thickness can be feasibly regulated by optimizing the thermal processing parameters. In Figure 2a, ultrathin POSS@PBA film is demonstrated and AFM images reveal the smooth and flat surface without any wrinkles, whereas its vertical height is determined to be 1.3 *µ*m. UV–vis absorption tests are conducted and high transmittance of POSS@PBA films can be clearly demonstrated. POSS@PBA‐8:1 film with a thickness of 100 *µ*m maintains a transmittance exceeding 89.5% within the testing wavelength range (400–800 nm, Figure 2c; Figure S5), comparable to the that of the typical silica and polymethyl methacrylate (PMMA) glasses (Figure 2d). Slight reduction on the transparency is further observed for POSS@PBA‐4:1 and 8:3, most probably originated from the increased absorption contribution from the extended PBA networks. At higher PBA loadings, the phase separation and formation of PBA micro‐crystals bring undesirable multiple scattering effects. Therefore, the resultant opaque appearance of POSS@PBA‐2:1 and 1:1 impedes their extensive applications in the field of organic glass (Figure S6).

### 2.3 Mechanical Performance of MGM Glass

The mechanical properties of the MGM glass are comprehensively evaluated for their potential applications, which also sheds lights on the structure‐property relationship of this new types of organic glass (Figure 2e). The soft POSS@PBA‐8:1 shows good stretchability with Young's modulus of 3.51 ± 0.61 MPa (Figure 2f; Figure S7). The tensile stress starts with sharp increase and gradually decreases beyond a critical draw ratio (*ε* = 0.18). This reveals the deconstruction of the cross‐linking networks and it starts to flow under an external elongation field. The cross‐linking density can be feasibly regulated by varying the PBA contents. With the increasing of PBA loadings, POSS@PBA‐4:1 and 8:3 behave as rigid bulks, as manifested by their remarkably different mechanical response when subjected to the uniaxial tensions. The tensile strength is greatly improved accompanied with the compromise of materials’ overall fracture strain. High mechanical strength can be achieved, nearly 500 folds of increasements for POSS@PBA‐8:3 (1.79 ± 0.43 GPa) as compared to POSS@PBA‐8:1 (3.51 ± 0.61 MPa) (Figure 2f; Figures S7–S10 and Tables S2–S5). Through a simple remelting‐resolidification cycle, the POSS@PBA can be fully recovered to a glassy state with mechanical properties matching those of the virgin material, enabling efficient closed loop recycling and reprocessing (Figure S11). Dynamic mechanical analysis (DMA) is performed and the shearing modulus (*E′*) maintains at the GPa level within broad temperature range (−80°C–60 °C), consistent well with uniaxial tensile results (Figure 2g; Figures S12–S14). The observable significant mechanical reinforcement solidly reveals the dramatic enhancement of the crosslinking density of the dynamic covalent networks at higher PBA contents. Small amplitude oscillatory shearing (SAOS) test are carried out, which yields the rheological master curves by applying the time‐temperature superposition principle (Figure 2h; Figures S15–S25). The plateau moduli, positively correlated with the network cross‐linking density, are observed to increase significantly with increasing PBA contents. Higher cross‐link densities unsurprisingly give rise to the significantly slow down segmental dynamics, which can be directly reflected from the increase of both the glass transition temperature (*T*_g_) and rheological terminal relaxation time (Figure 2i; Figure S26).

**FIGURE 3:**
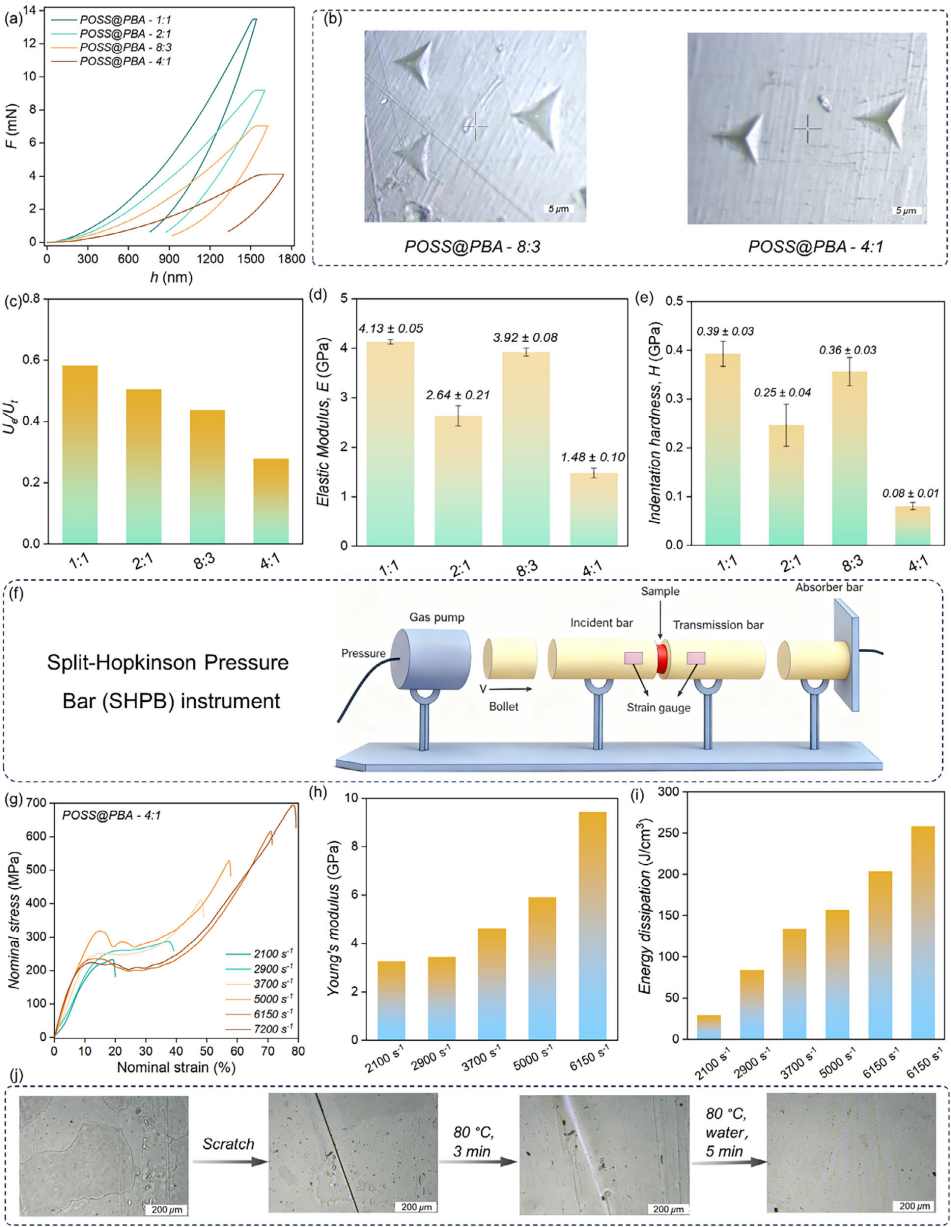
Mechanical properties and healing of MGM glass. (a) Indentation force‐displacement curves for POSS@PBA. (b) Optical microscope images of residual impressions after the nanoindentation experiment of POSS@PBA‐8:3 and 4:1, respectively. (c) Histogram of *U*_e_/*U*_t_ for POSS@PBA from averaged force‐displacement plots given in Figure 2a. (d) Elastic modulus and (e) Hardness of POSS@PBA from nanoindentation tests. (f) Schematic illustration of the SHPB system. (g–i) Compressive stress–strain curves at different strain rates, compressive strength, and energy absorption capacities of POSS@PBA‐4:1 from SHPB tests. (j) Optical microscopy images of the scratch and crack healing process of POSS@PBA‐4:1 (Scale bar = 200 *µ*m).

Nanoindentation analysis provides deeper insights into the mechanical performance and surface hardness of POSS@PBA glass systems. POSS@PBA‐8:1 is too soft for nanoindentation examinations, and therefore, the corresponding measurements are only performed for the other samples. The unloading path exhibits obvious hysteresis by comparing to the loading path, characteristic of mechanical properties intermediated between the viscous non‐Newtonian fluid and elastic solid (Figure 3a; Figures S27–S30). Distinct extended plateaus can be observed at the final stage of loading, which is particularly pronounced for POSS@PBA‐4:1 sample. This corresponds to the time‐dependent plastic flow, or creep, structurally originated from the cooperative rearrangements of the dynamic covalent networks. The indentation images provide further evidence for the plastic flow, in which evident surface deformation (a dent) can be detected after compression (Figure 3b). The elastic recovery (*U*_e_), a description of the ratio of the recovered work to the total work imparted during indentation loading, can thereby be quantified. The corresponding *U*_e_ value of POSS@PBA‐8:3 is larger than POSS@PBA‐4:1, indicative of the more pronounced elasticity resilience due to the higher cross‐linking density (Figure 3c). Data analysis based on the Oliver & Pharr method is exploited to decipher the mechanical fingerprint information. Both the two samples show superior mechanical stiffness with elastic modulus exceeding 1 GPa (Figure 3d). The elastic modulus exhibits a two‐fold increasement from 1.48 ± 0.10 GPa to 3.92 ± 0.08 GPa with increasing PBA loadings (Figure 3d; Tables s6–s8). The noticeable reinforcement of elastic modulus directly reflects the rise of cohesive energy among the structural units at higher network cross‐linking densities. While higher PBA content initially enhances modulus, excessive loading (e.g., in POSS@PBA‐2:1) becomes counterproductive (Figure 3d; Table S8). The steric crowding of boric acid groups hampers efficient boronic ester formation and network interlinking, resulting in a saturated or diminished cross‐linking density. This molecular‐scale impediment is the direct cause of the observed mechanical deterioration.

As the key factor to index the scratch resistance of organic glass, the indentation hardness (*H*) can be measured simultaneously to quantitatively describe material's resistance to the plastic deformation. Non‐monotonic dependence of *H* can be observed on the PBA loadings while the *H* value of POSS@PBA‐8:3 is calculated to be as high as 0.36 ± 0.03 GPa, which is very close to that of rigid polyimide and even aluminum alloys (Figure 3e; Table S8). The appreciable indentation hardness is structurally associated with the nano‐reinforcement effect from the high concentration of POSS units and the formation of robust cross‐linking networks. This impedes the deconstruction of the as‐formed networks and slip of the fragmented network segments, and therefore, the undesirable plastic deformations can be effectively prevented. A more detailed comparison is further carried out, which demonstrates good comprehensive mechanical properties of POSS@PBA‐8:3 that is comparable and even better than that of the previously reported both supramolecular and covalent organic glasses, and even rivals that of several conventional polymers (Tables S9–S11).

The excellent impact resistance is further confirmed with the help of typical split‐Hopkinson pressure bar (SHPB) tests (Figure 3f). POSS@PBA‐4:1 is capable of resisting the fast mechanical impact and even with the formation of macrocracks at strain rate > 4000 s^−1^, the specimen consistently maintains structural integrity without any debris ejection during the whole impact process (Figure S31). As depicted in Figure 3g, the input energy is dissipated through the deformation and the nominal stress increase with the compressive strains. The elastic moduli are positively correlated with the loading strain rates, characteristic of rate dependent mechanical behaviors (Figure 3h). Afterward, the curve dips down and typical yield behavior can be clearly detected (*ε* ∼10%), informing the transition from elastic to plastic deformation. Interestingly, pronounced strain hardening behaviors appears after the yield points only at high strain rate impacts (≥ 3700 s^−1^). The strain hardening and rate‐dependent response cooperatively impart unique mechanical adaptability to withstand the substantial compressive load under complex and extreme conditions. Moreover, a time‐dependent synergy orchestrates the impact response: fast hydrogen bonds act as sacrificial elements for instant energy absorption, while the slower boronic ester exchange drives post‐impact structural recovery through network reorganization and stress redistribution, thereby achieving exceptional high‐speed impact‐resistance (Figure 3i). Therefore, POSS@PBA‐4:1 can withstand a maximum compressive stress of 695 MPa, while showing an admirable impact toughness of 258.14 J cm^−3^ at 7200 s^−1^ (Figure S32). The dynamic impact performances of POSS@PBA‐8:1 and 8:3 are further evaluated and compared with POSS@PBA‐4:1. With regard to the load‐bearing capacity and impact toughness, POSS@PBA‐4:1 (408 MPa and 133 J cm^−3^ at 3700 s^−1^) exhibits the most excellent comprehensive properties, followed by POSS@PBA‐8:3 (420 MPa and 105 J cm^−3^ at 3900 s^−1^), and then POSS@PBA‐8:1 (170 MPa and 79 J cm^−3^ at 4000 s^−1^) (Figures S33–S38). The recycling and regeneration after materials’ failure can be feasibly achieved. Even after consecutive harsh reprocessing cycles, the mechanical properties are mostly stored, as reflected from the minimal degradation of the key parameters after recycling, including Young's modulus, indentation hardness, and impact resistance. Remarkably, despite its flexural modulus reaching the GPa level, POSS@PBA‐4:1 still exhibited impressive self‐healing behavior. The scratches of POSS@PBA are observed to be repaired within hours at temperature approaching the *T*_g_, due to the activated structural relaxation dynamics (Figure 3j; Figure S39). It is noteworthy that while POSS@PBA does not exhibit observable time‐dependent creep behavior, its boronate ester bonds undergo gradual hydrolysis in aqueous environments over time (Figures S40–S42).

**FIGURE 4:**
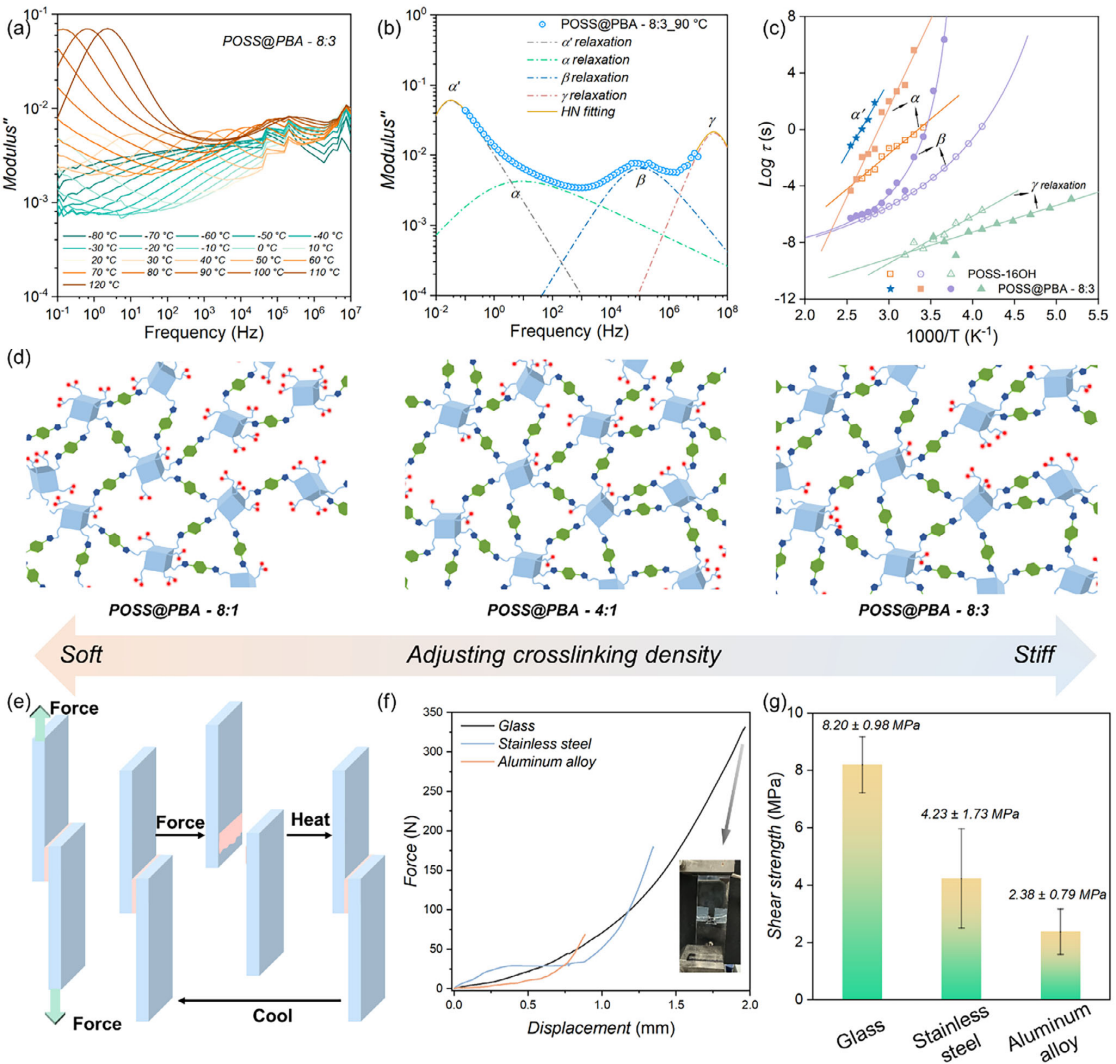
(a) The imaginary part of complex modulus for POSS@PBA‐8:3 from −80°C–120°C. (b) The imaginary part of complex modulus for POSS@PBA‐8:3 at 90°C and the experimental data are well fitted using a sum of four HN functions in the modulus expression. (c) The temperature dependence of relaxation time (τ) obtained by HN functions fitting. (d) The design principle of dynamic boron easter bonds engineering. (e) Schematic illustration of the lap shear procedure of POSS@PBA adhesives to the glass surface. (f) Peeling strength curves of POSS@PBA‐4:1 on the surface of glass, steel, aluminum. The inset is digital photograph of POSS@PBA‐4:1 on the glass surface. (g) Lap shear strength of POSS@PBA‐4:1 on substrates including glass, steel, aluminum.

The hierarchical dynamics are thereby probed by broadband dielectric spectroscopy (BDS) examinations to understand the physical origin of the unprecedented mechanical properties. BDS data of POSS‐16OH precursor is recorded and a total of three dielectric relaxations can be detected within our experimental temporal window, which can be structurally assigned to the local relaxation of grafted chains on POSS's surface (*γ*‐process), sub‐diffusive dynamics of POSS (*β‐* process), and cooperative motion of multiple POSSs (*α‐* process) (Figure 4c; Figures S43, S44). In contrast, apart from above three relaxation modes, an extra dielectric process (*α*′‐relaxation) rises and can be probed at higher temperature region (*T* > 353 K) for POSS@PBA‐8:3 (Figure 4a,b; Figures S45, S46). The corresponding relaxation time is quantified to be ∼60 s, which is comparable to that of the exchanging dynamics of nitrogen‐coordinated cyclic boron diester (NCB) as reported in previous research [40]. Therefore, we speculated that *α*′‐relaxation corresponds to the transesterification dynamics of boron ester networks for POSS@PBA‐8:3. It is interesting to observe some significant distinctions on the dielectric behaviors of *α‐* and *β‐*dynamics of POSS@PBA‐8:3 as compared to the POSS precursors (Figure 4c; Figures S47, S48). To be more specific, their characteristic relaxation times (*τ*_T_) are greatly improved while exhibiting much stronger temperature dependence (*T*‐dependence), which undoubtedly informs more restricted structural confinements and leads to the dramatically slow‐down relaxation dynamics. From our point of view, PBA undergoes boron ester reaction with POSS units to generate the dynamic covalent crosslinking networks. Meanwhile, the unreacted boron acid groups are prone to supramolecularly interact with POSSs via multiple hydrogen bonding, which acts as extra physical cross‐linking junctions to further stabilize the network structures. The dynamic covalent bonds work synergistically with the dense hydrogen interactions to create robust networks with the superior elastic modulus and indentation hardness. The dynamic hydrogen bonds can be disrupted at external mechanical stimuli and the disassociation‐association of them rapidly dissipates the input energy, bringing the capacity to withstand the fast impact. With respect to the primitive *γ*‐dynamics, the corresponding relaxation rate of POSS@PBA‐8:3 is much faster than that of POSS‐16OH precursor. The anomalous dynamics acceleration is most probably originated from the enlarged POSS–POSS distances due to the incorporation of PBA segments between the POSS units (Figure 4d). The bonused free volume at local region lowers down the energy barrier for structural relaxation, providing the molecular origin for the decreased activation energy (*E*_a_) of *γ*‐relaxation. Thanks to the persistence of the fast secondary relaxation, high energy damping ratio can be realized at harsh service conditions, expanding the adaptability and applicability when facing ultrahigh speed mechanical and wave impacts. The reversible nature combined with fast relaxation dynamics further enables feasible processing and regeneration after materials’ failures. The cost‐effective manufacturing and closed‐loop recyclability position this material as ideally suited to the current industrial focus on green production and atom economy.

### 2.4 Adhesive Property of MGM Glass

POSS@PBA demonstrate superior affinity to diverse hydrophilic surfaces, for instance, glass, steel, and aluminum substrates, projecting their promising application as robust adhesives. POSS@PBA is sandwiched between two adherent substrates and tensile forces are applied to pull apart the two surfaces (Figure 4e). In typical single lap shear tests, POSS@PBA show strongest adhesion to the glass surface with unprecedented shear strength up to 8.20 ± 0.98 MPa (Figure 4f,g; Figures S49–S51). Notably, failure occurred predominantly within the adhesive layer, indicating a cohesive failure mode. This observation confirms strong interfacial compatibility and suggests that the interfacial bond strength rivals or surpasses the material's intrinsic cohesive strength. The glass substrate at the joint area is observed to be completely fractured during tensile loading, further corroborating the exceptional adhesion capacity of POSS@PBA. From our point of view, the hydrophilic glass surfaces are characterized by an abundance of hydroxyl groups, which are inclined to interact with POSS@PBA layer via physical or chemical ways. The as‐formed boron ester cross‐linking nodes as well as the dense hydrogen bonding networks therefore contribute to the excellent adhesive performances. The boronic ester bonds undergo hydrolysis in the presence of water, which triggers the deconstruction of the POSS@PBA film under water environment. This can be validified from the fast decrease of the water contact angle within seconds (Figure S52). The unique stimulus‐responsive nature allows the on‐demand debonding and feasible removal, further expanding their application scenarios.

## 3 Conclusion

In summary, dynamic boronic ester chemistry is leveraged for the fabrication of novel MGM glasses from the cross‐linking of the sub‐nanosized POSS with PBA. The molar ratios between the two structural components dictate the cross‐linking density of the as‐formed reversible cross‐linked network, enabling feasible modulation of their physicochemical properties. The resulting MGM glasses exhibit high optical transparency as well as unprecedented mechanical performances. Due to the formation of robust three‐dimensional hybrid networks, the interaction potentials among the structural units are profoundly elevated, providing the molecular origin of the observable high elastic modulus and appreciable hardness. Even though the high ordered structural dynamics are restricted, the relaxations of local structures, e.g. association‐dissociation of the hydrogen bonds and the relaxation of surface structures on POSSs, are well‐preserved. Therefore, excellent resistance toward high‐speed impacts and feasible (re)processability at mild conditions can also be realized. The comprehensive properties of MGM glasses are comparable to and even better than that of the conventional polymeric glasses, projecting their extensive applications in the relative fields. More importantly, the polymer‐free design strategy is reasoned to be effective for resolving the long‐standing conflict between mutually exclusive mechanical properties. Our study opens new avenues for the rational design of organic glasses based on MC structural units. Given the wealth of available hybridization strategies, a brighter further of MGM glasses can be rationally expected. Moreover, beyond the specific system studied, the approach constitutes a generalizable platform. A vast chemical space of sub‐nanometer units (e.g., polyoxometalates, metallacages, and giant molecules), each precisely tunable geometry, interaction strength, and functionality. This can be leveraged to rationally encode performance into the glassy state from the bottom up, paving the way for a “property‐by‐design” paradigm in amorphous materials.

## 4 Experimental Section

### 4.1 General Materials

Octavinylsilasesquioxane POSS (V‐POSS, C_16_H_24_O_12_Si_8_) (98%, Meryer), 1‐thioglycerol (≥99%+, Adamas), 2,2‐dimethoxy‐2‐phenylacetophenone (DMAP) (≥ 99%, Aladdin), 1,4‐phenylenediboronic acid (PBA) (98%+, Adamas), methanol (CH_3_OH) (≥99.5%, Aladdin), tetrahydrofuran (THF) (≥99%, Aladdin), diethyl ether (≥99.5%, General‐reagent), deuterium oxide (D_2_O) (99.9%, Aladdin), dimethyl sulfoxide‐*d*_6_ (DMSO‐*d*_6_) (99.9%, Aladdin), methanol‐*d*_4_ (99.8%, Adamas), all organic solvents or monomers were used without further purification. Deionized water was obtained from the Ultra‐pure water system (Water Purifier, WP‐RO‐10B).

### 4.2 Preparation of POSS@PBA Blends

POSS‐16OH provides 8 ortho‐dihydroxy groups (briefly noted as *‐OH*) per molecule. PBA provides 2 reactive *─B(OH)_2_* groups per molecule. The theoretical optimal stoichiometry for forming a fully crosslinked network via cyclic boronic esters is [─*OH*]: [─*B(OH)_2_*] = 2:1. Based on this principle, POSS@PBA were fabricated with the following steps. POSS‐16OH (2.0 g) was dissolved in methanol (100 mL), and PBA was dissolved in methanol (100 mL) at a mass to POSS‐16OH ratio respectively of 1:8, 1:4, 3:8, 1:2, and 1:1. POSS@PBA samples were prepared by mixing the POSS‐16OH solutions and PBA solutions and stirring vigorously. Then the mixed solution was heated to 60°C to remove part of the solvent. The post‐annealed POSS@PBA was obtained after drying under vacuum at 60°C for 24 h. All used samples were the post‐annealed POSS@PBA unless otherwise noted.

### 4.3 Characterization Methods

Nuclear magnetic resonance (NMR), Fourier transform infrared spectroscopy (FT‐IR), small angle X‐ray scattering (SAXS), small angle neutron scattering (SANS), thermogravimetric analyses (TGA), differential scanning calorimetry (DSC), rheology, uniaxial tensile test, dynamic mechanical analysis (DMA), nanoindentation tests, broadband dielectric spectroscopy measurements (BDS), single lap shear test, atomic force microscopy (AFM), split‐Hopkinson pressure bar (SHPB) experiments, UV‐spectrophotometer, surface wettability measurements and self‐healing tests are employed for structural characterizations and mechanical property evaluations. Due to the limitation of this section, more details can be accessible in the attached supplemental information file.

### 4.4 Statistical Analysis

The uniaxial tensile test, nanoindentation test, and single lap shear test experimental data were presented as the mean ± SD (n = 3).

## Conflicts of Interest

The authors declare no conflicts of interest.

## Supporting information




**Supporting File**: advs74623‐sup‐0001‐SuppMat.docx.

## Data Availability

The data that support the findings of this study are available in the supplementary material of this article.
