# Supplementary material for: Dynamic Covalent Networks of Molecular Clusters for Hard and Impact‐Resistant Glass with Feasible Processability
Source: Adv Sci (Weinh). 2026 Mar 2;13(27):e24238. doi: 10.1002/advs.202524238 (PMC13170207; doi:10.1002/advs.202524238)
Supplement: Supplementary file 1 — Supporting File: advs74623‐sup‐0001‐SuppMat.docx. [file ADVS-13-e24238-s001.docx]

Supporting Information

Dynamic Covalent Networks of Molecular Clusters for Hard and Impact-Resistant Glass with Feasible Processability

Haiyan Xiao,^†^ Jia-Fu Yin,^†^ Linjie Lan, Wei Liu-Fu, and Panchao Yin*

**Table of contents:**

1. Materials and Solvents ………………………………………………20
2. Methods………………………………………………………………20
3. Experimental Section (Scheme S1 to S3) ……………………………24
4. Supplementary Figures and Tables (Figure S1 to S52, Table S1 to S11) …………………………………………………………………25
5. Reference ……………………………………………………………53

**1. Materials and Solvents**

Octavinylsilasesquioxane POSS (V-POSS, C_16_H_24_O_12_Si_8_) (98 %, Meryer), 1-thioglycerol (≥ 99 %+, Adamas), 2,2-dimethoxy-2-phenylacetophenone (DMAP) (≥ 99 %, Aladdin), 1,4-phenylenediboronic acid (PBA) (98 %+, Adamas), methanol (CH_3_OH) (≥ 99.5 %, Aladdin), tetrahydrofuran (THF) (≥ 99 %, Aladdin), diethyl ether (≥ 99.5 %, General-reagent), deuterium oxide (D_2_O) (99.9 %, Aladdin), dimethyl sulfoxide-*d*_6_ (DMSO-*d*_6_) (99.9 %, Aladdin), methanol-*d*_4_ (99.8 %, Adamas), all organic solvents or monomers were used without further purification. Deionized water was obtained from the Ultra-pure water system (Water Purifier, WP-RO-10B).

**2. Methods**

**2.1 Nuclear Magnetic Resonance (NMR) Spectroscopy.**

All the samples were dissolved in deuterium solvents with tetramethylsilane (TMS) as reference. ^1^H NMR has recorded on Bruker AVANCE II 500 spectrometer at 298 K.

**2.2 Fourier Transform Infrared Spectroscopy (FT-IR).**

FT-IR data of solid samples were recorded by Fourier transform infrared spectrometer (Bruker, VERTEX 33) in attenuated total reflection (ATR) mode. POSS-16OH and POSS@PBA were dispersed in anhydrous KBr powder, then piled into thin tablets for testing. FTIR spectra of samples were collected from the transmission model of FT-IR at 25 °C by subtracting the air background. The transmission data was converted to absorption data based on the equation: *A* = *Log (1/T)*, where *T* was the experimental transmissive value and *A* was the converted absorption value.

**2.3 Small Angle X-ray Scattering (SAXS).**

The POSS-16OH was sealed in a custom-made hollow U-shaped sample container with two layers of 3M tape. The POSS@PBA samples were sealed by two layers of 3M tape. The SAXS data were recorded on the beamline (BL-16B) of the Shanghai Synchrotron Radiation Facility (SSRF) with a Pilatus 2M detector. The wavelength of the incident X-ray beam is 1.23984 Å and the exposure time was set to 2 s to collect the structural information. Silver behenate (AgBh) was used as the standard sample for calibration. For all the samples, the background was the two layers of 3M tape. Fit2D software is used to convert two-dimensional scattering data into a one-dimensional scattering curve. In contrast, while the contribution of background scattering is subtracted, and the real one-dimensional scattering data is finally obtained.

**2.4 Small angle neutron scattering (SANS).**

SANS data were collected at China Spallation Neutron Source (CSNS). The wavelength of the incident neutron was 1.2 ~ 9.5 Å and the exposure time for each sample was 2 h to acquire the form factor of POSS-16OH. For the POSS@PBA samples, the samples were placed in a quartz container with a thickness of 1 mm. The POSS@PBA were exposed to the neutron beams and the scattered neutrons were detected by the area detector. The obtained two-dimensional data were then converted to one-dimensional data. The air background was subtracted to afford the final scattering curve.

**2.5 Thermogravimetric analyses (TGA).**

All samples (each sample 5-10 mg) heated from 30 to 800 °C with a heating rate of 10 °C /min under nitrogen atmosphere are performed on a Netzsch TG 209 F3 Tarsus®.

**2.6 Differential Scanning Calorimetry (DSC).**

Differential Scanning Calorimetry (DSC) was the determination of the thermal properties of materials using the NETZSCH DSC 214 instrument, the temperature range was -80 to 170 °C with a heating rate of 10 °C min^-1^. First, the procedure was performed at room temperature, with a heating rate of 10 °C min^-1^ ascending to 120 °C and holding for 5 minutes to eliminate any thermal history within the material. All the tests were performed under the nitrogen atmosphere.

**2.7 Rheology.**

A controlled-stress rotational rheometer (Anton Paar MCR-302) with parallel plate geometry was used to investigate the rheological properties of POSS@PBA. Amplitude sweeps with constant frequency (*ω* = 5 rad s^-1^) at 25 °C were performed to determine the linear viscoelastic region. Bearing this in mind, small amplitude oscillatory shear (SAOS) experiments were subsequently carried out to quantitatively evaluate the viscoelastic performances of different POSS@PBA samples within the frequency window ranging from 0.1 ~ 100 rad s^-1^ at 25 °C. To ensure the credibility of experiment data, the rheology experiments for each sample were repeated at least 2 times. The storage modulus (*G'*) and loss modulus (*G"*) were obtained from the above-mentioned dynamic rheology experiments. Following the principle of time-temperature superposition (TTS), master curves of *G*′ and *G*′′ of the samples were constructed at a reference temperature of 30 °C.

**2.8 Uniaxial tensile test.**

The tensile tests were carried out on a mechanical testing machine (Instron 6022, Instron Corporation, USA). All POSS@PBA are sized into dumbbell panels, with a length of 10 mm and width of 2 mm. The sample was stretched at a velocity of 10 ~ 50 mm min^-1^, until the sample fractured. Time interval between two loading cycles is 30 seconds.

**Statistical Analysis**

Young's modulus was derived from the initial linear-elastic portion of the uniaxial tensile stress-strain curves. The analysis procedure was as follows: For each curve, the linear region was identified, typically within the strain range of 0.05% to 0.1%. This region was selected prior to any visible yielding or non-linear deviation. Within this strain interval, a linear least-squares regression was performed on the stress-strain data points. The regression was carried out using the built-in fitting tools in *OriginPro*. The slope of the resulting best-fit line was directly reported as the Young's modulus (E) for that sample. The correlation coefficient (R²) of the fit was consistently >0.99, confirming the appropriateness of the selected linear region. This method, which utilizes multiple data points for fitting, provides a more stable and accurate modulus estimate than a two-point secant method, particularly for materials with slight initial non-linearity. The final modulus value for each composition represents the average and standard deviation calculated from a minimum of three independent specimens.

**2.9 Samples for Single Lap Shear Test.**

10 mm × 80 mm × 2 mm clean ordinary soda-lime glass substrates were washed with water and alcohol; 10 mm × 80 mm × 2 mm clean ordinary 304 stainless steel and aluminum substrates were washed with water, alcohol and acetone. The tested sample had a substrate (50 × 25 mm^2^) on top of the other, forming a small overlapping region where the glue samples (POSS@PBA films) were pasted. The periphery of the jointing region was masked with 3M tapes to avoid the overflown glue. The jointed substrates were heated at 80 ℃ for several minutes and stored at room temperature. The measurement was carried out at 1 mm min^-1^. The lap shear strength of the glue between two substrates was determined by dividing the maximum load by the contact area. The lap shear strength was presented as mean ± standard deviation.

**2.10 Dynamic Mechanical Analysis (DMA).**

Dynamic mechanical analyses (DMA) were performed using a DMA Q800 in the tensile test mode under the nitrogen atmosphere with a heating rate of 10 °C min^-1^ from -80 to 150 °C. For each experiment, all the samples are sized into tensile test standard. After the sample was placed under tension between a fixed and a moveable clamp, the heating chamber was closed to obtain the desired temperature range. The temperature was increased stepwise with a waiting time of 10 min for equilibration of the samples between the individual steps, and each sample was scanned at a given temperature range. All tests were conducted at a frequency of 1 Hz and a linear range of 0.50% strain. For each sample group, three separate measurements were taken to generate an average DMA response.

**2.11 Nanoindentation tests.**

Nanoindentation tests were performed by a nanoindentor (Anton Paar, Model TTX-NHT3) equipped with Berkovich diamond tip at 25 °C. The hardness and elastic modulus were quantified by Oliver-Pharr method and calculated from loading-displacement data. For each force loading, holding, and withdrawing cycle, the maximum displacement was 1500 nm, the loading and unloading rates were both at 100 mN/min and the holding time at the maximum displacement was 5 s.

**2.12 Broadband Dielectric Spectroscopy Measurements (BDS).**

Broadband dielectric relaxation spectroscopy (BDS) was performed using a Novocontrol Alpha ANB analyzer system with a Quatro nitrogen temperature controller. The samples were pressed at high temperature between two gold-plate copper sheets with a diameter of 8 mm and a thickness of 0.1 mm (100 ℃ for POSS@PBA - 8:3, 60 ℃ for POSS@PBA - 4:1 and 30 ℃ for POSS@PBA - 8:1). Fuse-silica fibers with a diameter of 50 or 100 *μ*m were used as spacers. Before the measurements, the samples were heated to make sure no solvent in the thin tablets. The measurements were conducted under the gradient frequency from 10^-1^ Hz to 10^7^ Hz under different temperatures.

**Dielectric Data Fitting**^[1]^

Through the broadband dielectric spectroscopy measurements, the hierarchical relaxation processes of POSS@PBA could be investigated. In order to remove the polarization effects in lower frequencies and high temperature, the complex modulus (*M** (ω)) is used to uncover the relaxation processes instead of complex permittivity (*ε**). Then: $M^{*}\left( \omega\right)=\frac{1}{\varepsilon^{*}}=M^{'}\left( \omega\right)+iM^{''}\left( \omega\right); M^{'}\left( \omega\right)=\frac{\varepsilon^{'}\left( \omega\right)}{{\varepsilon^{'}\left( \omega\right)}^{2}+{\varepsilon^{''}\left( \omega\right)}^{2}} and M^{''}\left( \omega\right)=\frac{\varepsilon^{''}\left( \omega\right)}{{\varepsilon^{'}\left( \omega\right)}^{2}+{\varepsilon^{''}\left( \omega\right)}^{2}}$. Two empirical relation functions are used to represent the complex modulus variations: 'Havriliak-Negami (HN)': $M^{*}\left( \omega\right)=M_{\infty}(1-\frac{1}{{(1+{(i\omega\tau_{M})}^{\alpha})}^{\beta}})$. The temperature dependence of τ for the main relaxation process are fitted as Arrhenius function ($\tau\left( T \right)=\tau_{0}exp(\frac{E_{a}}{kT})$, $E_{a}$ is activation energy, k is the Boltzmann constant and $\tau_{0}$ is a pre-exponential factor) and/or Vogel-Fulcher-Tammann (VFT) function ($\tau\left( T \right)=\tau_{0}exp(\frac{B}{T-T_{0}})$, *B* is a dimensional normalization factor, $T_{0}$ is the VFT temperature).

**2.13 Split-Hopkinson Pressure Bar (SPHB).**

Split-Hopkinson Pressure Bar (SPHB) instrument is comprised of a launching system, a strike bar, an incident bar, a transmission bar, an absorbed bar, and a data acquisition system. POSS@PBA - 8:1, POSS@PBA - 4:1 and POSS@PBA - 8:3 samples were pressed into a cylinder specimen with a diameter of 10 mm and a thickness of 5 mm. And the SPHB experiments was conducted under different speeds.

**Statistical Analysis**

The nominal stress *σ* is defined as the measured force divided by the cross-sectional area of the sample. The nominal strain *ε* is defined as the displacement divided by the gauge length of the sample. The fracture stress *σ*_b_ and strain *ε*_b_ are defined as the nominal stress and strain at the fracture point of the sample, respectively. The work of extension *W*_e_, characterizing the work required to fracture the sample per unit volume, was calculated from the area under the impact stress-strain curve until the sample fractured. The stretch ratio *λ* was obtained from the strain *ε* using the relationship *λ* = *ε* + 1. The compressive elastic modulus was calculated from the slope of the linear part of the stress-strain curve.

**2.14 Atomic Force Microscopy (AFM).**

The POSS@PBA samples were pressed at high temperature between two gold-plate copper sheets (100 ℃ for POSS@PBA - 8:3, 60 ℃ for POSS@PBA - 4:1 and 30 ℃ for POSS@PBA - 8:1), then the POSS@PBA solid films were obtained. The surface morphology of the samples was obtained using Cypher (Asylum Research, CA).

**2.15 Transmittance of POSS-16OH@PBA Films.**

The transmittance of a thin piece of POSS@PBA film was obtained using a UV-spectrophotometer (UV-1800, SHIMADZU). The thickness of all samples was ~100 μm.

**2.16 Surface wettability measurements.**

The static contact angle of the samples (∼100 μm in thickness) was measured using a contact angle goniometer (Biolin, Model KSV NIMA) at 25 °C. The droplet volume employed for static contact angles was 3 μL of liquid.

**2.17 Self-healing tests.**

To evaluate healing, cured samples of POSS@PBA - 8:1 and POSS@PBA - 4:1 healed for varying times under ambient conditions after dabbing the cut interfaces with water and reconnecting the two individual pieces along their freshly exposed interface. Healing was also attempted for all boronic ester samples over several days under ambient conditions without wetting the damaged samples prior to reconnecting. The samples of POSS@PBA - 8:1 and POSS@PBA - 4:1 were additionally tested for healing with heat in the absence of water at 80 °C for 5 min.

**3. Experimental Section**

**3.1 The synthesis of POSS-16OH**

The o-diol-functionalized polysilsesquioxane (POSS-16OH) was synthesized according to the literature.^[2]^ Octavinylsilasesquioxane POSS (V-POSS) (1.995 g, 3.14 mmol) and 5 mL 1-thioglycerol (5.96 mmol) were mixed with 30 mL THF. Then, 2,2-dimethoxy-2-phenylacetophenone (DMAP) (0.28 g, 11.2 mmol) was added to the solution. And the reaction mixture stirred at RT for 1 h under 365 nm UV light. THF was removed under reduced pressure. The resulting crude product was washed with icy diethyl ether for three times. The final product (3.26 g, 74.9%) was yielded after filtration and rotary evaporation. ^1^H NMR (500 MHz, methanol-*d*_4_): δ = 3.71 (m, 8H), 3.62 -3.47 (m, 16H), 2.71 - 2.58 (m, 32H). 1.05 (t, 16H).

**Scheme S1**. Reaction conditions: 1-thioglycerol, DMAP, THF, RT.

**3.2 The fabrication of POSS@PBA**

POSS-16OH provides 8 ortho-dihydroxy groups (briefly noted as *-OH*) per molecule. PBA provides 2 reactive *-B(OH)₂* groups per molecule. The theoretical optimal stoichiometry for forming a fully crosslinked network via cyclic boronic esters is [-*OH*] : [-*B(OH)₂*] = 2:1. Based on this principle, POSS@PBA were fabricated with the following steps. POSS-16OH (2.0 g) was dissolved in methanol (100 mL), and PBA was dissolved in methanol (100 mL) at a mass to POSS-16OH ratio respectively of 1:8, 1:4, 3:8, 1:2 and 1:1. POSS@PBA samples were prepared by mixing the POSS-16OH solutions and PBA solutions and stirring vigorously. Then the mixed solution was heated to 60 °C to remove part of the solvent. The post-annealed POSS@PBA was obtained after drying under vacuum at 60 °C for 24 h. All used samples were the post-annealed POSS@PBA unless otherwise noted.

**3.3 Recycling and thermal-assisted remolding of POSS@PBA**

POSS@PBA were reprocessed easily by hot-pressing, a widely-used polymer processing technique. POSS@PBA films were cut into pieces and loaded into a mold with the desired shape. The recycled POSS@PBA were remodeled at 60 °C under 10 MPa pressure for 5 min. The molded samples were transferred to an ambient condition and were carefully retrieved after being left at room temperature for 1 h.

**3.4 Preparation of POSS@PBA adhesive**

The samples were pressed at high temperature between two gold-plate copper sheets with a diameter of 8 mm and a thickness of 0.1 mm (80 ℃ for POSS@PBA - 8:3, 60 ℃ for POSS@PBA - 4:1 and 30 ℃ for POSS@PBA - 8:1). A series of POSS@PBA films containing different w/w ratios (8:1, 4:1 and 8:3) was prepared to understand the micro-structure and resulting mechanical properties of samples. The suffix number of samples’ notation refers to the mass ratio of PBA in 1 g of POSS-16OH during preparation; for example, POSS@PBA - 4:1 was prepared by blending 1 g of POSS-16OH and 0.25 g of PBA.

**4. Supplementary Figures and Tables**

**4.1 Supplementary Figures**


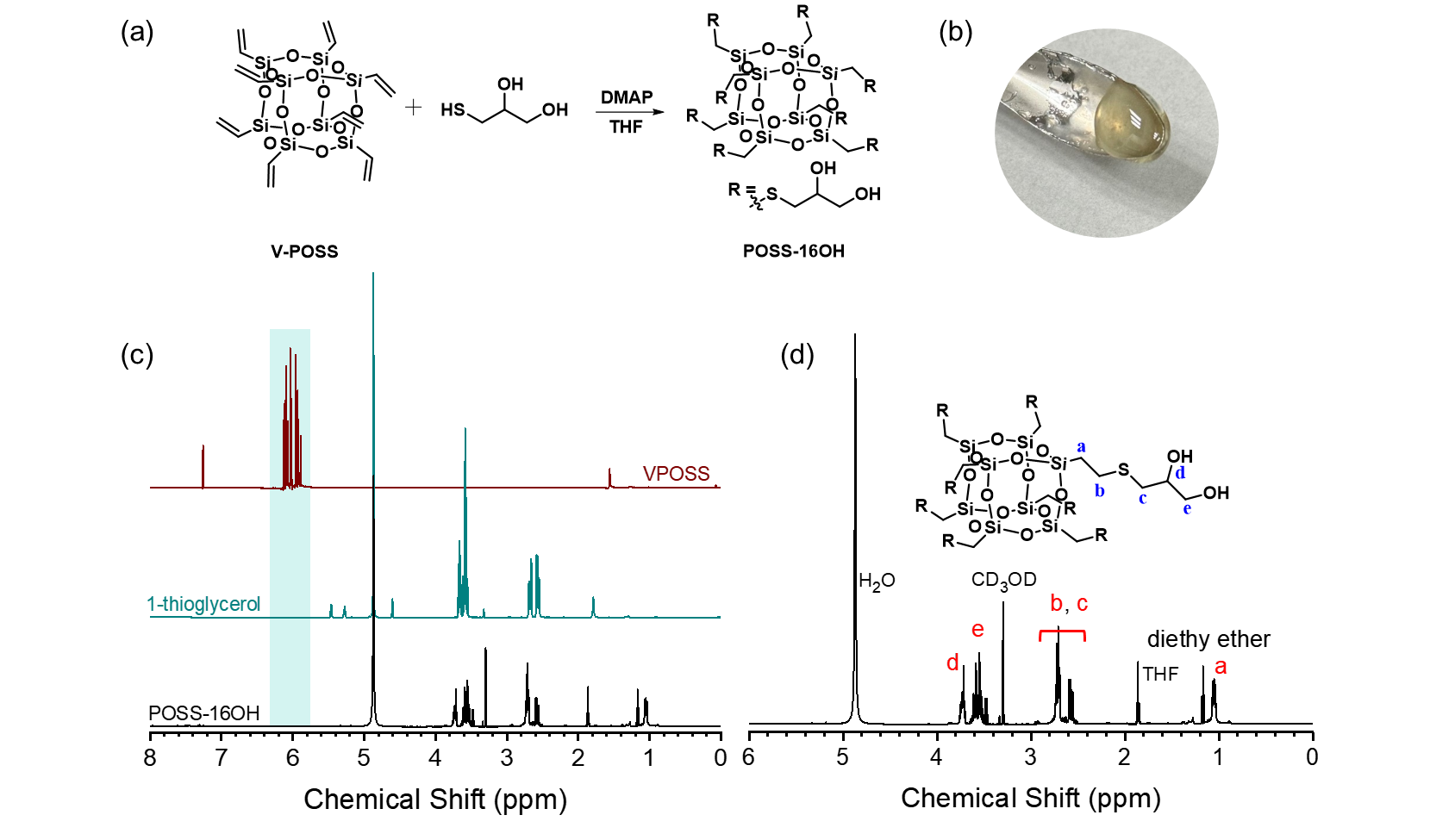


**Figure S1** (a) Scheme of the V-POSS by 1-thioglycerol to obtain POSS-16OH. (b) Photographs of POSS-16OH. (c) ^1^H NMR spectra of POSS-16OH and 1-thioglycerol in CD_3_OD, and VPOSS in CDCl_3_ at 298 k. (d) ^1^H NMR spectra of POSS-16OH in CD_3_OD at 298 k.


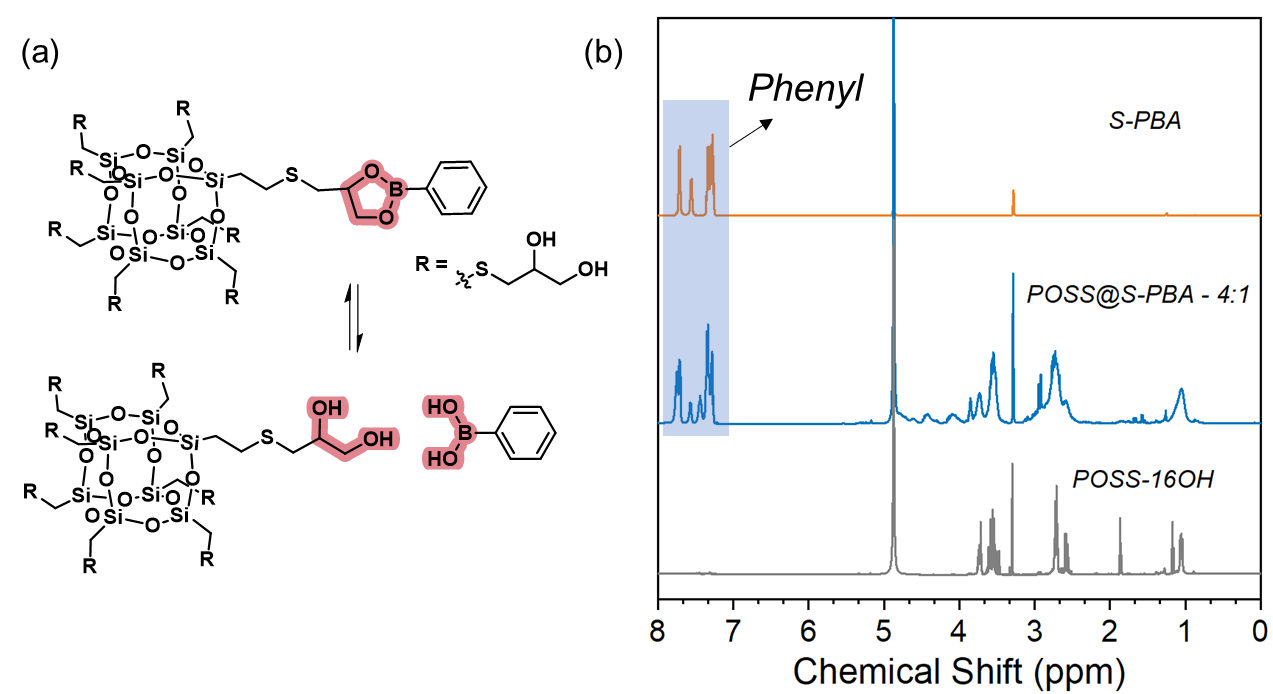


**Figure S2** Dynamic exchange reaction between POSS-16OH and monofunctional phenylboronic acid (S-PBA). (a) Transesterification reaction between POSS-16OH and phenylboronic acid. (b) ^1^H NMR spectra of POSS-16OH, POSS-16OH@S-PBA and phenylboronic acid in CD_3_OD-*d*_4_ at 298 k, respectively.


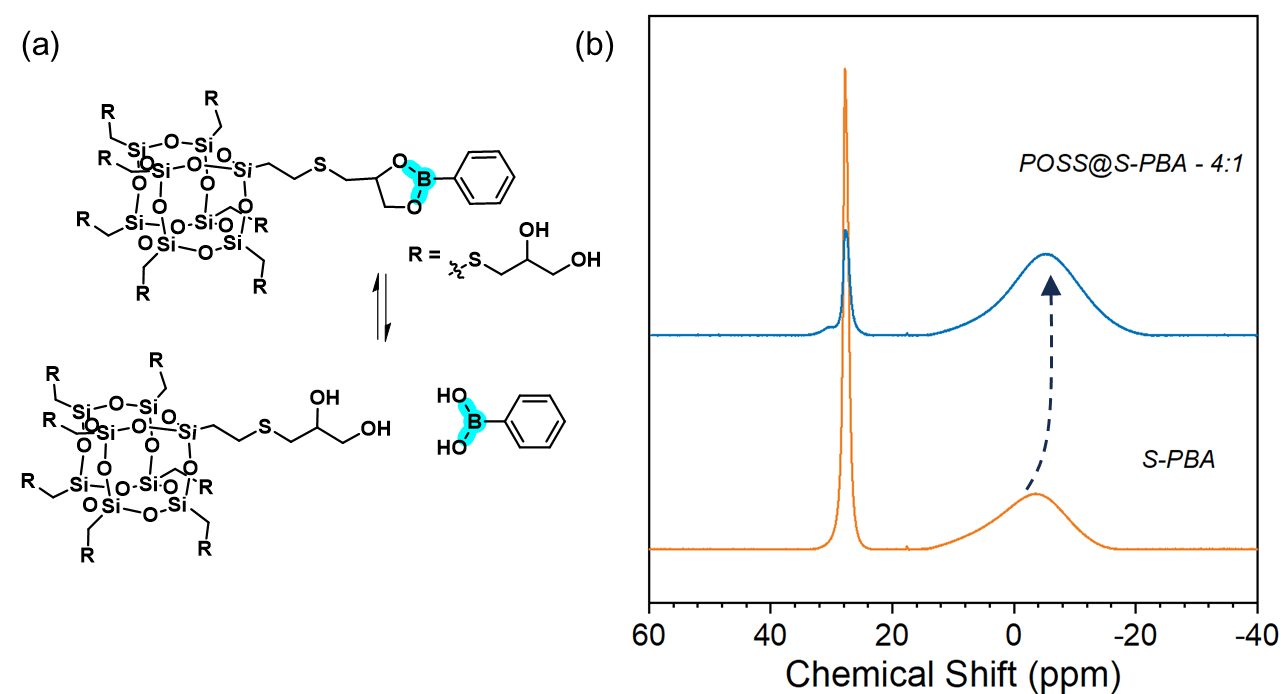


**Figure S3** Dynamic exchange reaction between POSS-16OH and monofunctional phenylboronic acid (S-PBA). (a) Schematic diagram for concept verification of transesterification reaction between POSS-16OH and phenylboronic acid. (b) ^11^B NMR spectra of POSS-16OH@S-PBA and phenylboronic acid in CD_3_OD-*d*_4_ at 298 k, respectively.

**
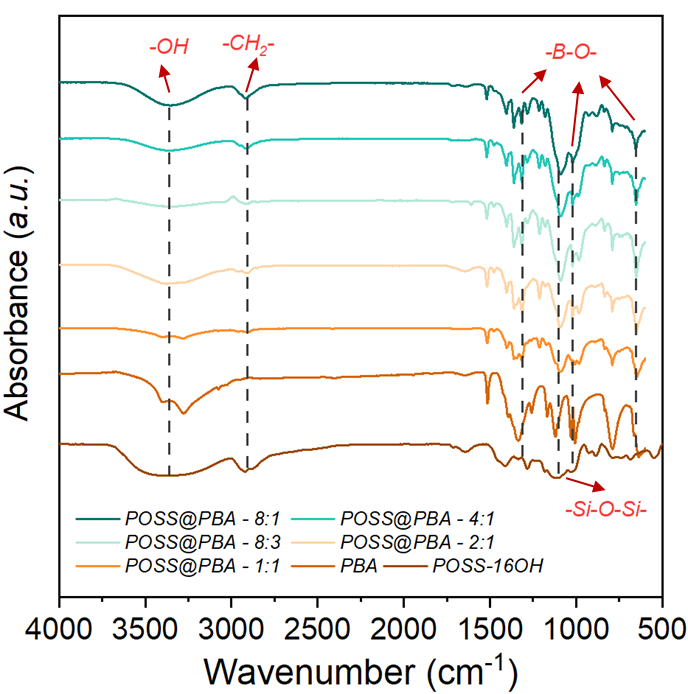
**

**Figure S4** FTIR spectra of POSS-16OH, PBA and POSS@PBA composites.


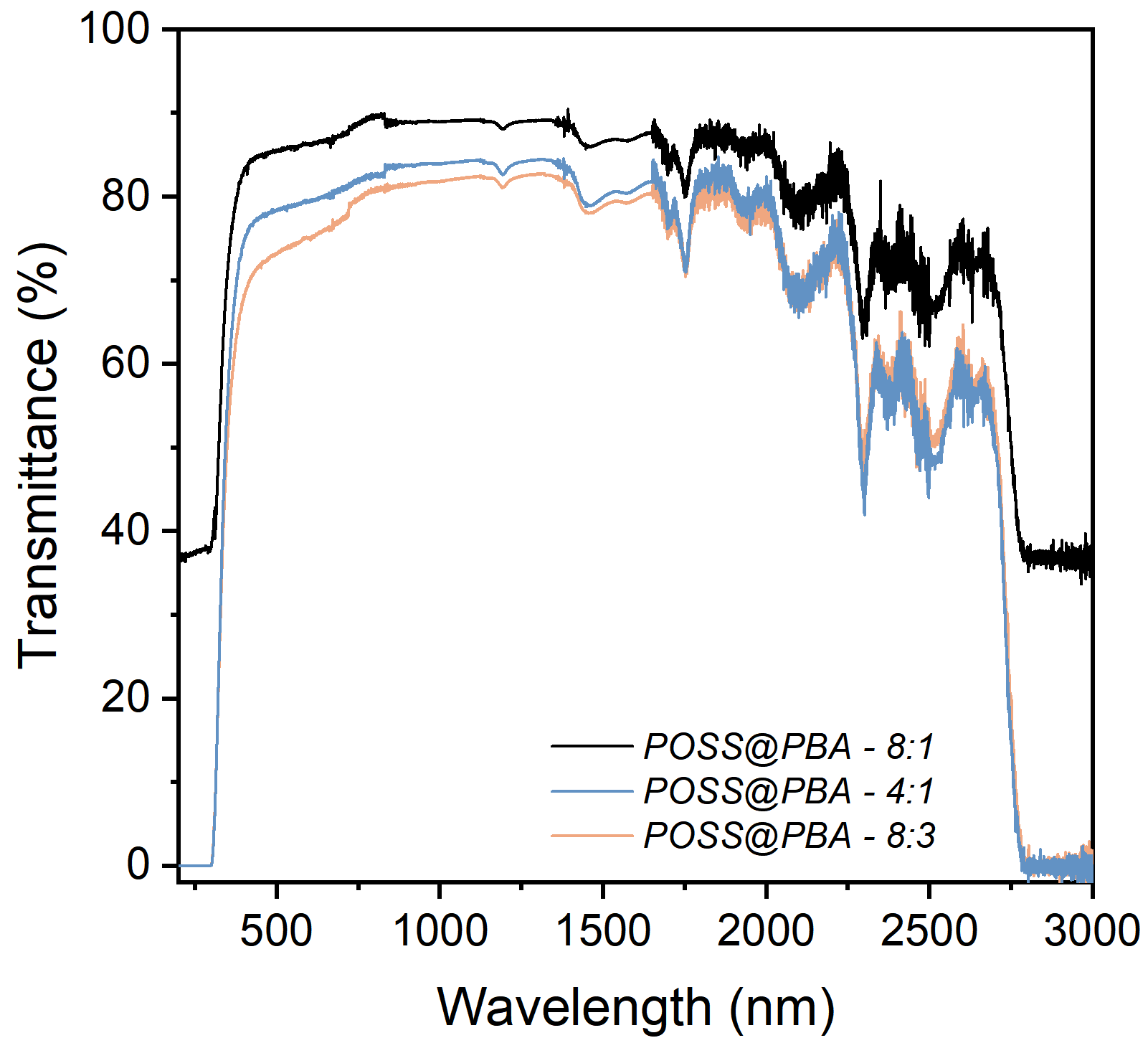


**Figure S5** UV-vis spectra of POSS@PBA films (400 - 3000 nm).


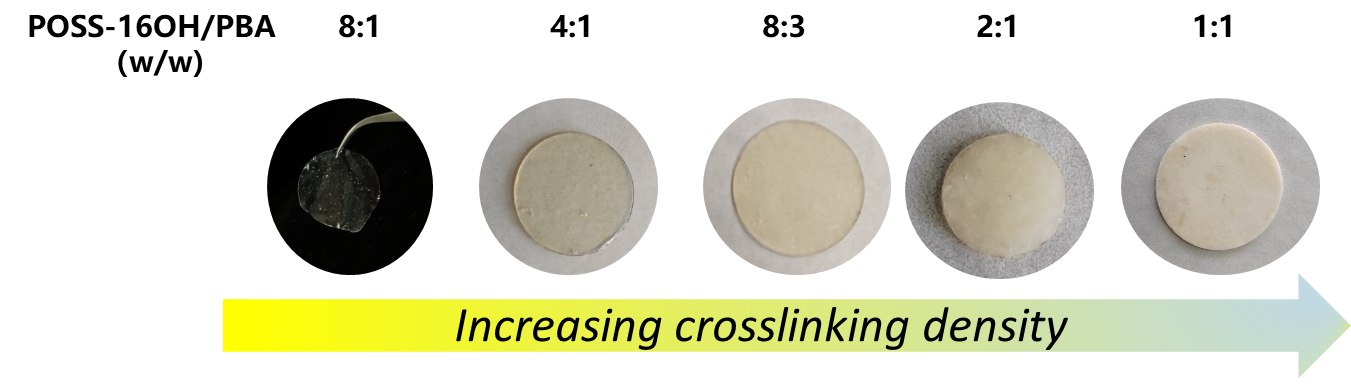


**Figure S6** The physical appearances of different POSS@PBA composites.


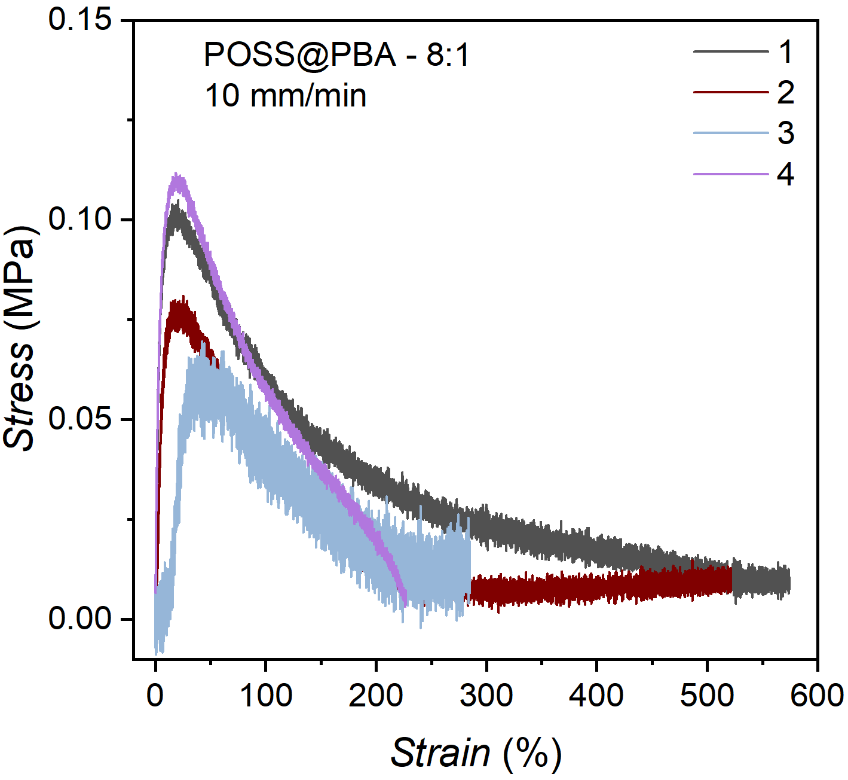


**Figure S7** Strain-stress curves for POSS@PBA - 8:1 at 10 mm min^-1^.


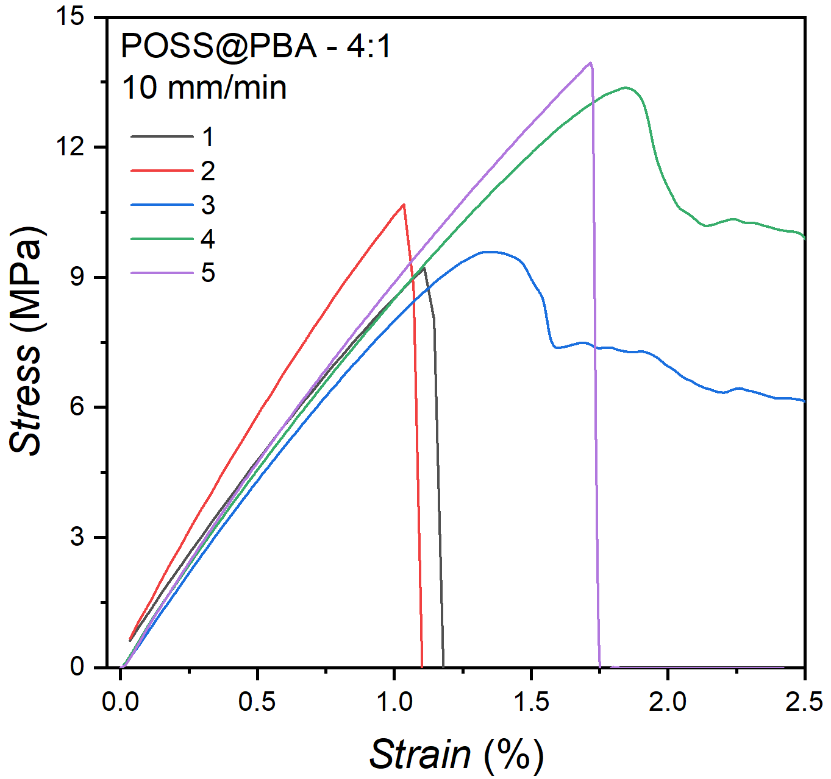


**Figure S8** Strain-stress curves for POSS@PBA - 4:1 at 10 mm min^-1^.


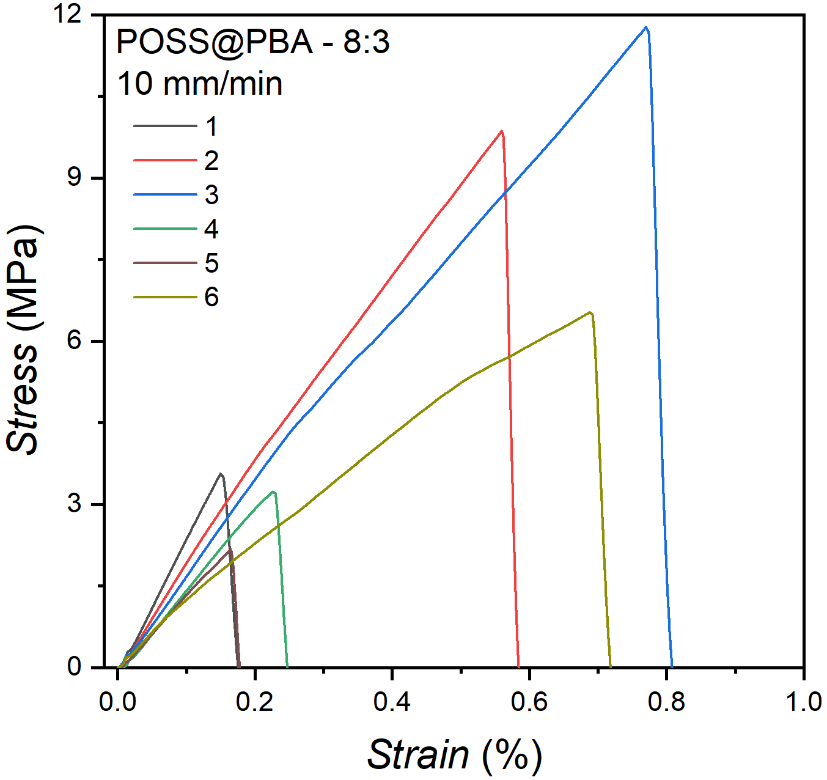


**Figure S9** Strain-stress curves for POSS@PBA - 8:3 at 10 mm min^-1^.


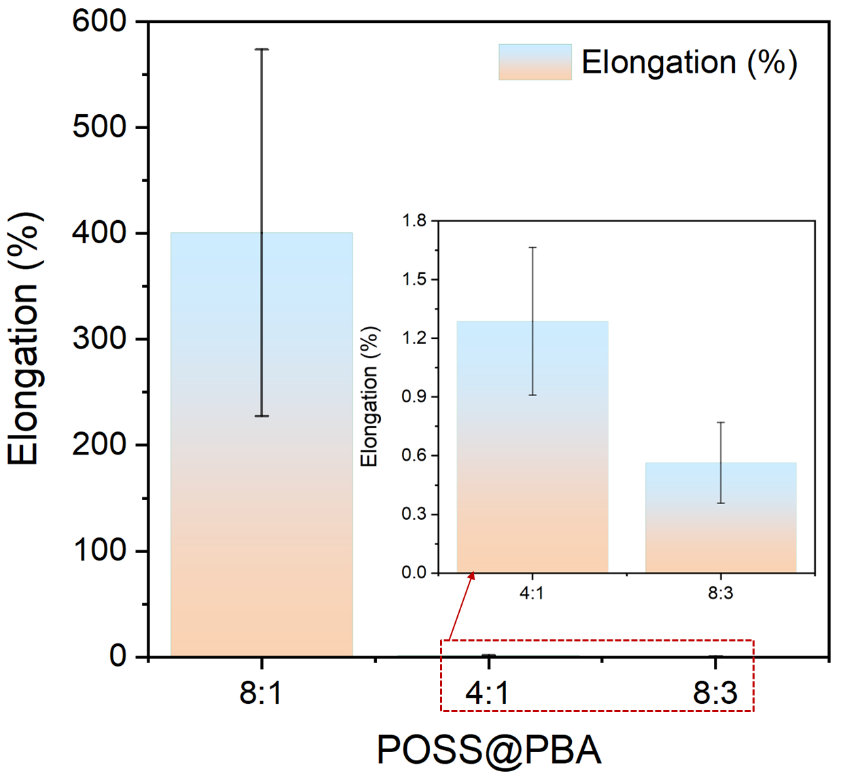


**Figure S10** Elongation of POSS@PBA obtained by uniaxial tensile test.


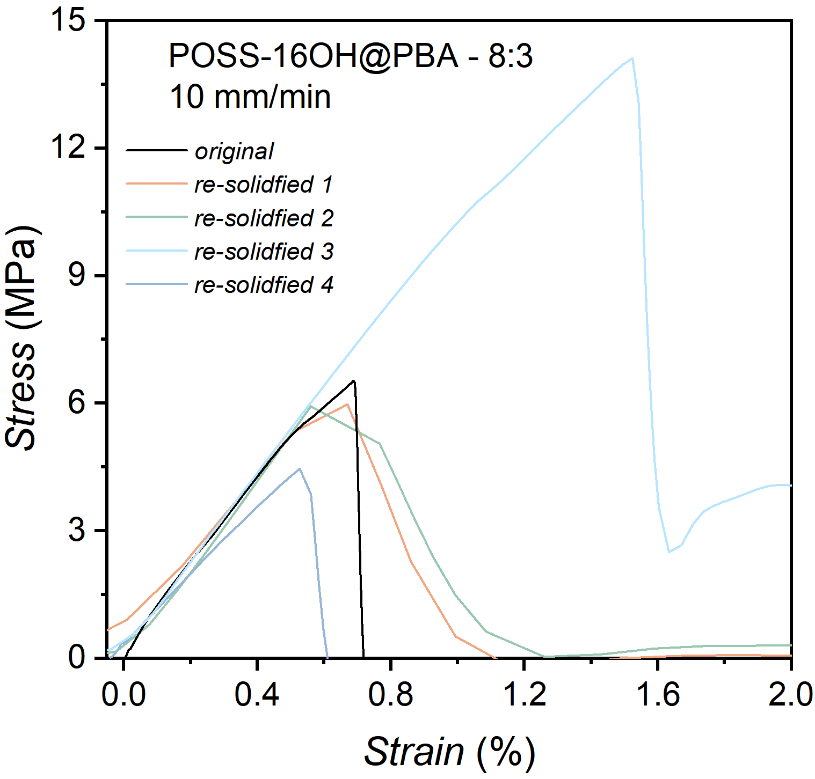


**Figure S11** Strain-stress curves for POSS@PBA - 8:3 at 10 mm min^-1^ before and re-solidified.


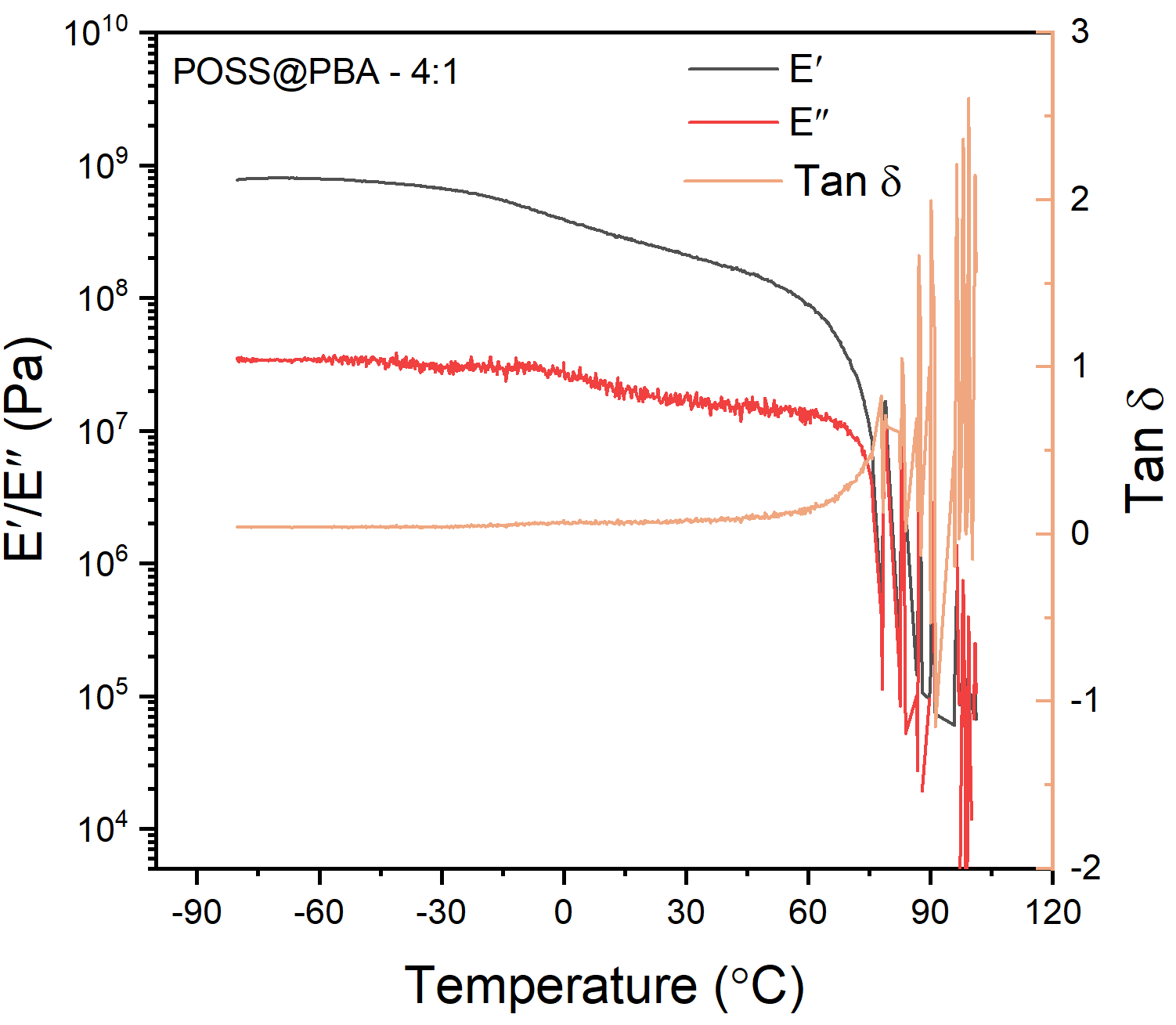


**Figure S12** DMA studies of POSS@PBA - 4:1.


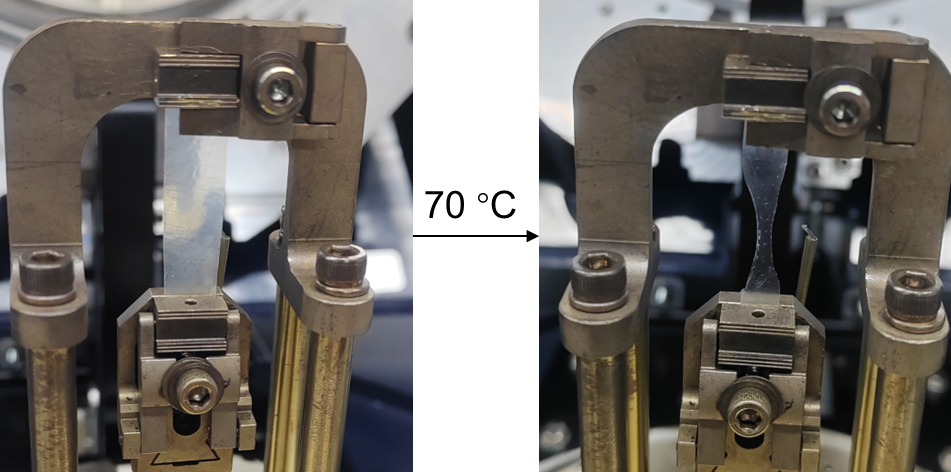


**Figure S13** The photographs for DMA tests of POSS@PBA - 8:3.


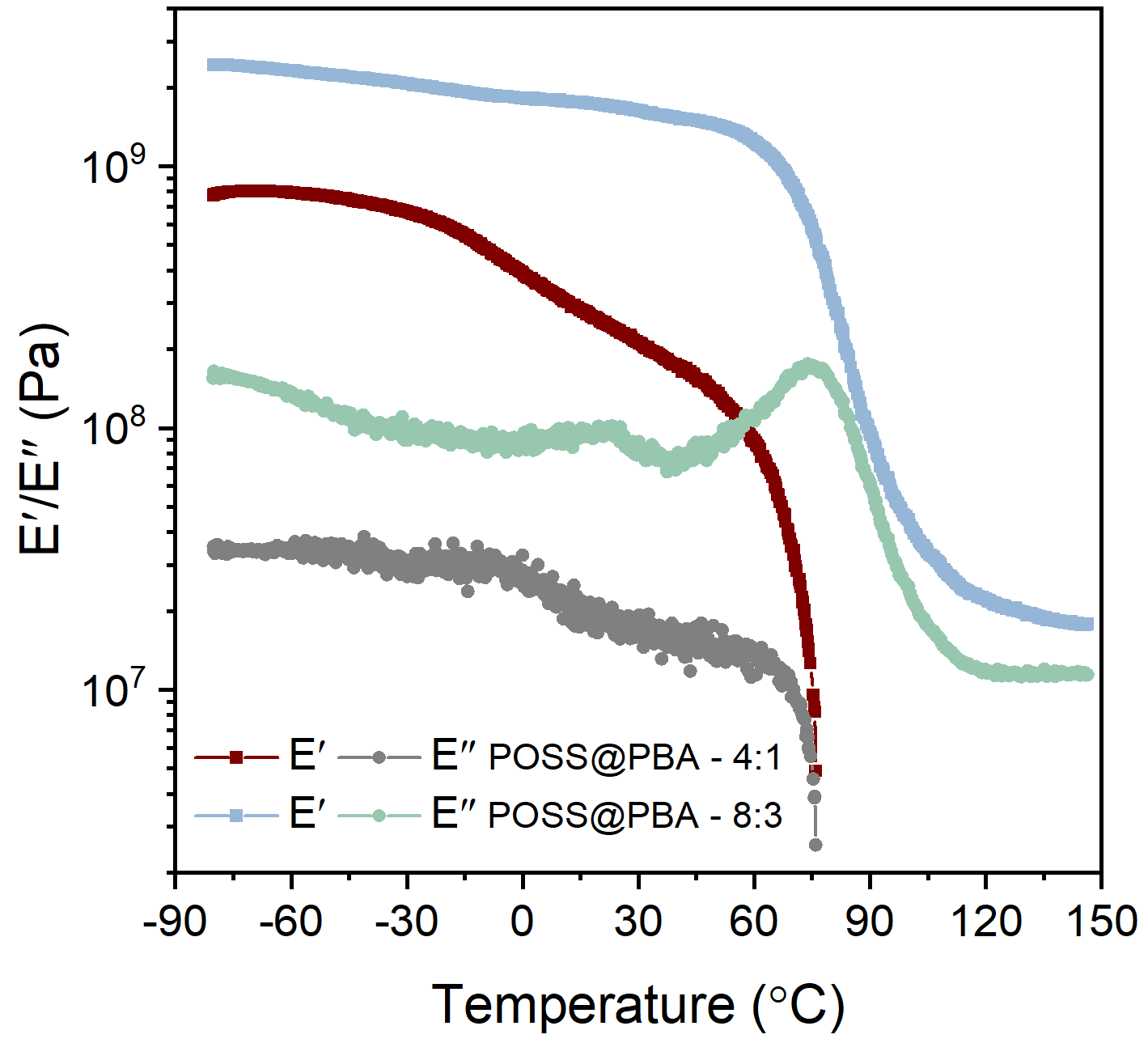


**Figure S14** The comparison of DMA studies of POSS@PBA - 4:1 and POSS@PBA - 8:3.


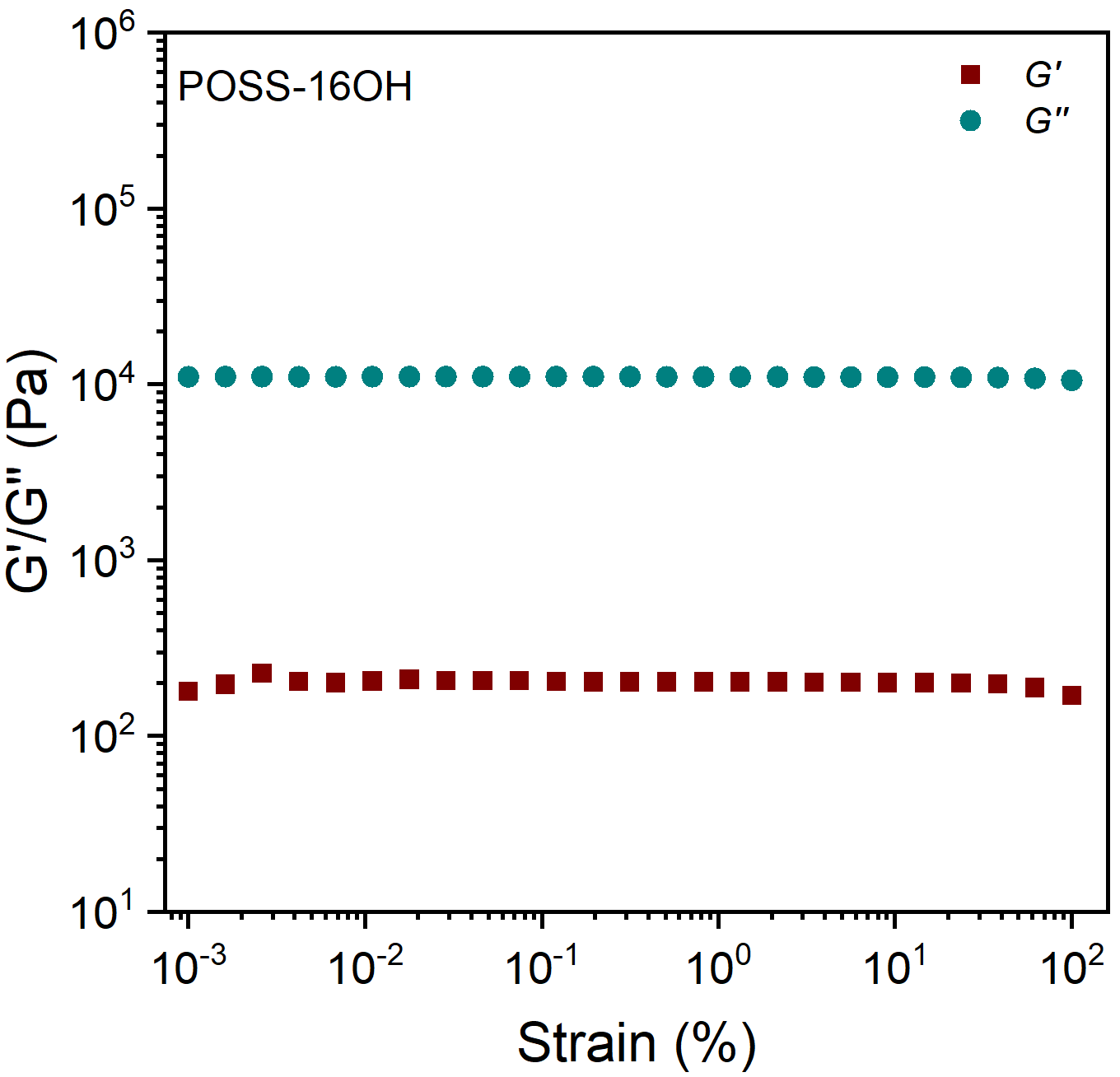


**Figure S15** Amplitude sweep (25 ^o^C, *ω* = 5 rad s^-1^) of POSS-16OH.


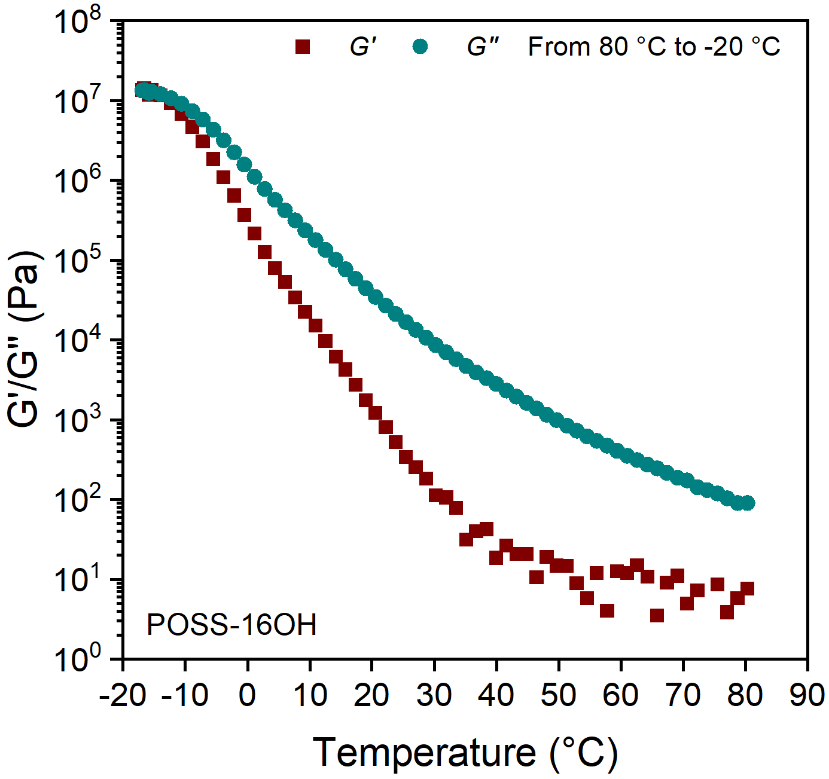


**Figure S16** Representative temperature sweep data of POSS-16OH.


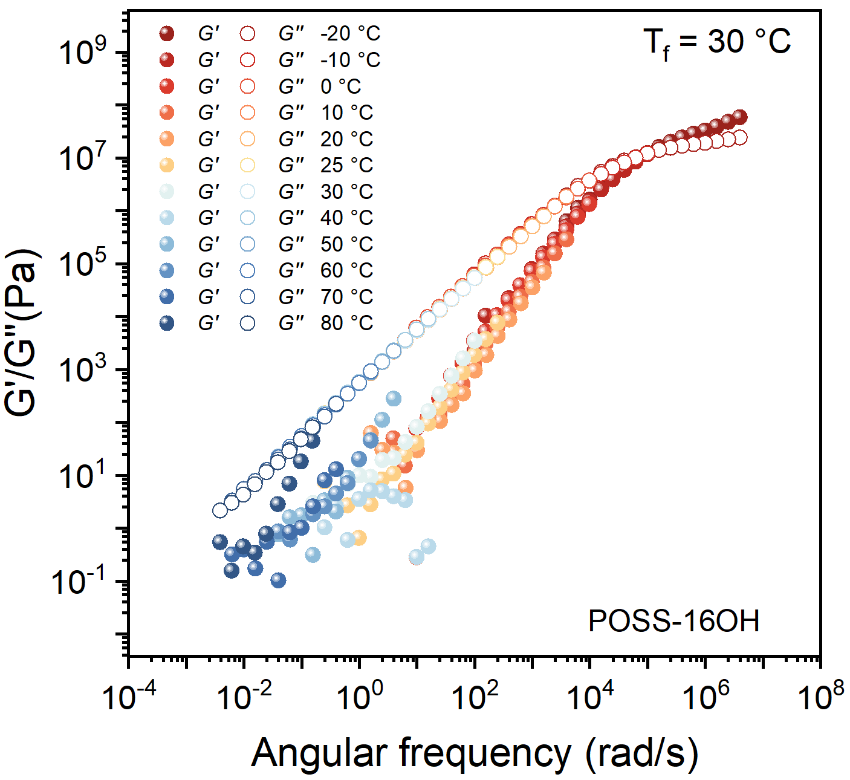


**Figure S17** Master curves of POSS-16OH at *T*_f_ of 30 °C from SAOS experiment.


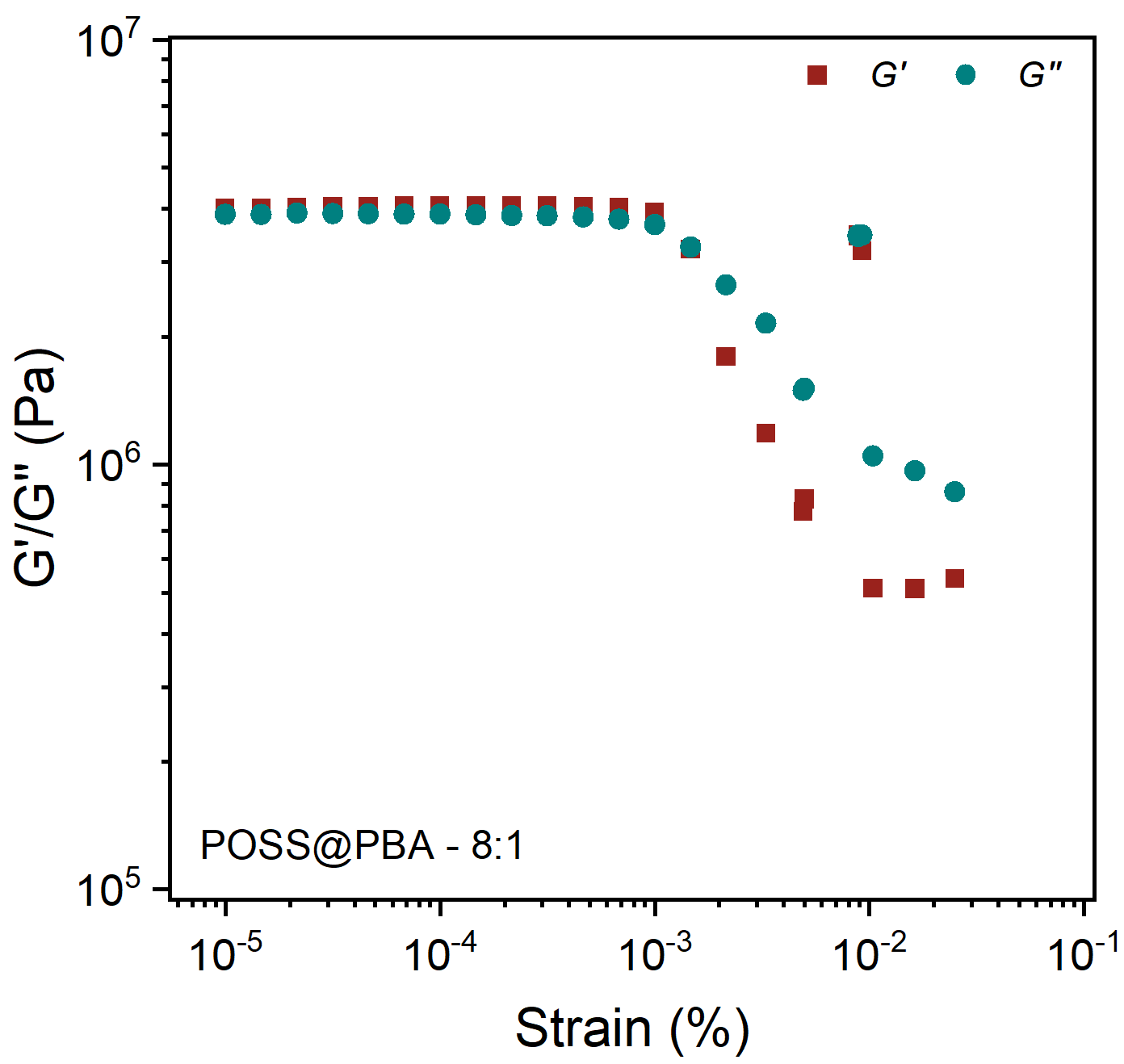


**Figure S18** Amplitude sweep (25 ^o^C, *ω* = 5 rad s^-1^) of POSS@PBA - 8:1.


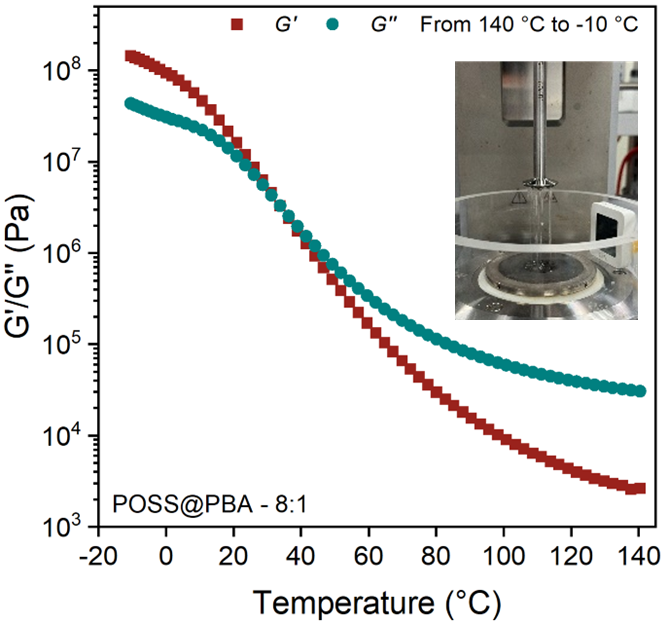


**Figure S19** Representative temperature sweep data and photograph of POSS@PBA - 8:1. The insets is photograph of POSS@PBA - 8:1.


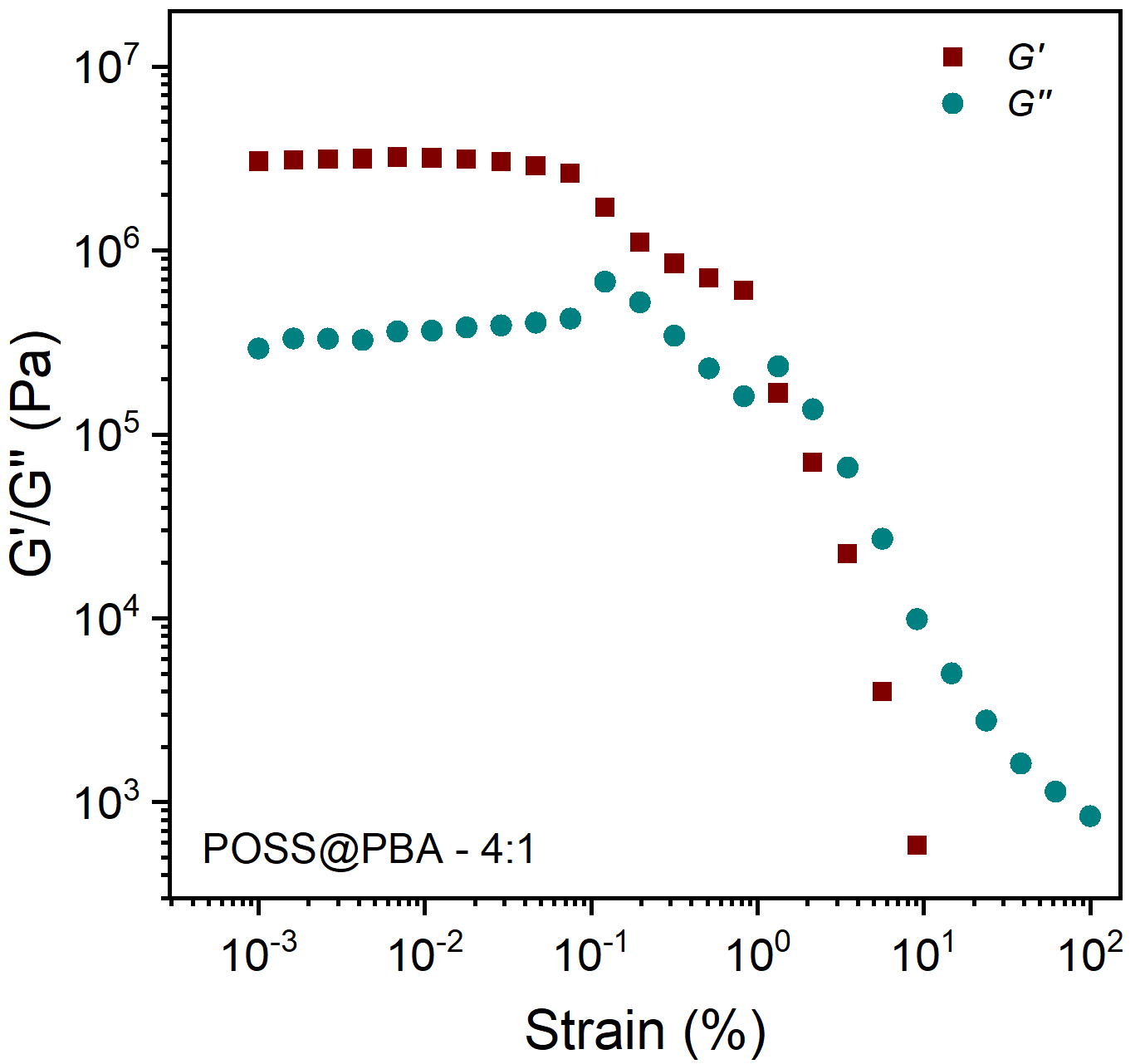


**Figure S20** Amplitude sweep (25 ^o^C, *ω* = 5 rad s^-1^) of POSS@PBA - 4:1.


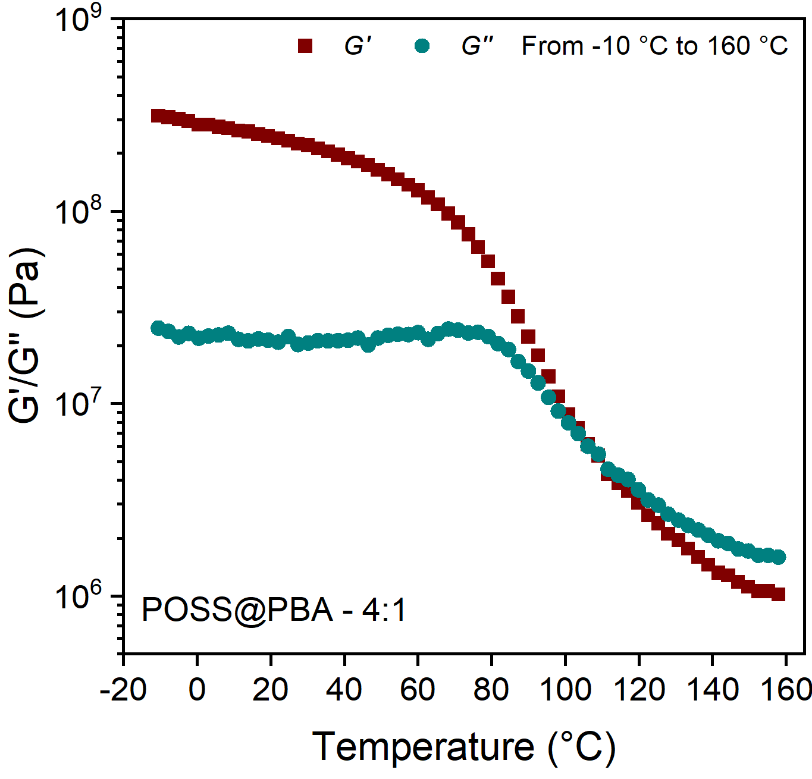


**Figure S21** Representative temperature sweep data of POSS@PBA - 4:1.


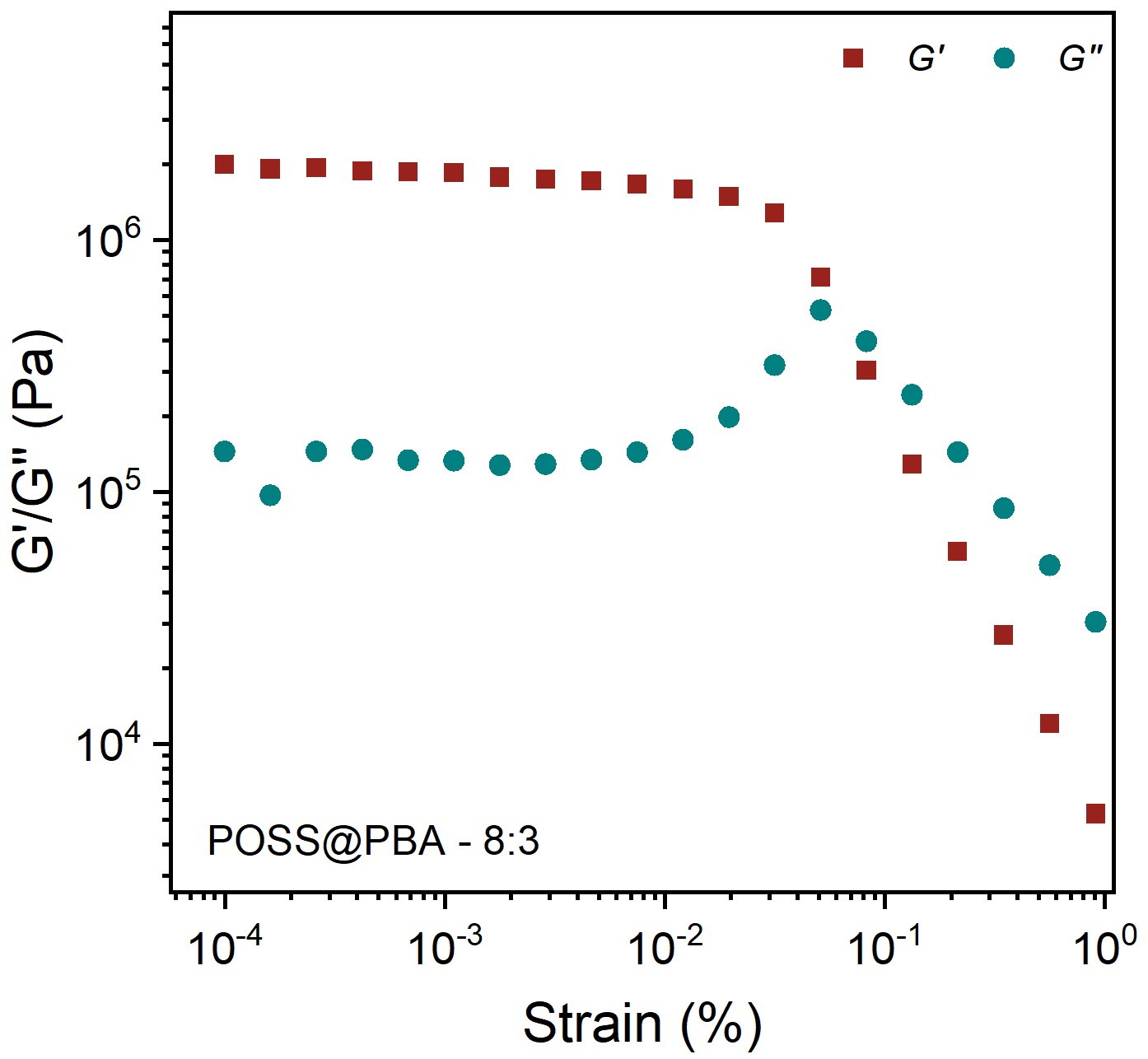


**Figure S22** Amplitude sweep (25 ^o^C, *ω* = 5 rad s^-1^) of POSS@PBA - 8:3.


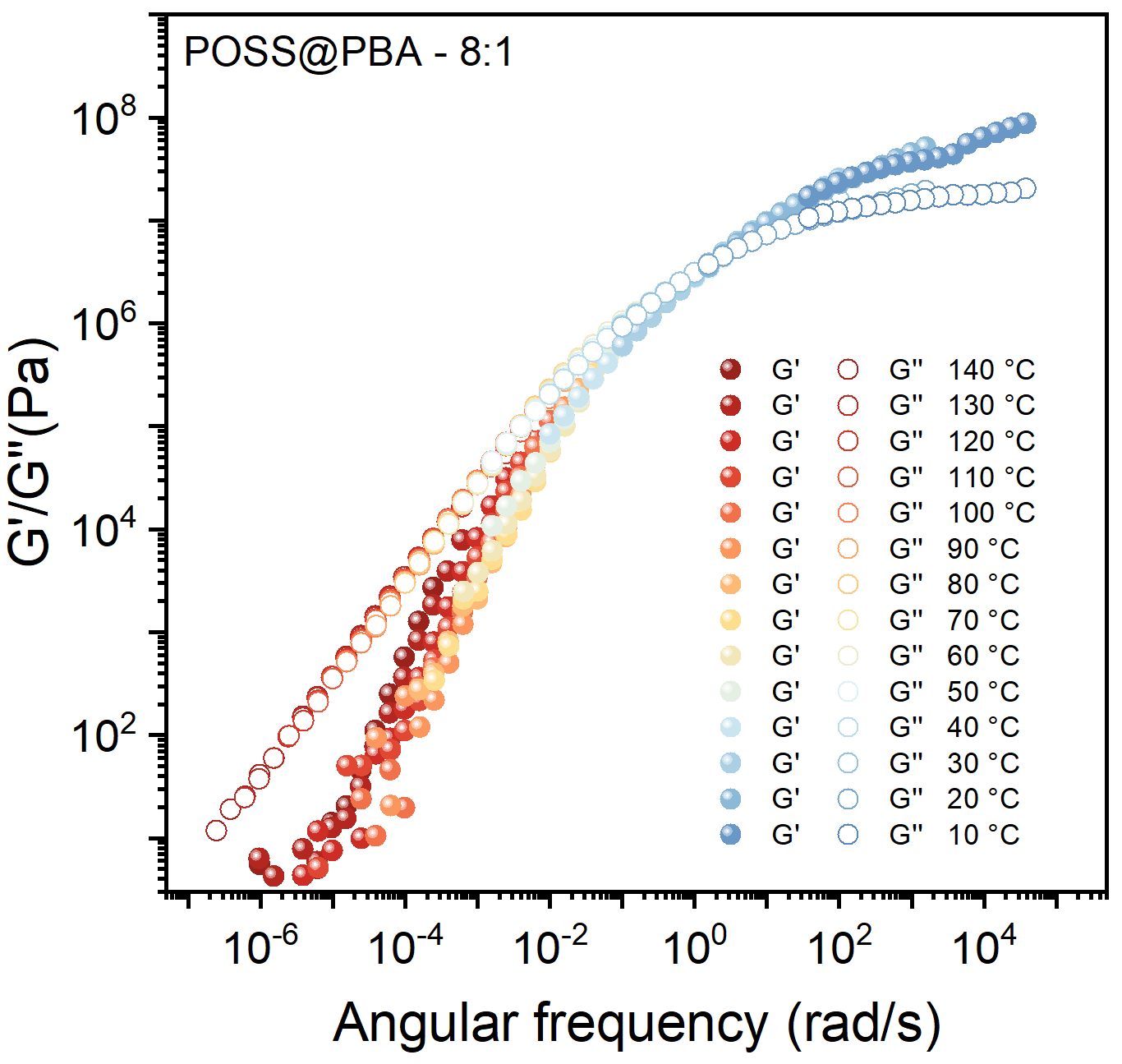


**Figure S23** Master curves of POSS@PBA - 8:1 at *T*_f_ of 30 °C from SAOS experiment.


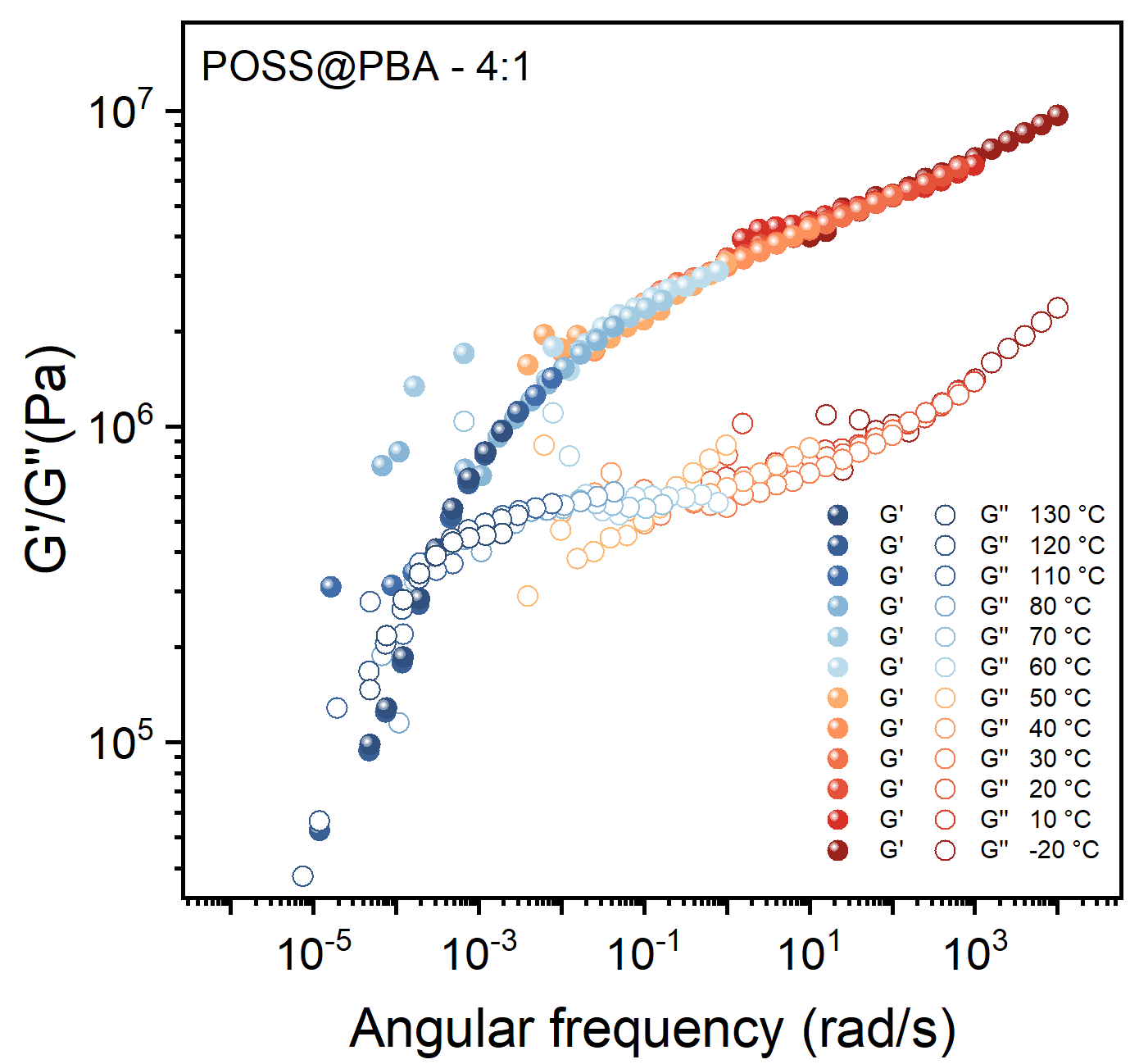


**Figure S24** Master curves of POSS@PBA - 4:1 at *T*_f_ of 30 °C from SAOS experiment.


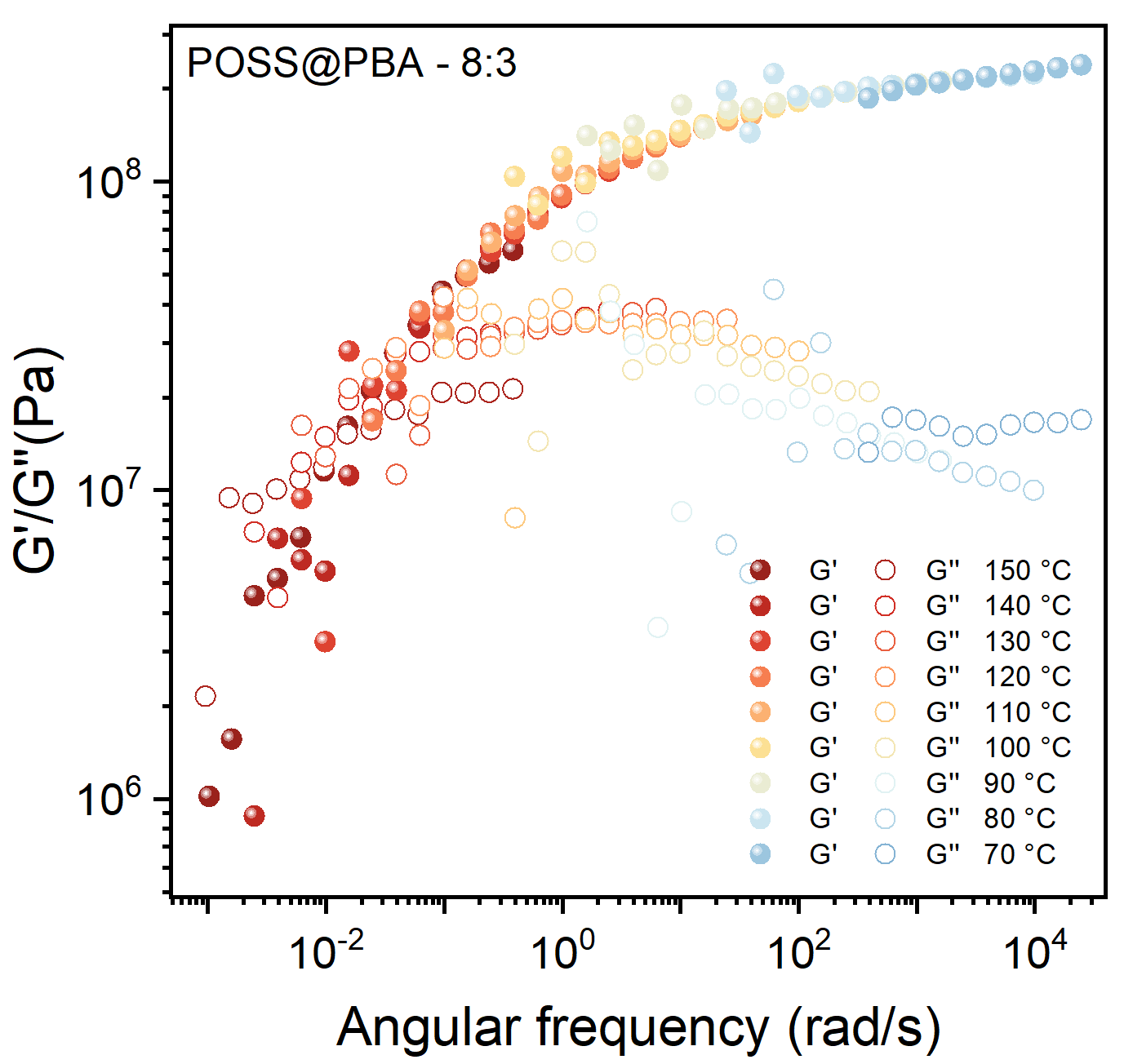


**Figure S25** Master curves of POSS@PBA - 8:3 at *T*_f_ of 30 °C from SAOS experiment.


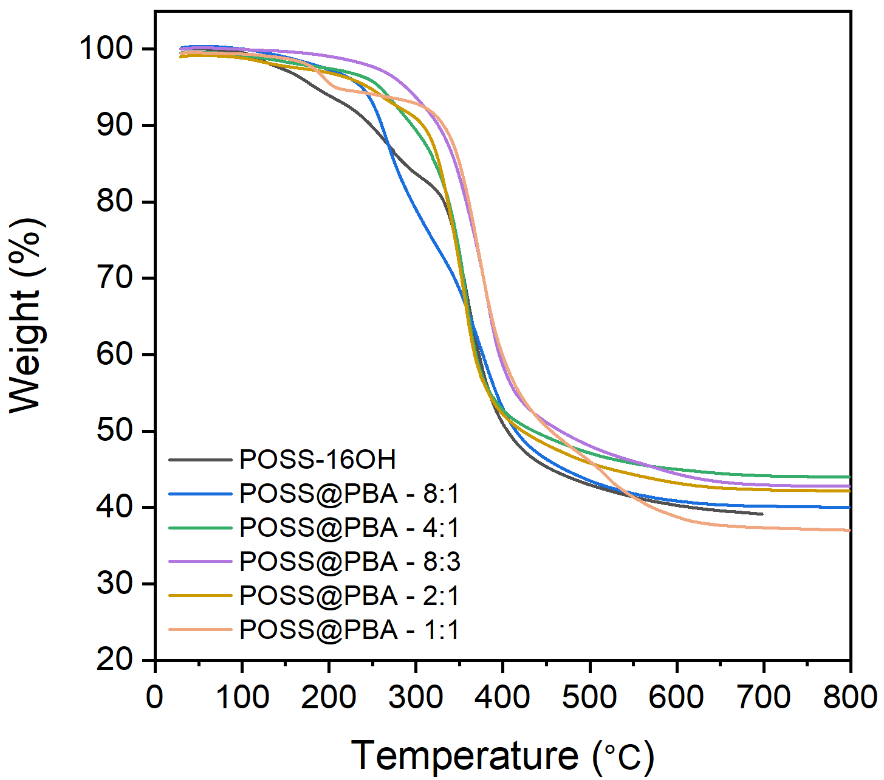


**Figure S26** TGA curves of the POSS@PBA at temperatures from 30 to 800 ℃.


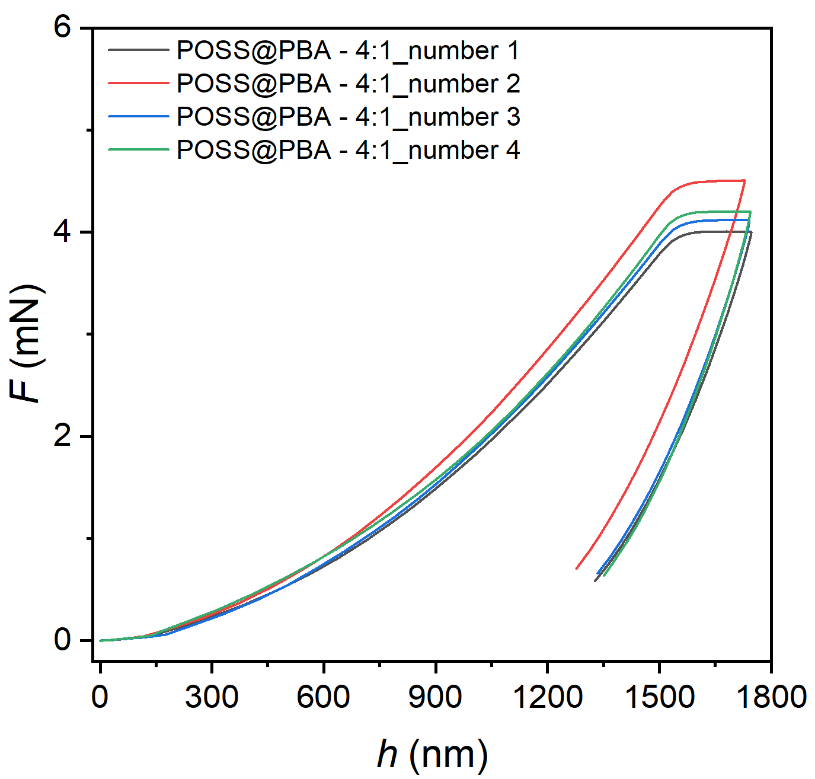


**Figure S27** Force-displacement curves of the nanoindentation POSS@PBA - 4:1.


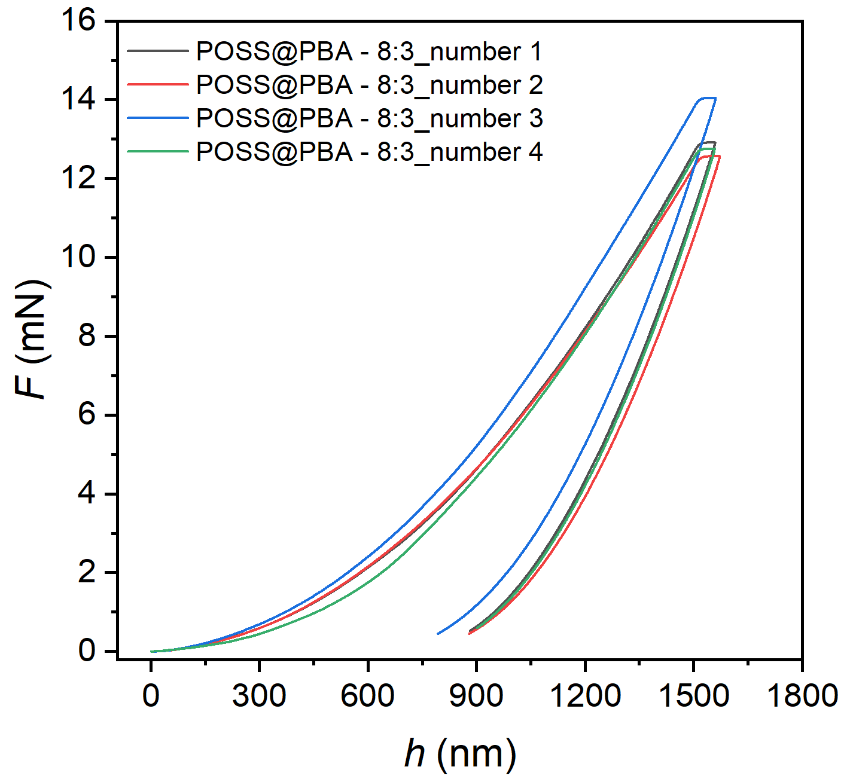


**Figure S28** Force-displacement curves of the nanoindentation POSS@PBA - 8:3.


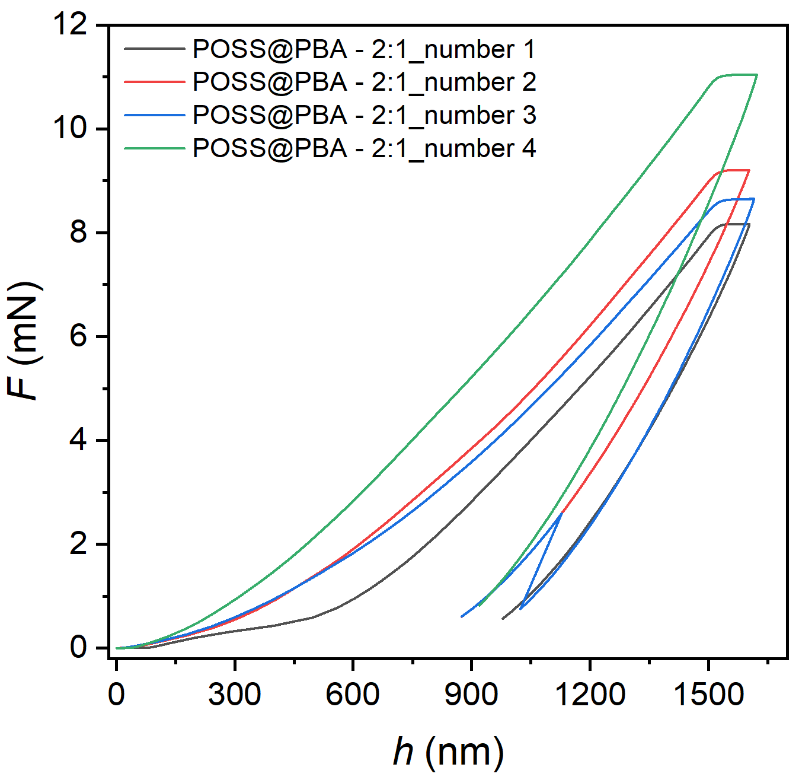


**Figure S29** Force-displacement curves of the nanoindentation POSS@PBA - 2:1.


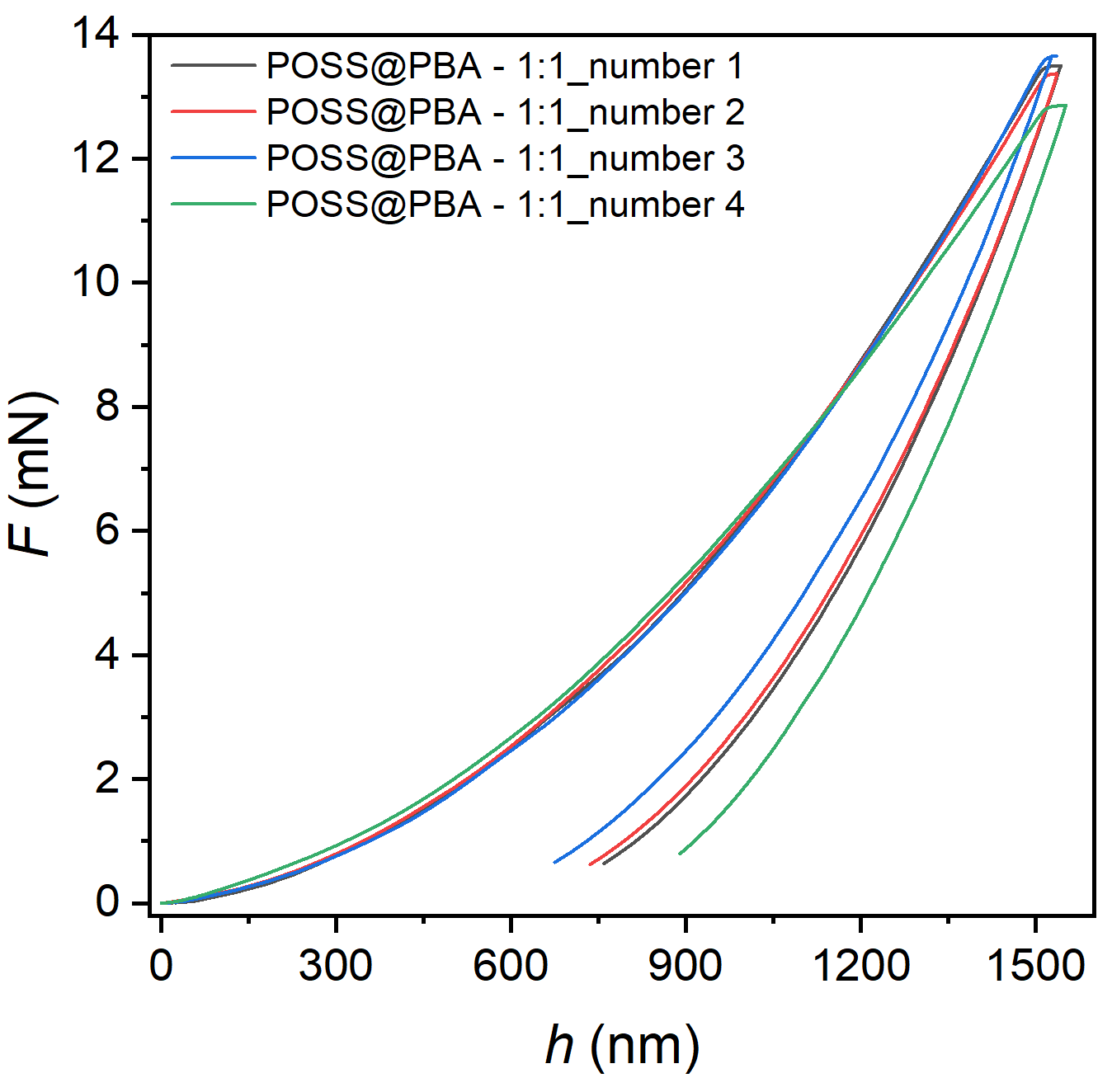


**Figure S30** Force-displacement curves of the nanoindentation POSS@PBA - 1:1.

**
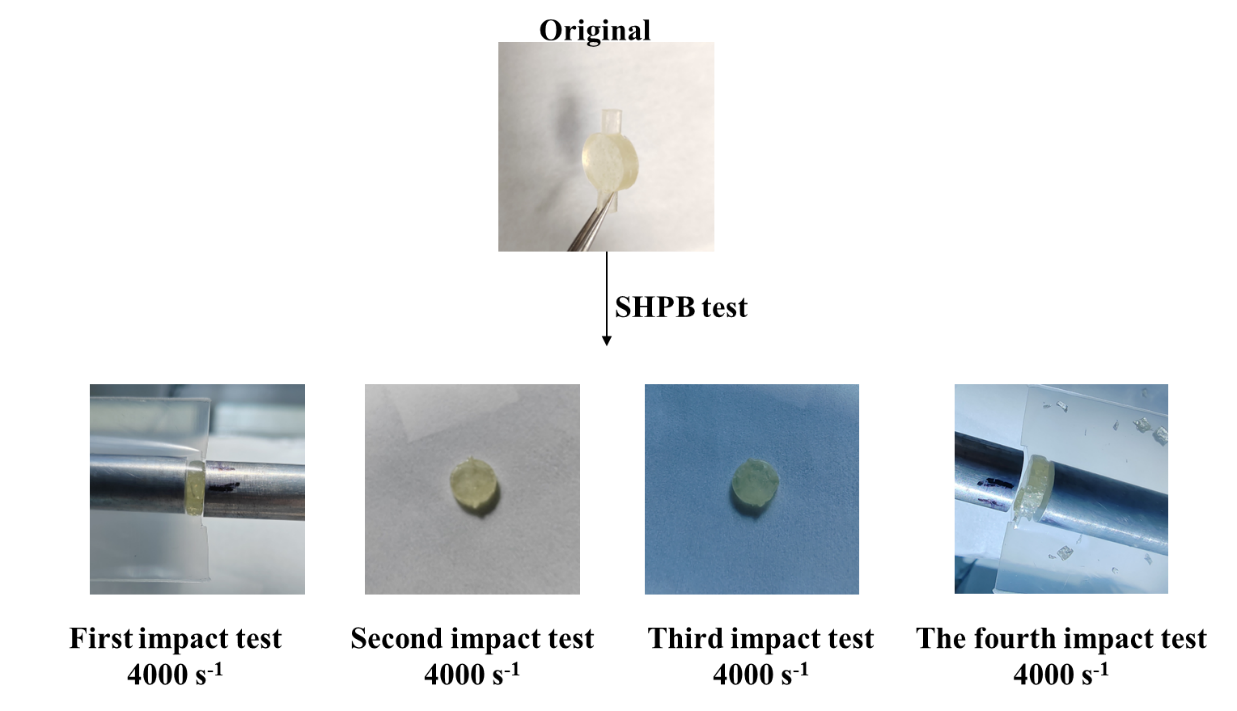
**

**Figure S31** Digital photographs for the specimens and the recycle and re-impact test for 4 times at 4000 s^-1^.


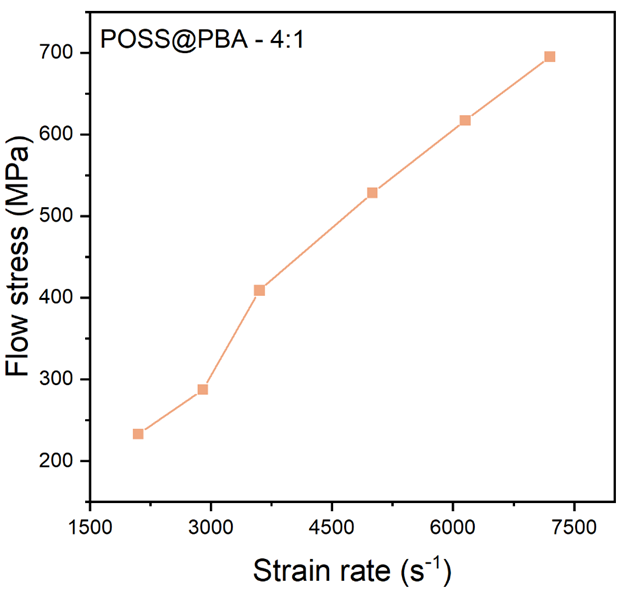


**Figure S32** Flow stress at different strain rates of POSS@PBA - 4:1 from SHPB tests.


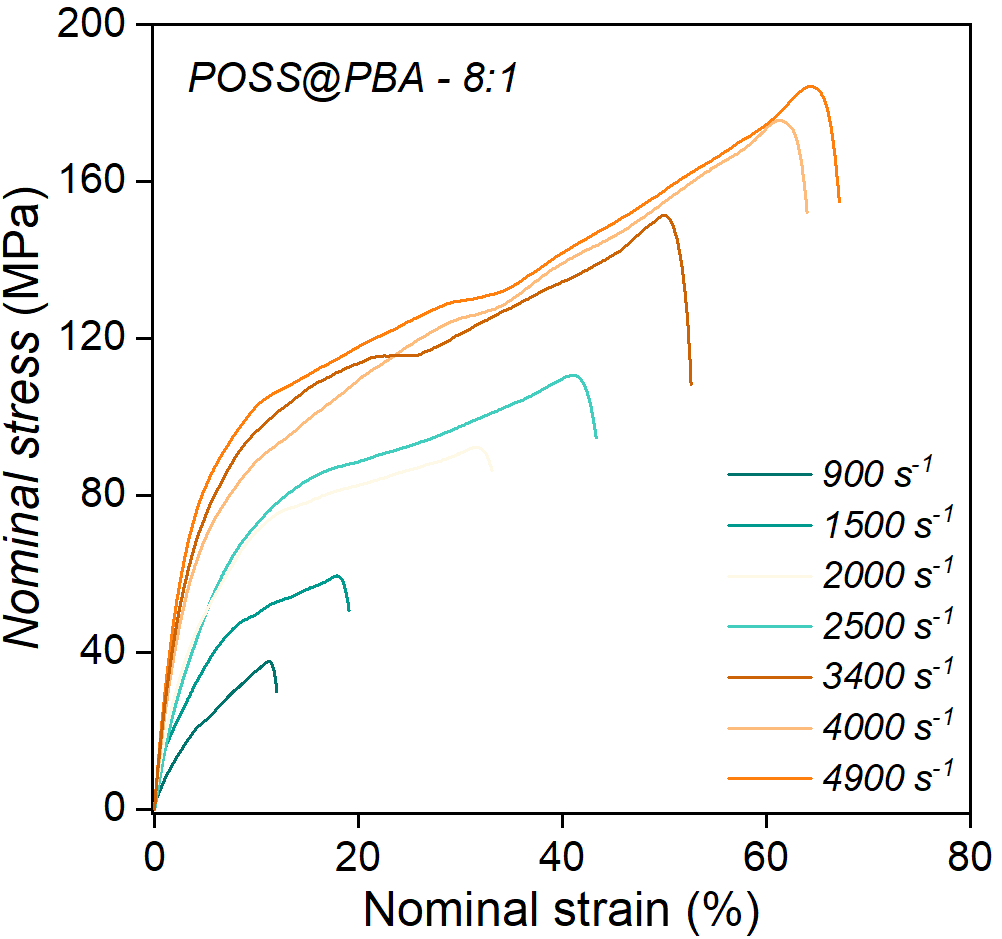


**Figure S33** Compressive stress-strain curves at different strain rates of POSS@PBA - 8:1 from SHPB tests.


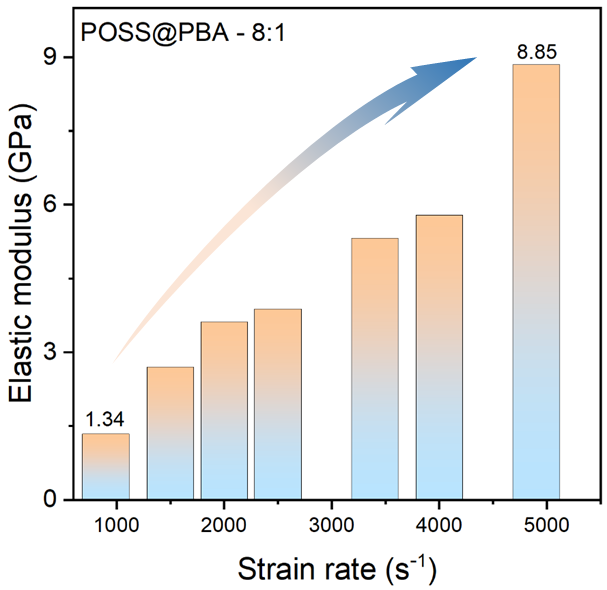


**Figure S34** Compressive strength at different strain rates of POSS@PBA - 8:1 from SHPB tests.


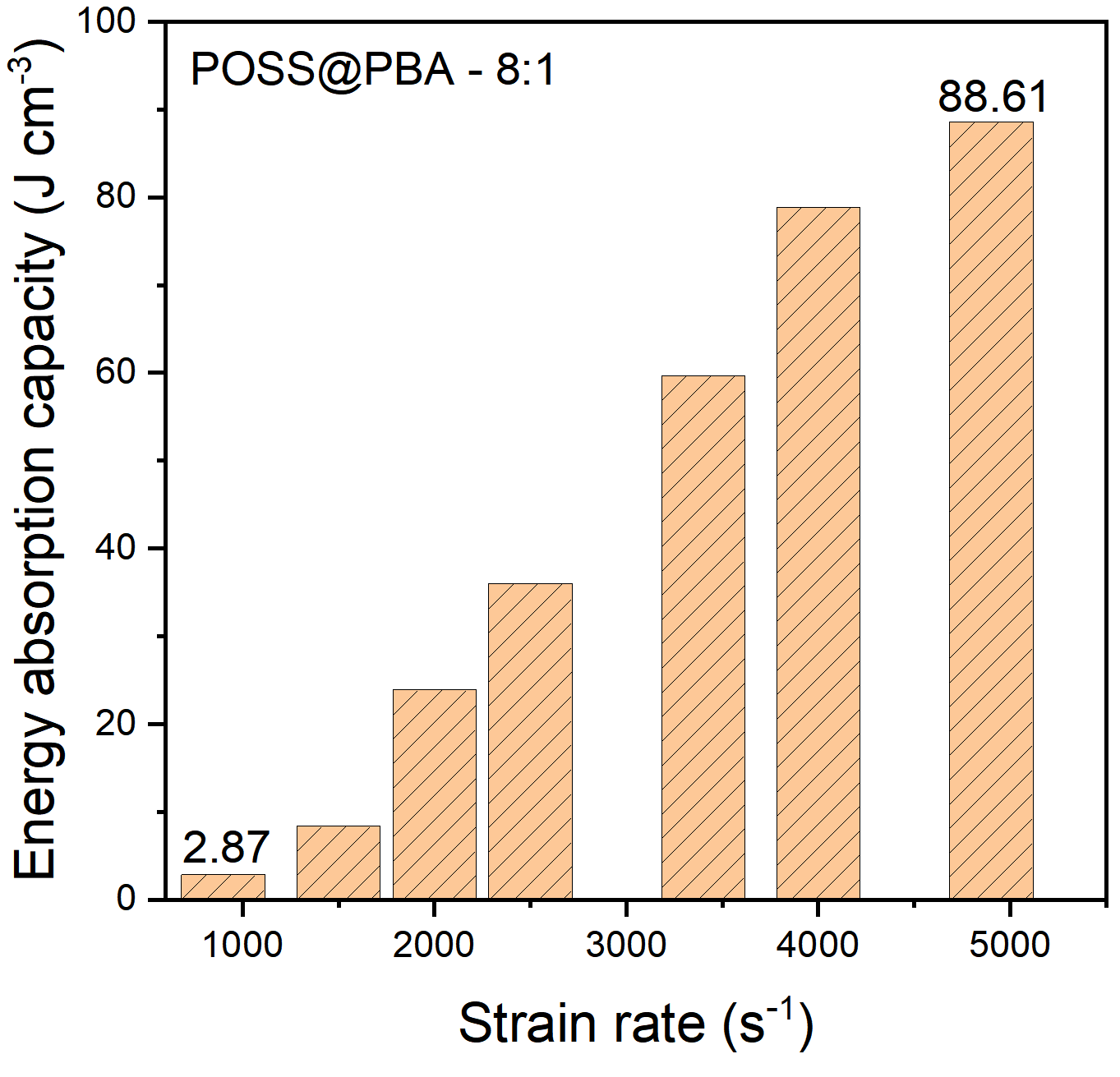


**Figure S35** Energy absorption capacities at different strain rates of POSS@PBA - 8:1 from SHPB tests.


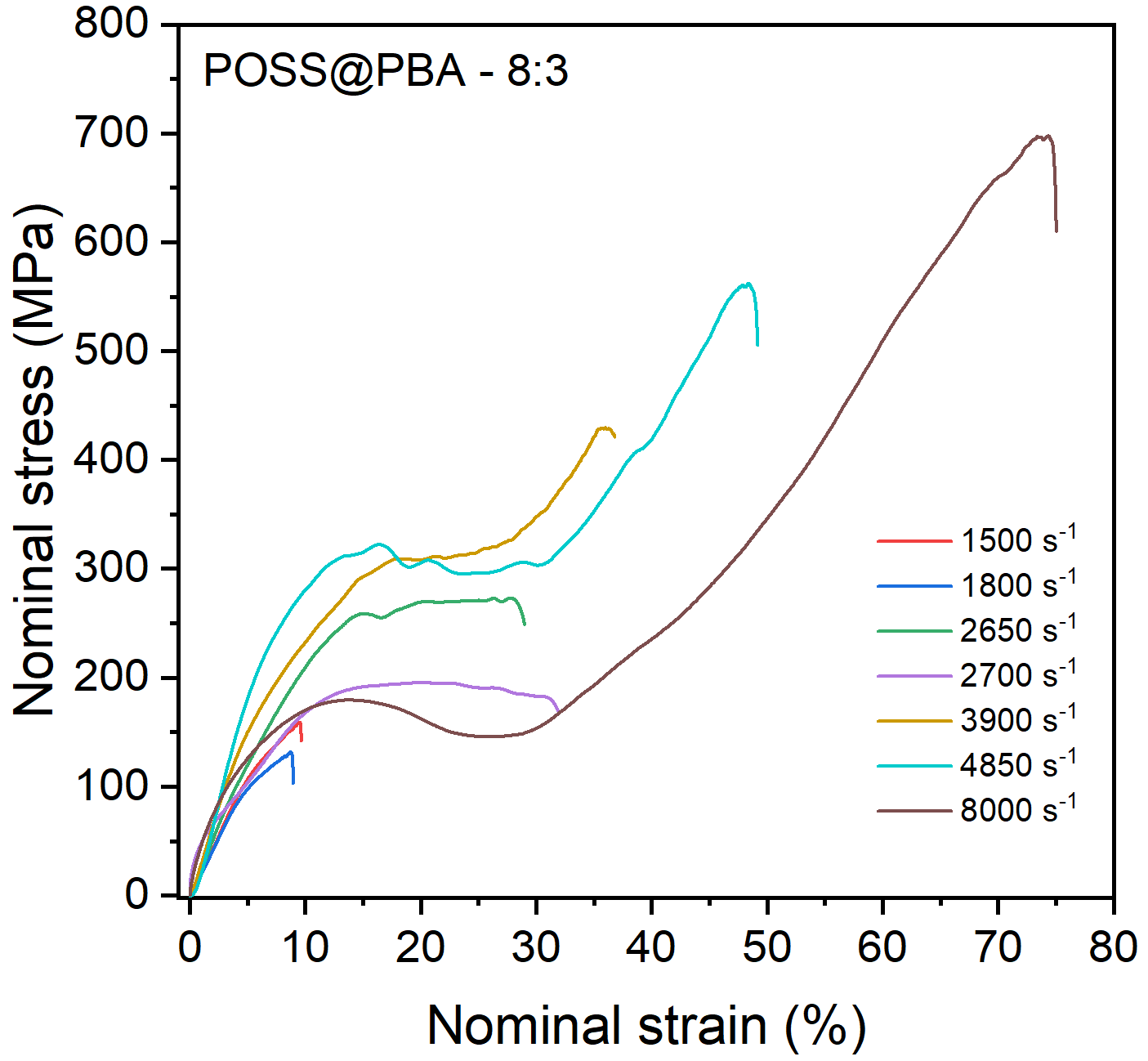


**Figure S36** Compressive stress-strain curves at different strain rates of POSS@PBA - 8:3 from SHPB tests.


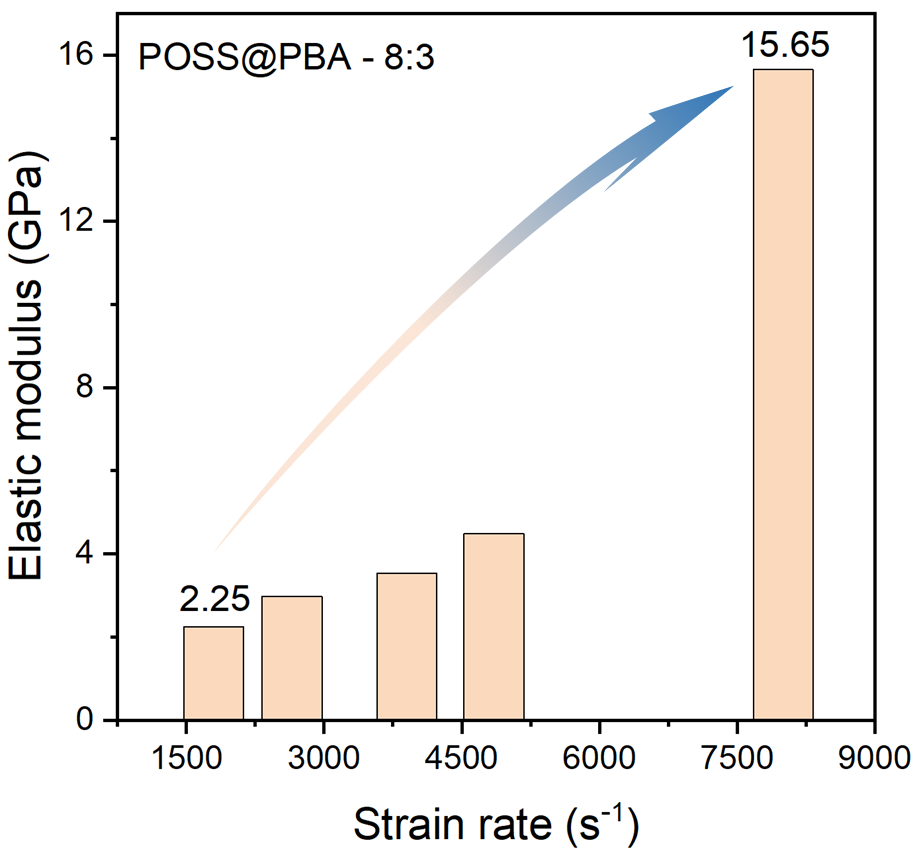


**Figure S37** Compressive strength at different strain rates of POSS@PBA - 8:3 from SHPB tests.


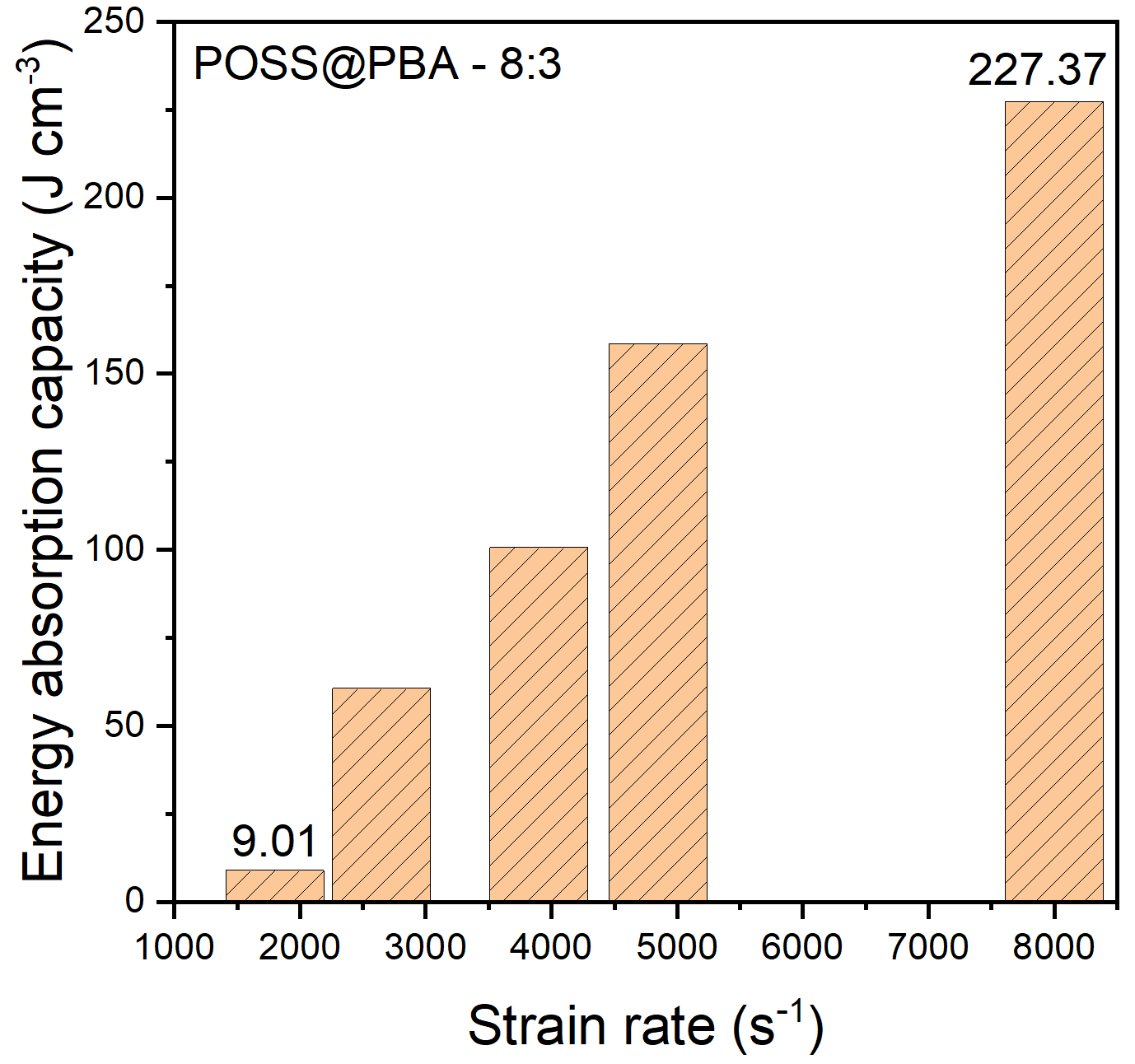


**Figure S38** Energy absorption capacities at different strain rates of POSS@PBA - 8:3 from SHPB tests.


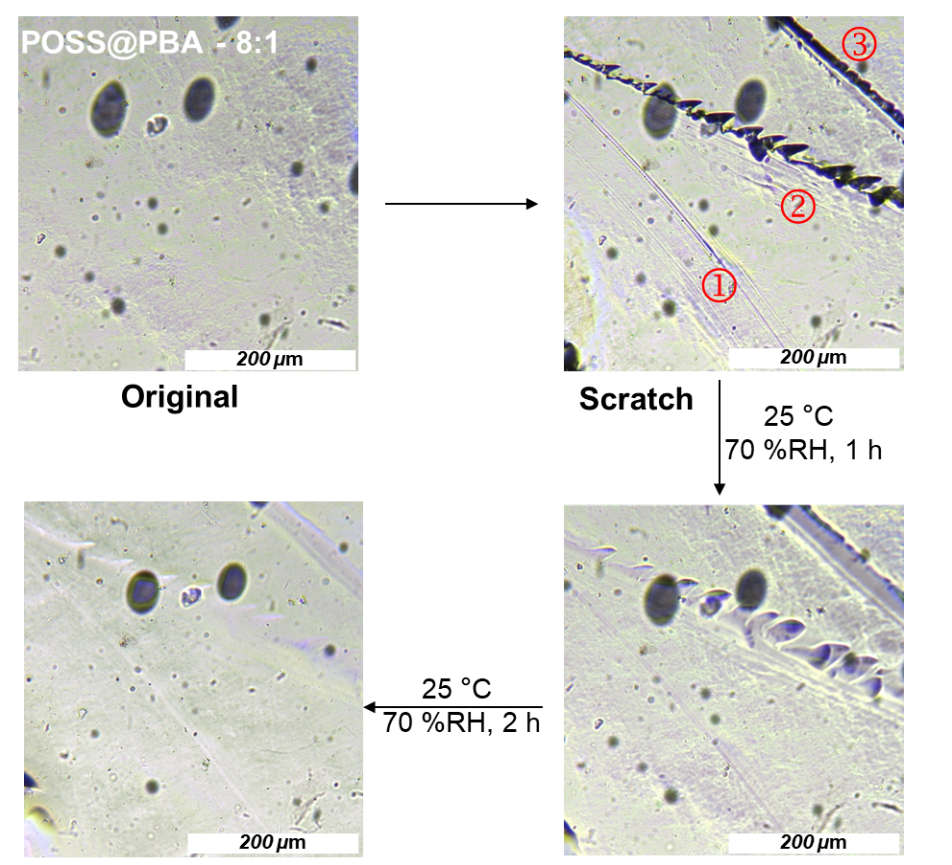


**Figure S39** Photographs of the scratch and crack healing process of POSS@PBA - 8:1.


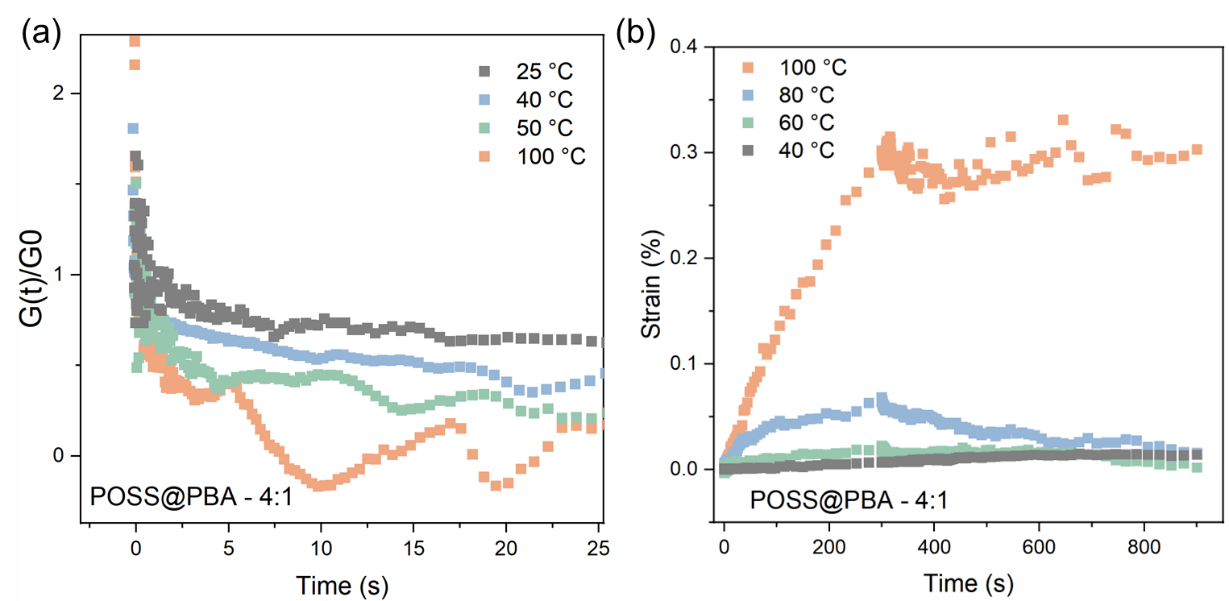


**Figure S40** (a) Normalized stress relaxation curves of POSS@PBA - 4:1. (b) Creep curves of POSS@PBA - 4:1.


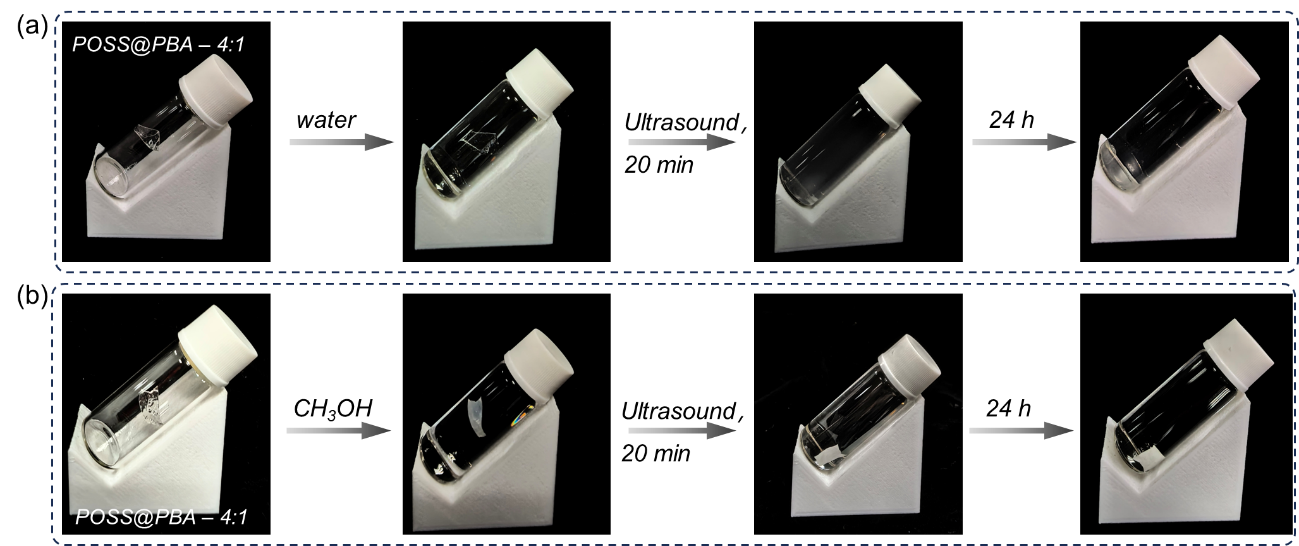


**Figure S41** POSS@PBA - 4:1 film was respectively immersed in water (a) and methanol (b) in glass vials at ambient temperature. Due to the susceptibility of the boronic ester bonds to hydrolysis upon contact with water molecules, the network undergoes structural disassembly, resulting in weakened connectivity and eventual complete dissolution of POSS@PBA - 4:1 in the aqueous solution.

**
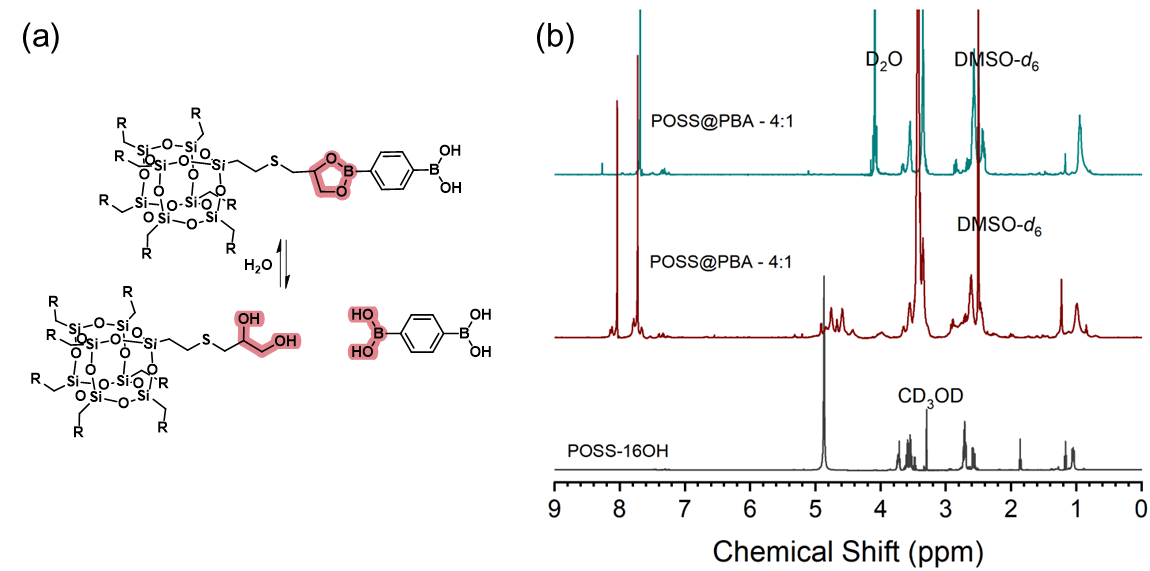
**

**Figure S42** Hydrolytic stability for POSS@PBA - 4:1. (a)Hydrolysis reaction of POSS@PBA. (b) ^1^H NMR spectra of POSS@PBA in DMSO-*d*_6_ and DMSO-*d*_6_ /D_2_O at 298 k, respectively. In the DMSO-*d*_6_ /D_2_O solvent system, all of the POSS@PBA were completely hydrolyzed to their corresponding boronic acids and triol components. The disappearance of the characteristic chemical shift corresponding to the five-membered ring of the boronic ester bond clearly confirms the hydrolytic reversibility of the system and unambiguously verifies the presence of dynamic boronic ester linkages in the materials.


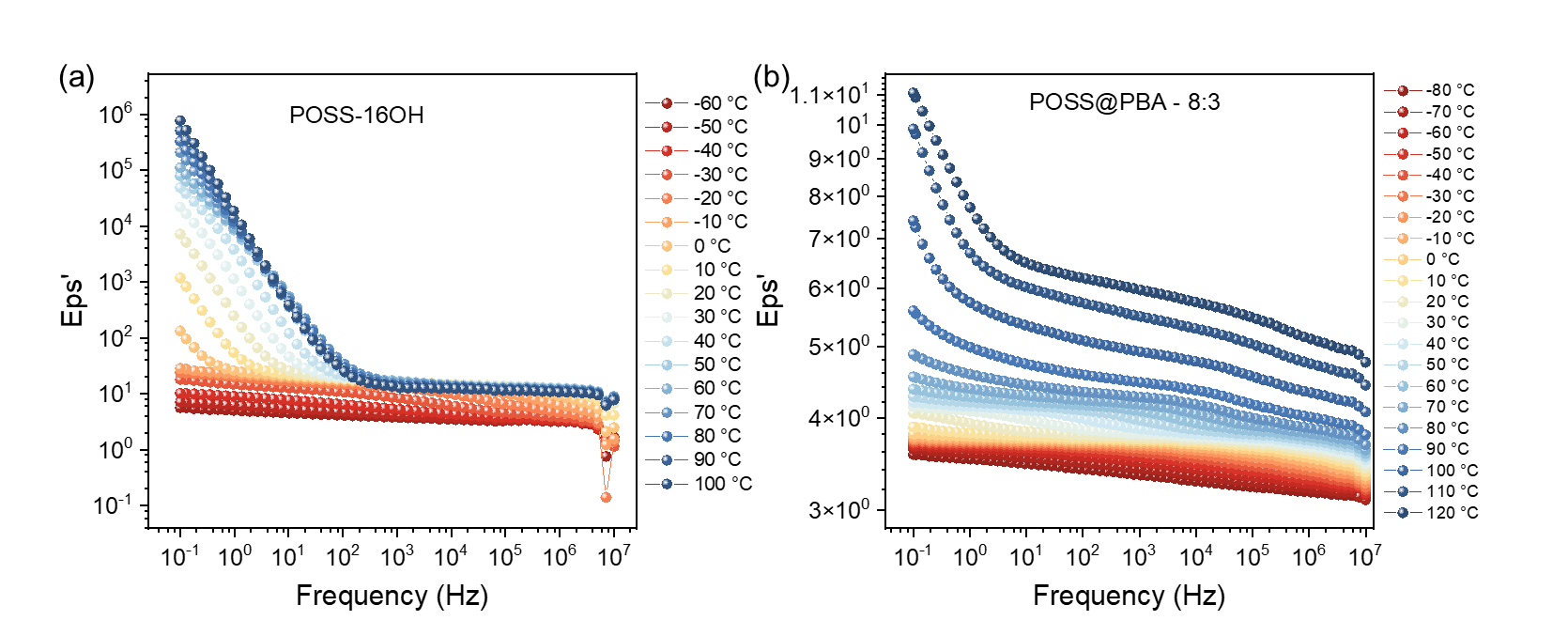


**Figure** **S43** The real part of complex dielectric function (ε′) over frequency of (a) POSS-16OH and (b) POSS@PBA - 8:3.


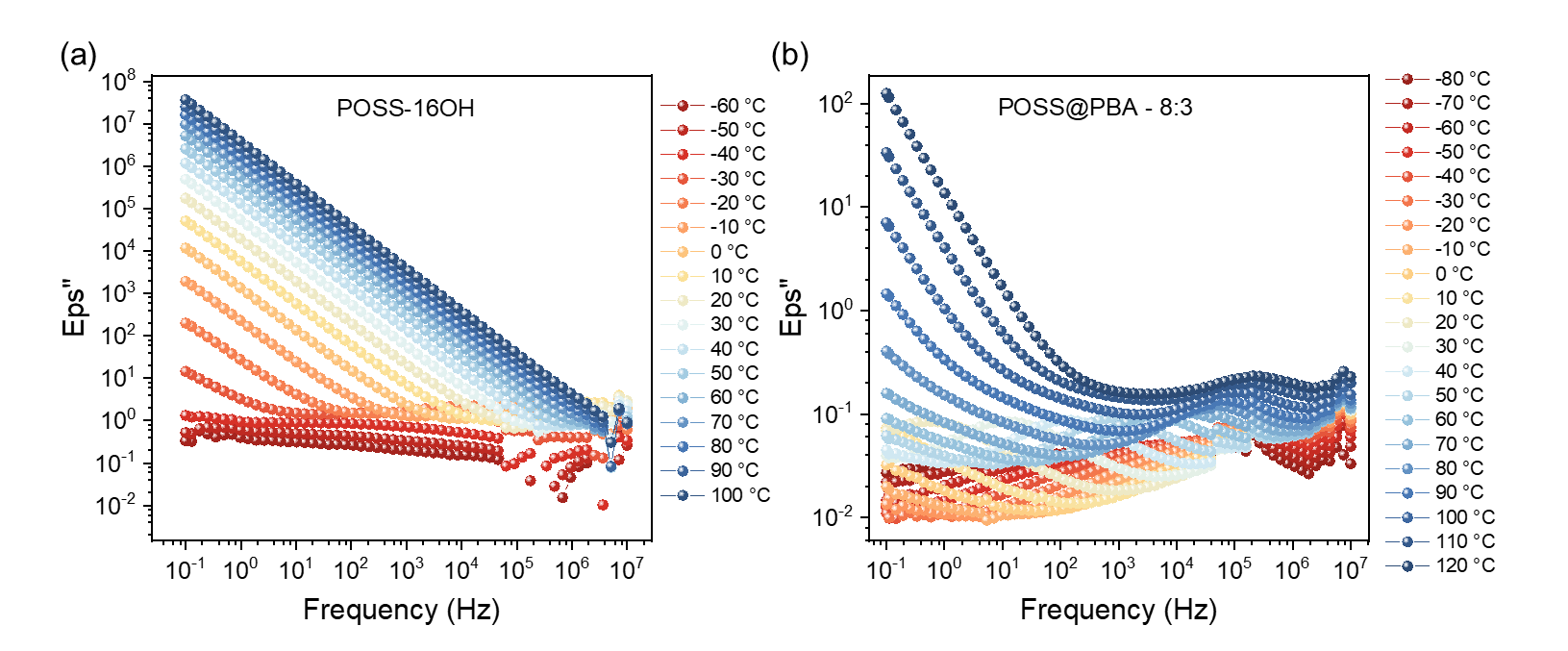


**Figure** **S44** The imaginary part of complex dielectric function (ε′′) of (a) POSS-16OH and (b) POSS@PBA - 8:3.


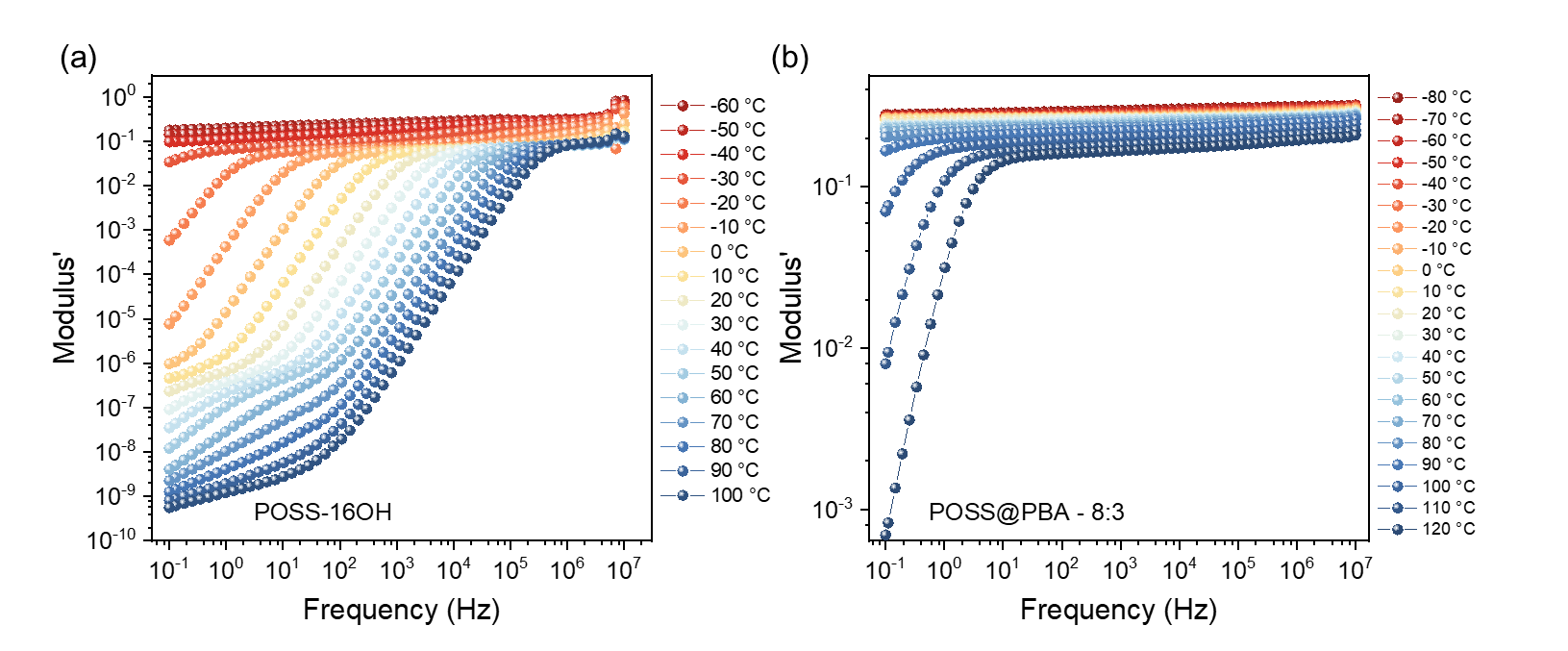


**Figure S45** The real part of complex modulus (M′) of (a) POSS-16OH and (b) POSS@PBA - 8:3.


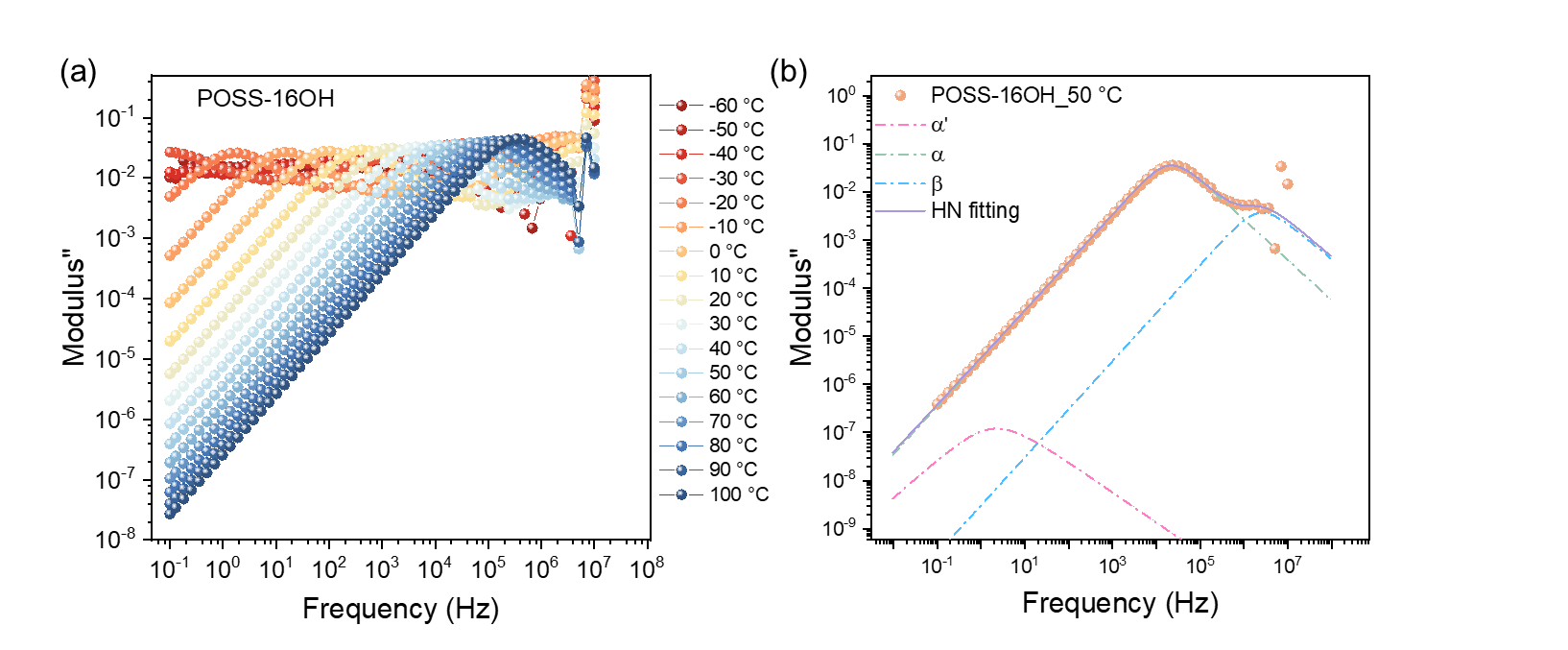


**Figure S46** (a) The imaginary part of complex modulus for POSS-16OH from -60 ~ 100 ℃. (b) The imaginary part of complex modulus for POSS-16OH at 50 ℃ and the fitting curve by using a sum of three HN functions in the modulus expression.


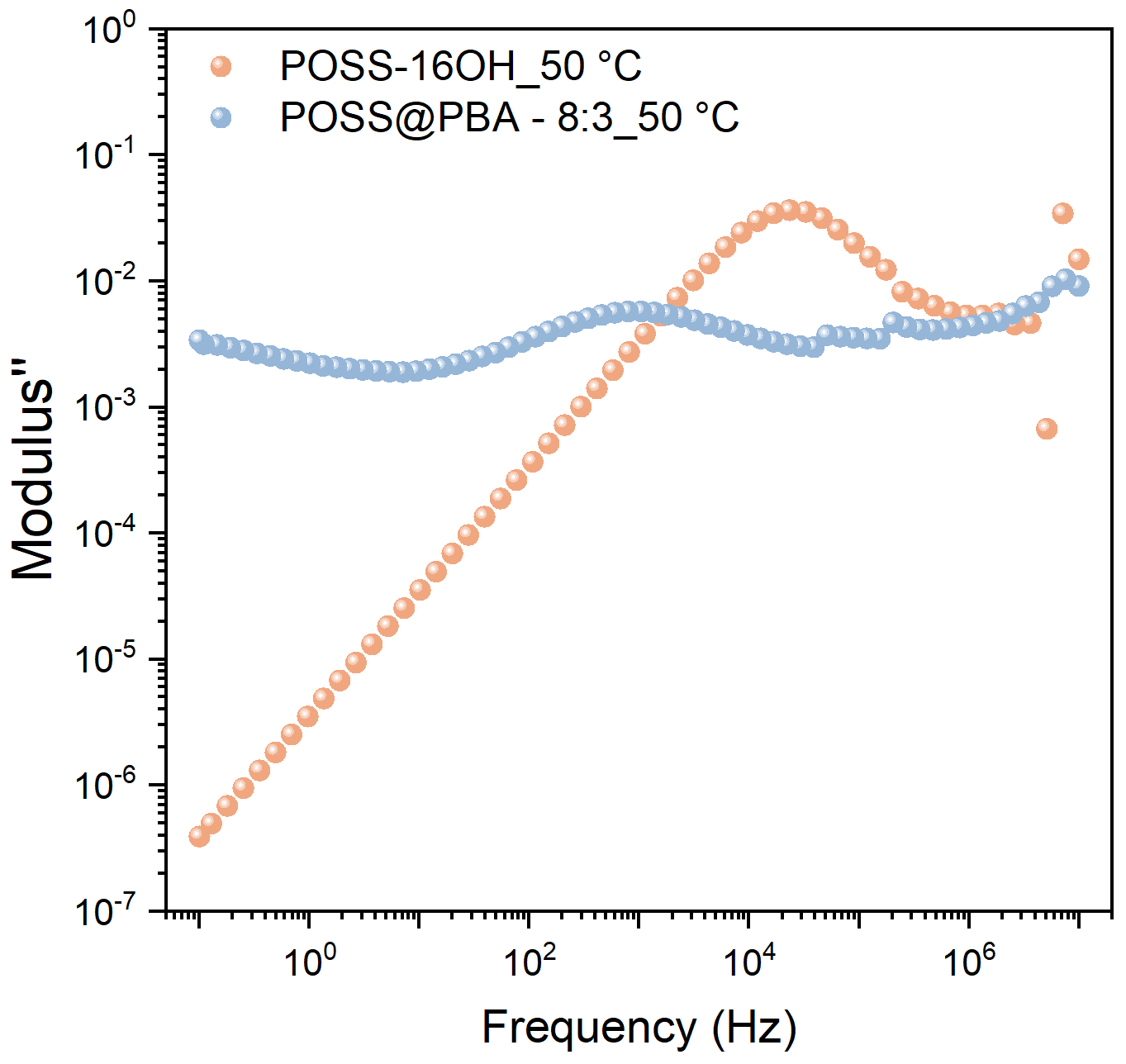


**Figure S47** The comparison of the imaginary part of complex modulus (M′′) of POSS-16OH and POSS@PBA - 8:3 at 50 ℃.


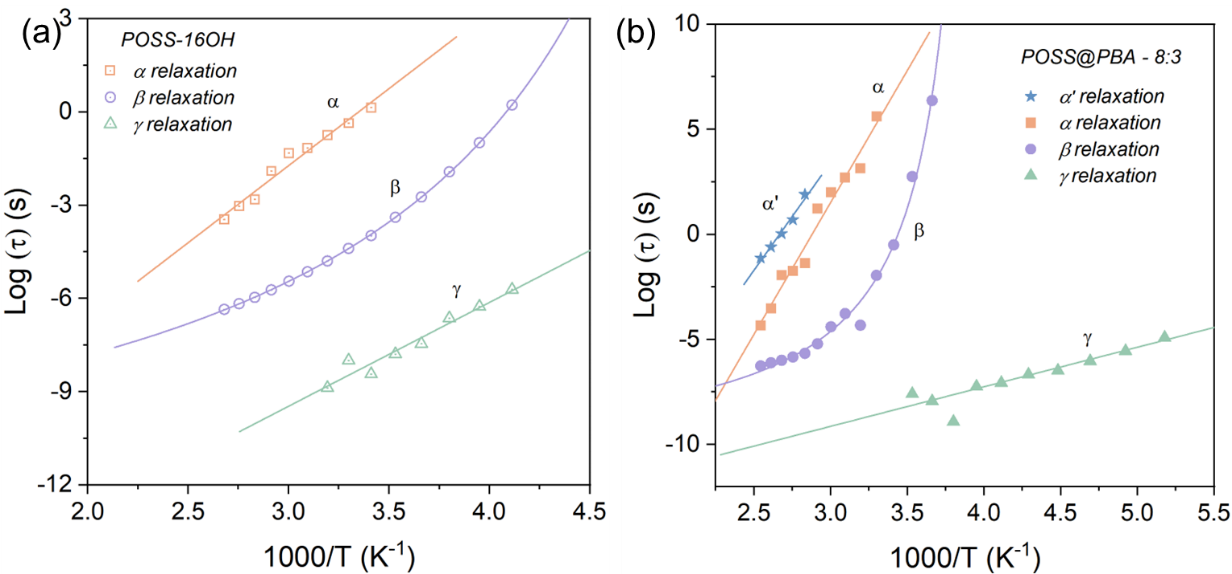


**Figure S48** The temperature dependence of relaxation time (τ) obtained by HN functions fitting. (a) POSS-16OH. (b) POSS@PBA - 8:3.


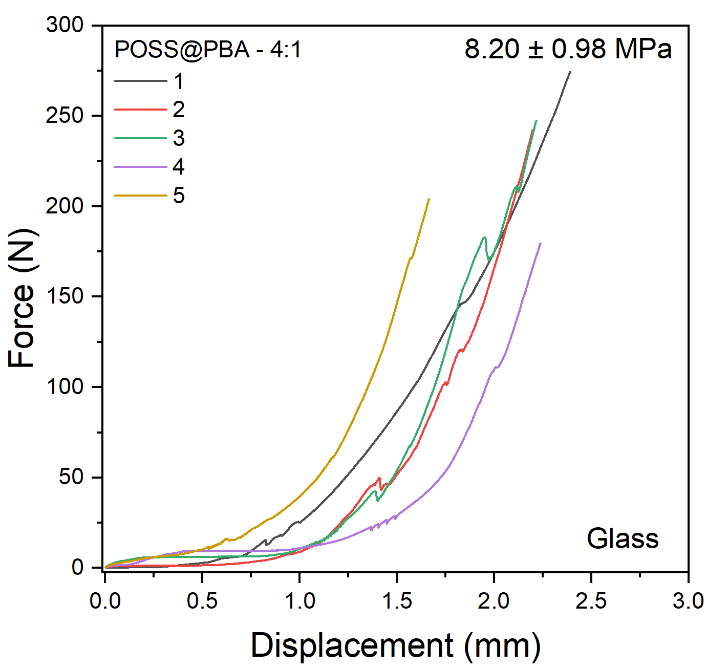


**Figure S49** Peeling strength of POSS@PBA - 4:1 on the surface of glass.


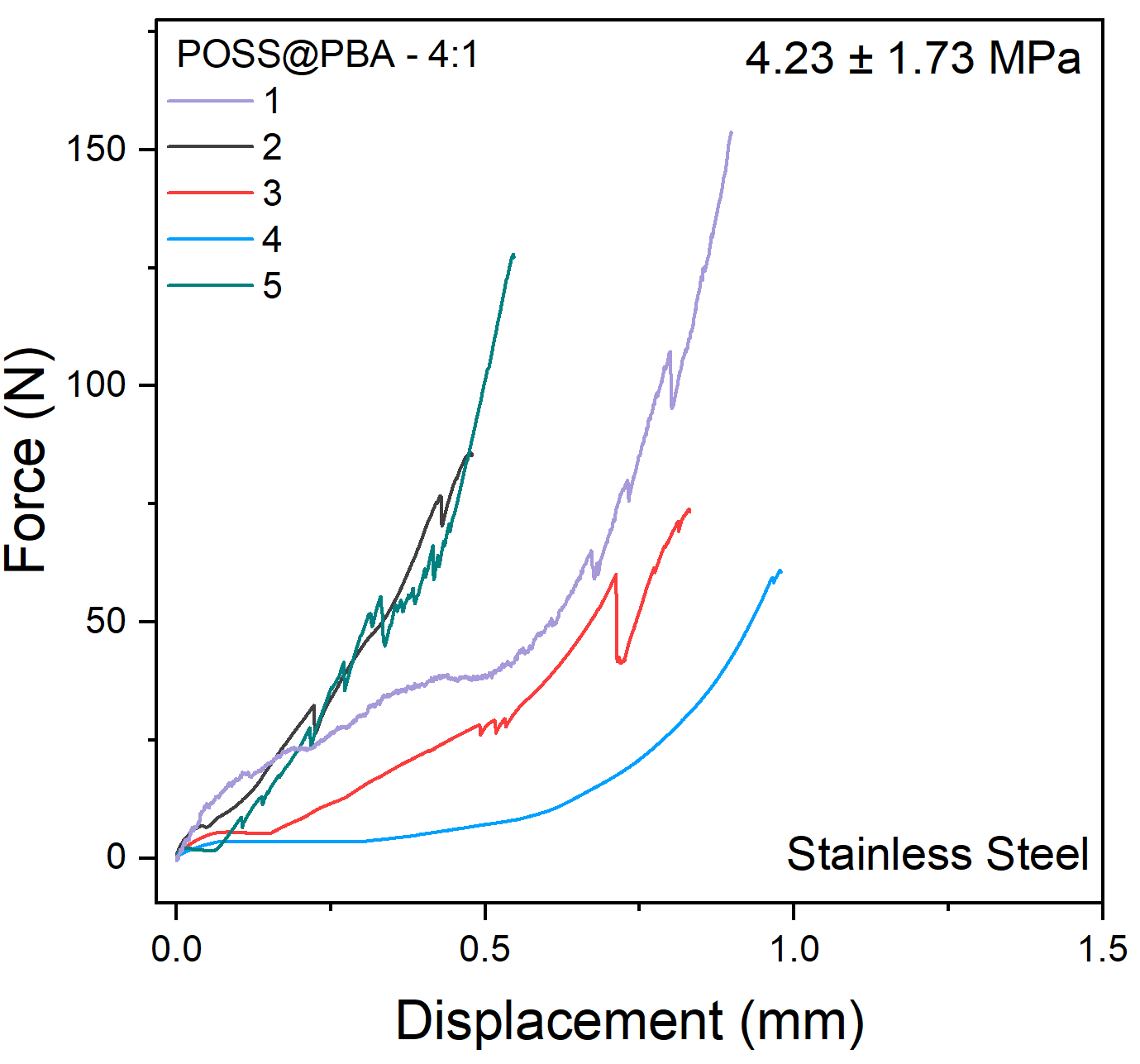


**Figure S50** Peeling strength of POSS@PBA - 4:1 on the surface of stainless steel.


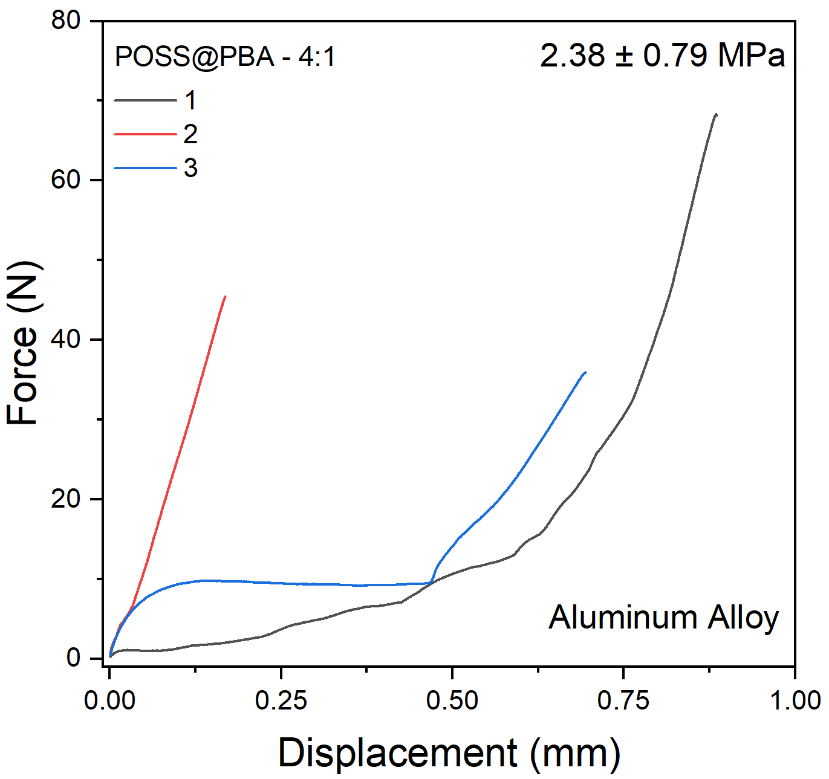


**Figure S51** Peeling strength of POSS@PBA - 4:1 on the surface of aluminum alloy.

**
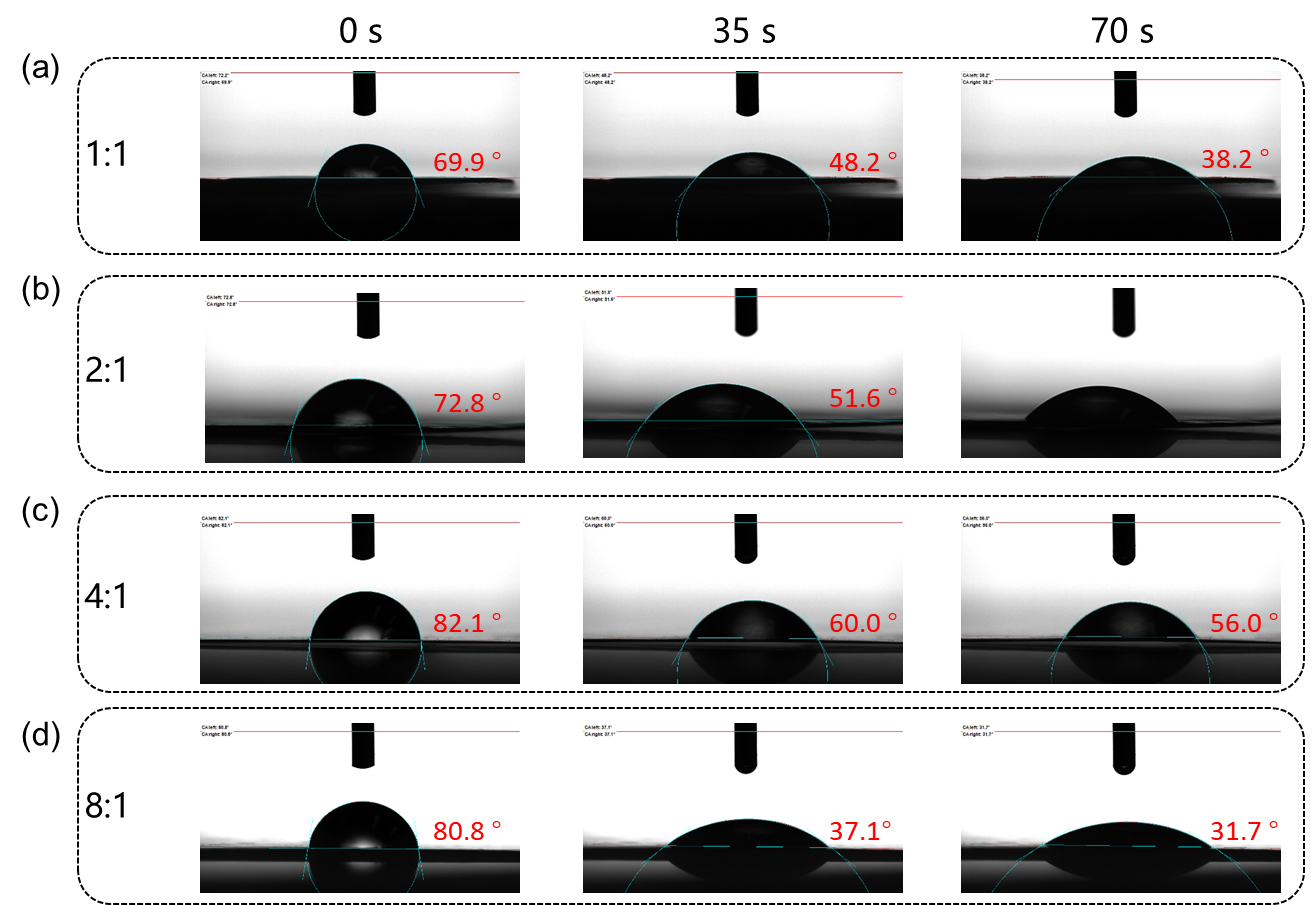
**

**Figure S52** Water contact angles as a function of time for different mass ratio of POSS@PBA. (a) 1:1. (b) 2:1. (c)4:1. (d) 8:1.

**4.2 Supplementary Tables**

**Table S1** The corresponding molar ratios and functional group ratios of different POSS@PBA samples

| samples | POSS-16OH:PBA | | |
| --- | --- | --- | --- |
|  | mass ratios | molar ratios | functional group ratios |
| POSS@PBA - 8:1 | 8:1 | 1:1 | 4:1 |
| POSS@PBA - 4:1 | 4:1 | 1:2 | 2:1 |
| POSS@PBA - 8:3 | 8:3 | 1:3 | 4:3 |
| POSS@PBA - 2:1 | 2:1 | 1:4 | 1:1 |
| POSS@PBA - 1:1 | 1:1 | 1:8 | 1:2 |

**Table S2** Summary of POSS@PBA - 8:1 mechanical properties

| POSS@PBA - 8:1 | Test number | | | |
| --- | --- | --- | --- | --- |
|  | 1 | 2 | 3 | 4 |
| Stretching rate (mm/min) | 10 | 10 | 10 | 10 |
| Young’s modulus (MPa) | 3.47 | 2.64 | 3.59 | 4.22 |
| Elongation (%) | 574.21 | 521.27 | 284.73 | 226.84 |
| Strength (MPa) | 0.008 | 0.010 | 0.007 | 0.011 |

**Table S3** Summary of POSS@PBA - 4:1 mechanical properties

| POSS@PBA - 4:1 | 1 | 2 | 3 | 4 | 5 |
| --- | --- | --- | --- | --- | --- |
| Stretching rate (mm/min) | 10 | 10 | 10 | 10 | 10 |
| Young’s modulus (MPa) | 920.99 | 1061.44 | 1048.19 | 974.74 | 1208.05 |
| Elongation (%) | - | - | 1.72 | 1.11 | 1.03 |
| Strength (MPa) | 9.58 | 13.37 | 13.94 | 9.21 | 10.68 |

**Table S4** Summary of POSS@PBA - 8:3 mechanical properties

| POSS@PBA - 8:3 | 1 | 2 | 3 | 4 | 5 | 6 |
| --- | --- | --- | --- | --- | --- | --- |
| Stretching rate (mm/min) | 10 | 10 | 10 | 10 | 10 | 10 |
| Young’s modulus (MPa) | 2552.6 | 2084.17 | 1738.43 | 1617.41 | 1453.69 | 1389.16 |
| Elongation (%) | 0.15 | 0.56 | 0.77 | 0.23 | 0.16 | 0.69 |
| Strength (MPa) | 3.56 | 9.86 | 11.77 | 3.23 | 2.17 | 6.53 |

**Table S5** Summary of POSS@PBA mechanical properties

| POSS@PBA | Mass ratio | | |
| --- | --- | --- | --- |
|  | 8:1 | 4:1 | 8:3 |
| Stretching rate (mm/min) | 10 | 10 | 10 |
| Young’s modulus (MPa) | 3.51±0.61 | 1036.53±100.30 | 1785.81±427.52 |
| Elongation (%) | 400.65±173.09 | 1.29±0.38 | 0.56±0.21 |

**Table S6** Summary of POSS@PBA - 4:1 mechanical properties from nanoindentation test

| POSS@PBA - 4:1 | 1 | 2 | 3 | 4 |
| --- | --- | --- | --- | --- |
| Holding time (s) | 5 | 10 | 15 | 20 |
| H (MPa) | 87.367 | 123.88 | 113.8 | 107.35 |
| E (GPa) | 1.30 | 1.33 | 1.77 | 1.86 |
| E′ (GPa) | 1.39 | 1.42 | 1.89 | 1.98 |

**Table S7** Summary of POSS@PBA - 8:3 mechanical properties from nanoindentation test

| POSS@PBA - 8:3 | 1 | 2 | 3 | 4 |
| --- | --- | --- | --- | --- |
| Holding time (s) | 5 | 5 | 5 | 5 |
| H (MPa) | 351.32 | 331.36 | 397.78 | 344.27 |
| E (GPa) | 3.92 | 3.82 | 4.02 | 3.93 |
| E′ (GPa) | 4.18 | 4.08 | 4.29 | 4.19 |

**Table S8** Summary of POSS@PBA mechanical properties from nanoindentation test

| POSS@PBA | Mass ratio | | | |  |
| --- | --- | --- | --- | --- | --- |
|  | 1:1 | 2:1 | 8:3 | 4:1 | |
| H (GPa) | 0.39 ± 0.03 | 0.25 ± 0.04 | 0.36 ± 0.03 | 0.08 ± 0.01 | |
| HV | 39.80 ± 3.06 | 25.51 ± 4.08 | 36.73 ± 3.06 | 8.16 ± 1.02 | |
| Tg (℃) | 82 | 79 | 80 | 57 | |
| E (GPa) | 4.13 ± 0.05 | 2.64 ± 0.21 | 3.92 ± 0.08 | 1.48 ± 0.10 | |

**Table S9** Comparison of mechanical properties between POSS@PBA and traditional polymers

| Materials | Young's modulus (GPa) | Materials | Young's modulus (GPa) |
| --- | --- | --- | --- |
| LDPE | 0.11-0.45 | PP | 1.5-2 |
| HDPE | 0.8 | PVC | 2-4 |
| PA | 1-4 | PS | 2.5-3.5 |
| Nylon1010 | 1.07 | PMMA | 2-4 |
| PTFE | 1.14-1.42 | PET | 2-2.7 |
| PBT | 1.93 | PC | 2-2.5 |
| PEEK | 3-4 | PI | 3-4 |
| POM | 2.6 | **POSS@PBA** | **1.79** |

**Table S10** Comparison of mechanical properties between POSS@PBA and polymer- free supramolecular interaction systems

| Materials | E (GPa) | Tg (℃) | Method | Ref.^[3-7]^ |
| --- | --- | --- | --- | --- |
| T_8_-UPy_8_ | 4.3 | 84.2 | QNM mappings | *Chem. Mater.*, **2024**, *36*, 575. |
| T_10_-UPy_10_ | 5.0 | 59.5 | QNM mappings |  |
| T_12_-UPy_12_ | 6.0 | 65.4 | QNM mappings |  |
| c-T_8_B_8_ | 2.35 | - | Nanoindentation | *Adv. Funct. Mater.*, **2021**, *31*, 2102074. |
| c-T_10_B_10_ | 2.97 | - | Nanoindentation |  |
| c-T_12_B_12_ | 3.34 | - | Nanoindentation |  |
| UPy-POSS | 4.5 | 78 | Nanoindentation | *Adv. Mater.*, **2018**, *30*, 1803854. |
| poly(UIO-TA) | 0.39 | **-** | Nanoindentation | *Adv. Sci.*, **2025**, e05122. |
| BCN | 2.80 | **-** | Nanoindentation | *Angew. Chem. Int. Ed.*, **2025**, *64*, e202424147. |
| LIN | 2.46 | **-** | Nanoindentation |  |
| **POSS@PBA** | **3.82** | **80** | **Nanoindentation** | **This work** |

**Table S11** Comparison of mechanical properties between POSS@PBA and polymer integrated supramolecular systems

| Materials | E (GPa) | Method | Ref.^[8-14]^ |
| --- | --- | --- | --- |
| PFPU | 1.45 | Uniaxial tensile tests | *Adv. Funct. Mater.*, **2025**, e10461. |
| ALH | 6.93 MPa | Uniaxial tensile tests | *Adv. Funct. Mater.*, **2024**, *34*, 2411384. |
| PMWPU-Bx | 243.2 MPa | Uniaxial tensile tests | *Small*, **2025**, *21*, 2410933. |
| PU-TA | 147 kPa | Uniaxial tensile tests | *Adv. Funct. Mater.*, **2025**, e17576. |
| PU-X | 1.30 | Uniaxial tensile tests | *Angew. Chem. Int. Ed.*, **2025**, *137*, e202421099. |
| NCoN | 1.1 | Uniaxial tensile tests | *Angew. Chem. Int. Ed.*, **2024**, *136*, e202318434. |
| TUEGx | 1.4 | Uniaxial tensile tests | *Science*, **2018**, *359*, 72-76. |
| PTBN6 | 4.25 | Uniaxial tensile tests | *Adv. Funct. Mater.*, **2022**, *32*, 2201959. |
| **POSS@PBA** | **1.78** | Uniaxial tensile tests | **This work** |

**5. Reference**

[1] W. Liu-Fu, H. Xiao, J. Chen, L. Cai, J. Yang, B. Xue, L. Lan, Y. Lai, J. F. Yin, P. Yin, *Nano Lett.* **2024**, *24*, 3307.

[2] K. Yue, C. Liu, K. Guo, X. Yu, M. Huang, Y. Li, C. Wesdemiotis, S. Z. D. Cheng, W. Bin Zhang, *Macromolecules* **2012**, *45*, 8126.

[3] X. Lin, M. X. Nie, H. Liu, D. L. Zhou, S. R. Fu, Q. Zhang, D. Han, Q. Fu, *Chem. Mater.* **2024**, *36*, 575.

[4] Y. Guo, M. Zhang, S. Wu, X. Wang, *Adv. Sci.* **2025**, *12*, e05122.

[5] L. Liu, Y. Deng, D. H. Qu, B. L. Feringa, H. Tian, Q. Zhang, *Angew. Chem. Int. Ed.* **2025**, *64*, e202424147.

[6] X. Wang, Y. Li, Y. Qian, H. Qi, J. Li, J. Sun, *Adv. Mater.* **2018**, *30*, 1803854.

[7] D. L. Zhou, J. H. Li, Q. Y. Guo, X. Lin, Q. Zhang, F. Chen, D. Han, Q. Fu, *Adv. Funct. Mater.* **2021**, *31*, 2102074.

[8] Y. Chu, W. Zhang, D. Liu, P. Chen, L. Huang, Y. You, S. Luan, C. Li, Y. Fu, *Adv. Funct. Mater.* **2025**, *10461*, 1.

[9] C. Zhou, N. Zhao, W. Liu, F. Hao, M. Han, J. Yuan, Z. Pan, M. Pan, *Small* **2025**, *21*, 2410933.

[10] S. Wang, C. Wong, Y. Pang, Y. Wu, Z. Yu, X. Zeng, R. Sun, *Adv. Funct. Mater.* **2025**, e17576.

[11] Y. Qian, F. Dong, S. Wang, Y. Jiang, X. Xu, H. Liu, *Angew. Chem. Int. Ed.* **2025**, *137*, e202421099.

[12] W. Niu, Z. Li, F. Liang, H. Zhang, X. Liu, *Angew. Chem. Int. Ed.* **2024**, *136*, e202318434.

[13] Z. H. Zhao, P. C. Zhao, Y. Zhao, J. L. Zuo, C. H. Li, *Adv. Funct. Mater.* **2022**, *32*, 2201959.

[14] Y. Yanagisawa, Y. Nan, K. Okuro, T. Aida, *Science.* **2018**, *359*, 72.
